# A New Red List of Endemic Vascular Plants of Iran Identifies a High Proportion of Threatened Species and Major Conservation Gaps

**DOI:** 10.1002/ece3.72394

**Published:** 2025-11-02

**Authors:** S. Khalvati, A. Talebi, M. Doostmohammadi, G. M. Schneeweiss, J. Noroozi

**Affiliations:** ^1^ Department of Biology Faculty of Sciences, Hakim Sabzevari University Iran; ^2^ School of Biology and Center of Excellence in Phylogeny of Living Organisms College of Science, University of Tehran Iran; ^3^ Department of Biology Faculty of Science, Shahid Bahonar University of Kerman Iran; ^4^ Department of Botany and Biodiversity Research University of Vienna Vienna Austria

**Keywords:** conservation gaps, endemic richness, Iran, IUCN, red list, Southwest Asia, threatened hotspots

## Abstract

Classifying the threat status of species using IUCN Red List categories is a crucial step in identifying endangered species and planning for their conservation. Iran, covering major parts of a global biodiversity hotspot in Southwest Asia, is climatically and topographically a heterogeneous country with a rich flora and a high concentration of endemics. Here, IUCN Red List criteria were applied to 2753 endemic Iranian vascular plants to assess their threat status. Moreover, threat hotspots of these categories and their conservation gaps were identified. Of all endemic species occurring in Iran, 2143 species (77.8%) are threatened; among those, 889 (32.3%) are critically endangered (CR), 875 (31.8%) are endangered (EN), and 379 (13.8%) are vulnerable (VU). Furthermore, 534 species (19.4%) were classified as least concern (LC) or near threatened (NT), and 76 species (2.8%) were classified as data deficient (DD). Based on the hotspots of threatened species, Threat Conservation Gaps, that is, hotspots of threatened species insufficiently covered by protected areas, were identified and found amounting to 58 percent. Hotspots of threatened species are restricted to the same five major mountain ranges of the Iranian Plateau identified as areas of endemism in previous studies. Our results provide an important basis for identifying areas of high conservation priorities, legislative decisions, and developing intervention strategies at the national level.

## Graphical abstract

Many endemic species are threatened by extinction due to anthropogenic activities, especially in global biodiversity hotspots with high number of endemics and high rate of destruction of natural habitats. IUCN Red List of threatened species is one of the most comprehensive data sources used in conservation biology to classify species based on their estimated extinction risk. In this study, IUCN Red List criteria were applied to all endemic Iranian plants, and hotspots of threatened species and their conservation gaps were identified.

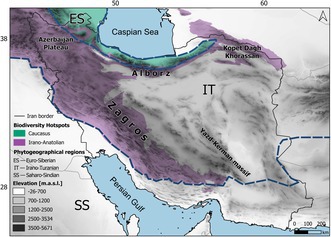

## 1 Introduction

A large number of endemic species are threatened by extinction due to anthropogenic activities (Mittermeier et al. 2005; Bellard et al. 2014). One of the main goals of conservation biology is to reduce the rate of their population decline and to prevent species extinction (Trombulak et al. 2004). Because of the large number of threatened taxa in global biodiversity hotspots (Myers et al. 2000; Mittermeier et al. 2005), an assessment of the extinction risk of these species, their geographic distribution, and of centres of endemism or hotspots in these areas is urgently needed (Possingham et al. 2002; Isaac et al. 2007; Carta et al. 2019). Hotspot analysis and gap analysis, which combine species diversity, endemism, and distributional characteristics in habitats, are two widely used approaches for determining conservation priorities (Myers 1988; Scott et al. 1993).

The International Union for Conservation of Nature (IUCN) Red List of threatened species (hereinafter Red List) is one of the most comprehensive and informative data sources in the field of conservation (Keith et al. 2015). IUCN criteria are commonly used to classify plant species based on their estimated risk of extinction and to assess global conservation status. This assessment provides baseline data and maps for policy makers to make appropriate decisions for the conservation of threatened species and the management of parks and protected areas at national and global levels. However, assessments of IUCN threat status of endemic species and their conservation status in some regions are largely missing (Di Marco et al. 2017), such as SW Asia.

Iran, characterized by heterogeneous topography and climate, supports a rich flora and fauna (Firouz 2005; Noroozi, Talebi, et al. 2019). The country harbors more than 8100 vascular plant species, of which approximately 30 percent are endemic (Noroozi, Talebi, et al. 2019). For Iranian endemics, areas and centres of endemism and conservation gaps calculated from those have already been studied (Noroozi, Naqinezhad, et al. 2019; Noroozi, Talebi, et al. 2019), but the threats to these endemics based on IUCN criteria and hotspots and conservation gaps calculated from these threat categories have not been explored so far.

**FIGURE 1:**
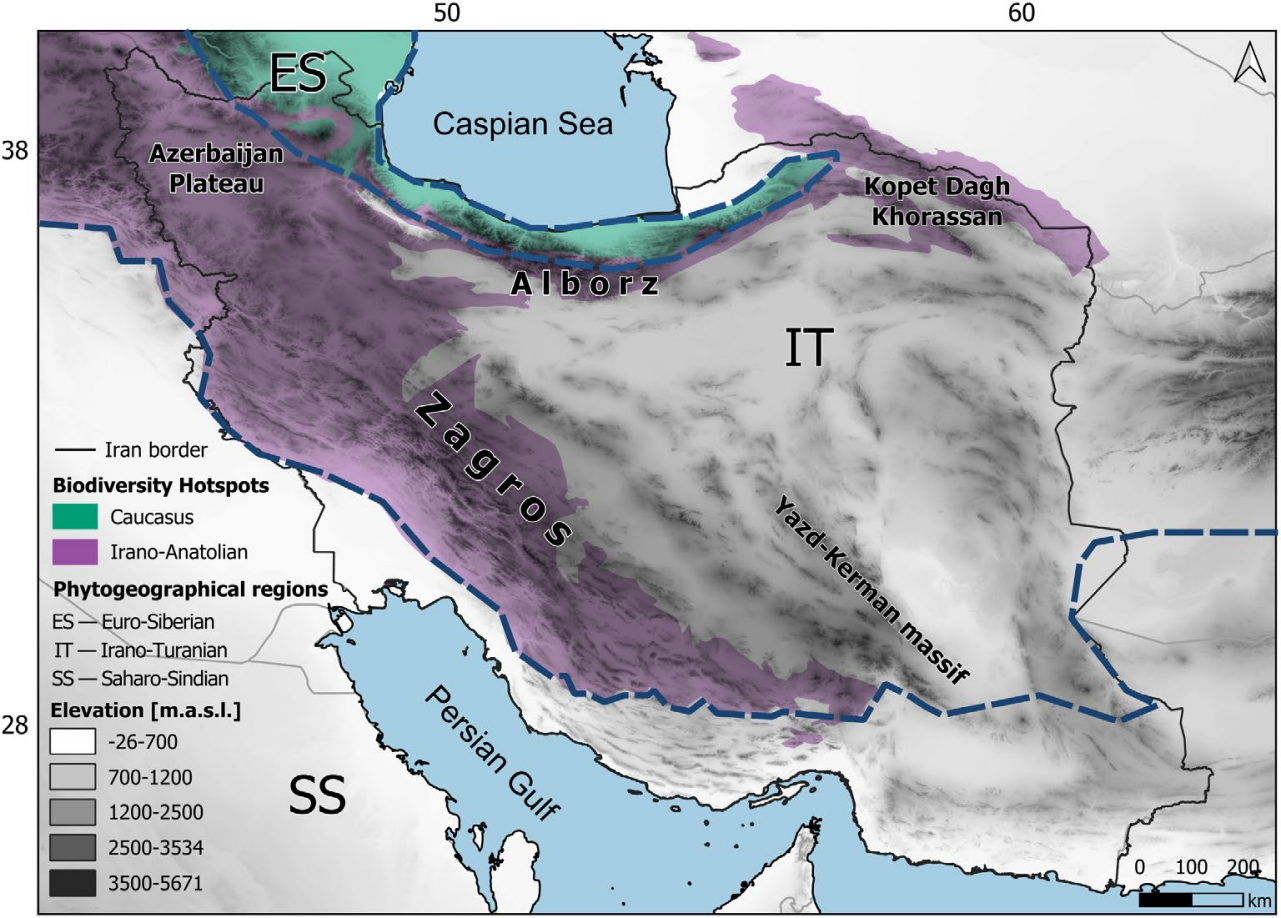
Topographic map of the study area (Iran) showing the phytogeographical regions, the global biodiversity hotspots, and the major mountain ranges.

Jalili and Jamzad (1999), in a preliminary survey of Iranian vascular plants in their Red Book, assessed 1727 taxa using the IUCN Red List criteria and classified 21 (1.2%) as endangered (EN), 432 (25%) as vulnerable (VU), 831 (48.1%) as of lower risk or of least concern (LR/LC), and 443 (25.6%) as data deficient (DD; Figure 1). The authors used data derived from herbarium specimens and field surveys, but do not explain how these were used to assign species to the IUCN 1994 Red List categories. Hence, it seems that the decision was subjective which can cause a considerable bias in the result of the classification. In addition, the authors have left out the category Critically Endangered (CR) intentionally in “order to be cautious in the study” (Jalili and Jamzad 1999, 4). Being more than 25 years old, the Red List of Jalili and Jamzad (1999) is in any case rather outdated. Therefore, a new study based on updated information and using objective methodology is necessary to assess the threat categories of the endemic species of this region.

Extent of occurrence (EOO) and area of occupancy (AOO) have been used by IUCN to produce conservation assessments. The extent of occurrence (EOO) is the spatial spread of the distribution area currently occupied by a taxon. It is not intended to be an estimate of actually occupied areas, but rather an indication of the spread of the extinction risk for the taxon. The area of occupancy (AOO) is the area of suitable habitat currently occupied by a taxon (IUCN 2012). The simplest approaches are to estimate EOO based on a convex envelope around occurrence localities, and AOO by summing the area of occupied grid cells at the defined resolution (IUCN 2012).

Our study has two aims. First, we want to assess the threat status of all endemic plant species of Iran according to the IUCN standard methods for geographic range, thus providing an updated threat assessment to be of use for conservation biologists. Second, we want to see if the endemic hotspots and conservation gaps of these regions, identified via analyses of centres of endemism (Noroozi, Naqinezhad, et al. 2019), are associated with threat hotspots. We expect that narrowly distributed species are more likely to be in higher threat categories, so that conservation gaps derived from endemic hotspots should be similar to those derived from threat hotspots.

## 2 Materials and Methods

### 2.1 Study Area

**FIGURE 2:**
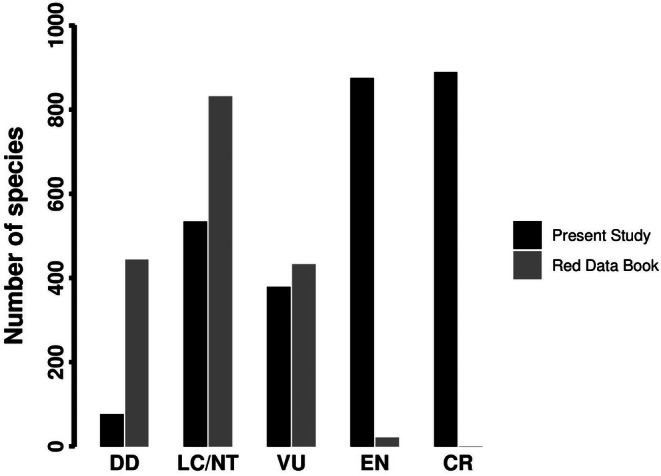
Comparison of Red List categories for the endemic flora of Iran in the present study and the Red Book of Iran (Jalili and Jamzad 1999).

Iran is located in Southwest Asia and covers an area of 1,648,195 km^2^, with a complex topography and a strong ecological heterogeneity (Figure 2). The different climatic zones of Iran include arid, semi‐arid, hyper‐arid, humid, semi‐humid, high‐humid, and Mediterranean climates (Zareiee 2014). Three phytogeographic regions and two global biodiversity hotspots intersect in this region (Zohary 1973; Mittermeier et al. 2005; Noroozi, Naqinezhad, et al. 2019). Iran's major mountain systems, including the Azerbaijan Plateau, Zagros, Alborz, Yazd–Kerman massif, and Kopet Dagh–Khorassan, represent centres of biodiversity and centres of endemism in the region (Farashi and Shariati 2017; Noroozi, Naqinezhad, et al. 2019). Their high topographic complexity and elevational gradients create diverse habitats, buffer extinction during climatic oscillations, and promote allopatric speciation (Djamali et al. 2012; Noroozi et al. 2018). A significant portion of the country lies within the Irano‐Turanian phytogeographic region and the Irano‐Anatolian global biodiversity hotspot (Zohary 1973; Noroozi, Naqinezhad, et al. 2019; Figure 1).

### 2.2 Species Checklist and Data Collection

Most of the distribution data on endemic vascular plant species (i.e., species having their range restricted to the study area) are from previous studies by Noroozi et al. (2018); Noroozi, Naqinezhad, et al. (2019); Noroozi, Talebi, et al. (2019), whose data were compiled mostly from the Flora Iranica (Rechinger 1963–2015) and the Flora of Iran (Assadi et al. 1989–2018). To supplement the data and to use the most recent floristic information, important monographs published after these floras were also included in our database (e.g., for mega‐diverse genera *Astragalus* L., *Cousinia* Cass., and *Allium* L., Maassoumi 2014; Kalouti et al. 2022; Fritsch et al. 2006, as well as many monographs about other taxonomic groups). We also used data from our own field surveys (since the early 2000s). In total, 2753 endemic vascular plant species with 25,077 geo‐referenced records were compiled for this analysis (Table S1). Species identification and nomenclature followed the Flora Iranica (Rechinger 1963–2015) and the Flora of Iran (Assadi et al. 1989–2018). The majority of the distribution records of the species taken from different floras had no coordinates. We used Google Earth to find the coordinates of the recorded points considering the locations and elevations of the records. We tried to be as precise as possible in this process. Subendemic species, that is, species with small occurrences also outside Iran, were not included in this study.

### 2.3 Data Assessment

The assessments were based on geographic range (Criteria B of IUCN 2024) including criterion B1 or extent of occurrence (EOO) and criterion B2 or area of occupancy (AOO). EOO and AOO were calculated with the “ConR” package (Dauby et al. 2017) of the R statistical computing environment (R Core Team 2020). In this framework, the categories Near Threatened (NT) and Least Concern (LC) are reported together as non‐threatened taxa (NT/LC; Dauby et al. 2017), and we followed this convention in our analyses. Due to the lack of demographic and population trend data (e.g., number of mature individuals, temporal monitoring) for most Iranian endemic species, we applied only IUCN Criterion B, which is based on range size metrics (EOO and AOO). In addition, our objective was to analyze spatial patterns of extinction risk and conservation gaps, which are directly linked to species distributions. This criterion is widely applied in large‐scale plant assessments, particularly in data‐poor regions (IUCN 2012; Dauby et al. 2017).

To identify the threat hotspots, we used grid cells in size of 0.5° × 0.5° (roughly 50 × 50 km; as used in previous studies, i.e., Noroozi, Talebi, et al. 2019). In total, entire Iran contained 712 cells, of which 568 cells contained values (i.e., the others did not harbor any endemic species). We examined Pearson correlation coefficients (*r*) of species richness between IUCN categories (CR, EN, and VU) as well as between each of them and the number of endemic species (endemic richness, ER) within each cell, using in‐built functions of R 4.2.1. The 5 percent, 10 percent, and 20 percent top‐most species‐rich cells of all threat categories (CR, EN, and VU) combined were identified as Threat Hotspots. Any Threat Hotspot with less than 10 percent of its area covered by protected areas was defined as a Threat Conservation Gap, using the same threshold value as in Noroozi et al. (2023). Data on the management quality of protected areas were not available; thus, this aspect could not be included in the calculation of Threat Conservation Gaps.

## 3 Results

From a total of 2753 endemic species of Iran, 2143 (77.8%) were classified as threatened, including 889 (32.3%) as CR, 875 (31.8%) as EN, and 379 (13.8%) as VU. Moreover, 534 (19.4%) species were classified as LC or NT (Table 1). The proportion of the different categories differs strongly from those in the Red Book of Iran (Jalili and Jamzad 1999), the differences being most pronounced in categories CR and EN that changed from 0 and 21 species in the Red Book to 720 and 796 species, respectively, in our work (Figure 2).

**TABLE 1:** Number and percentage of species in each IUCN category.

	IUCN category	Number of species/%
Threatened	Critically Endangered (CR)	889/32.3
	Endangered (EN)	875/31.8
	Vulnerable (VU)	379/13.8
	Least Concern (LC) or Near Threatened (NT)	534/19.4
	Data Deficient (DD)	76/2.8
	Sum:	2753/100

*Note:* Color codes follow the official IUCN scheme (CR–red, EN–orange, VU–yellow, LC/NT–green, DD–grey).

**FIGURE 3:**
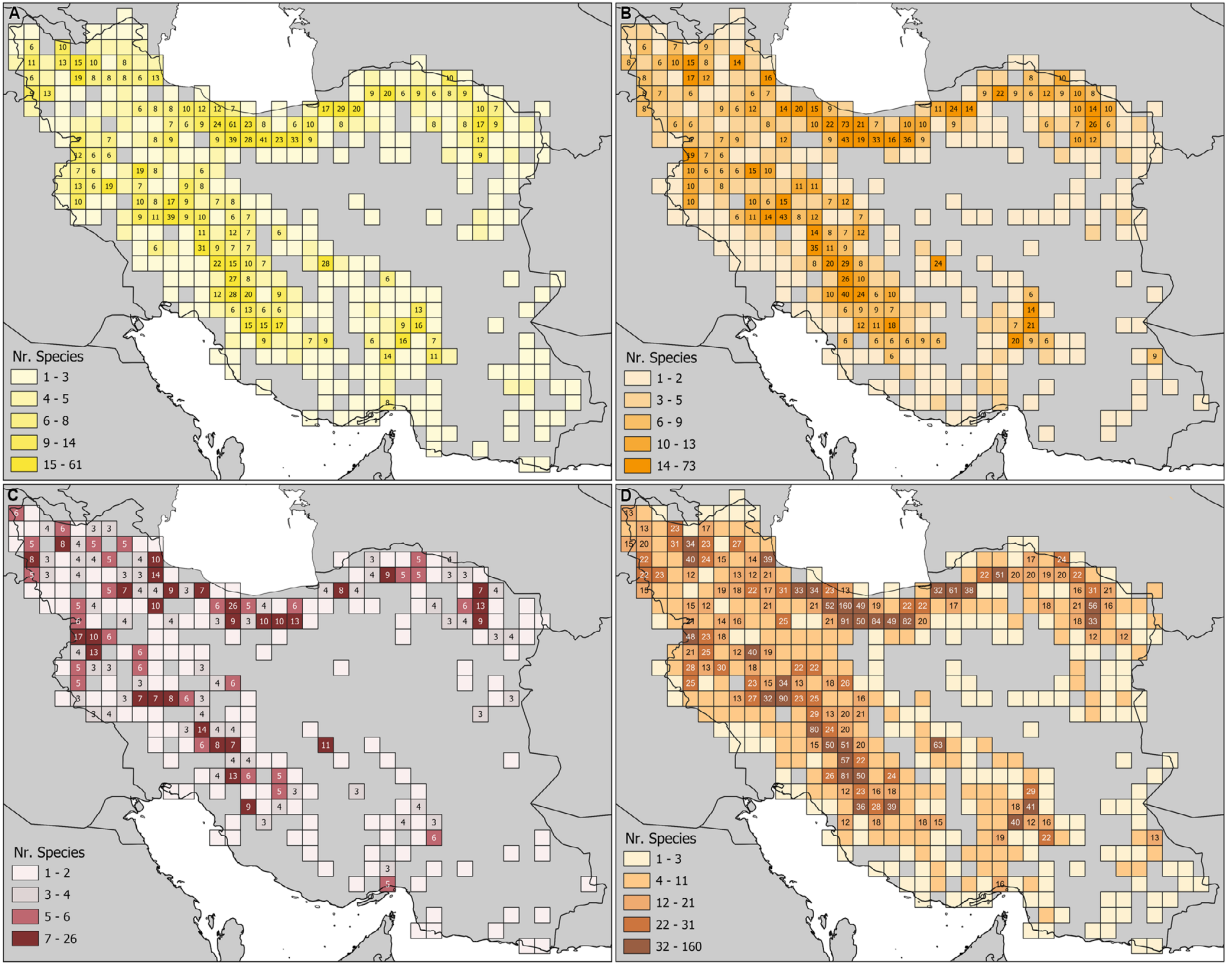
Richest cells of (A) VU, (B) EN, (C) CR, and (D) threatened, that is VU + EN + CR. In all four graphs, the first three size classes correspond to the top 5%, top 5%–10%, and top 10%–20% richest cells, respectively.

Species richness in category VU ranged from 0 to 61 species per cell (Figure 3A), that in category EN ranged from 0 to 73 species per cell (Figure 3B), and that in category CR ranged from 0 to 26 species per cell (Figure 3C). Species richness of all three threat categories combined ranged from 0 to 160 species per cell (Figure 3D). Pearson correlation coefficients of species richness among the four categories ranged from *r* = 0.63 (CR with NT/LC) to *r* = 0.93 (EN with VU; Table 2). Each category was strongly correlated with endemic richness (*r* = 0.92 with EN, *r* = 0.94 with VU and *r* = 0.97 with NT/LC), but more weakly correlated with CR (*r* = 0.75; Table 2).

**TABLE 2:** Pearson correlation coefficients (*r*) of species richness of the four IUCN categories and of endemic richness (ER) across grid cells; all correlations are highly significant (*p* < 0.001).

	EN	VU	NT or LC	ER
CR	0.81	0.76	0.63	0.75
EN		0.93	0.82	0.92
VU			0.85	0.94
NT or LC				0.97

Abbreviations: CR, Critically Endangered richness; EN, Endangered richness; ER, Endemic Richness; NT or LC, Near Threatened or Least Concern richness; VU, Vulnerable richness.

**FIGURE 4:**
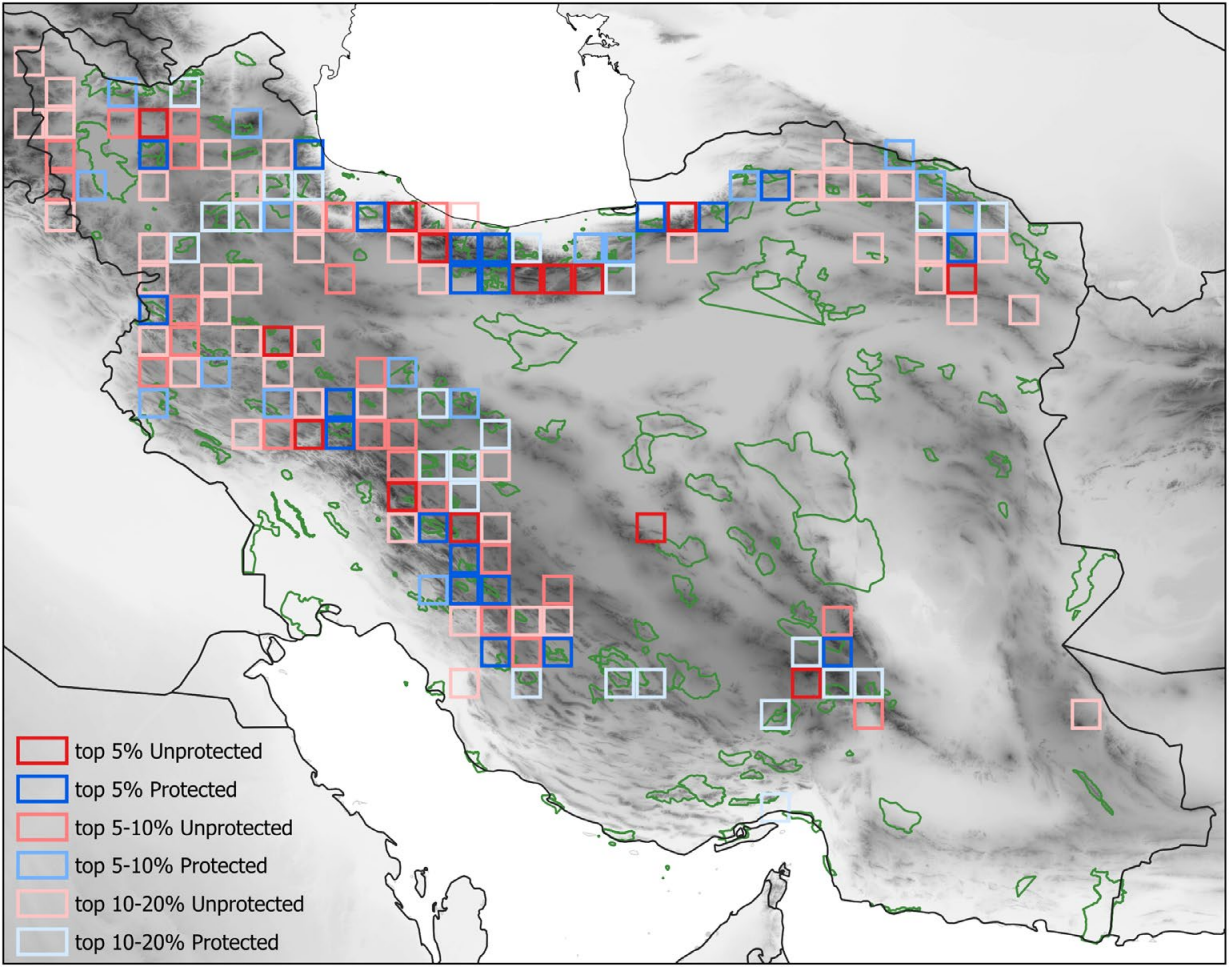
Threat Hotspots (top 5%, top 5%–10%, top 10%–20% of the most species‐rich cells) with more (hues of blue) or less (hues of red) than 10% of the cell's area being protected, the latter (i.e., the red cells) constituting Threat Conservation Gaps. Protected areas or areas under conservation, including national parks, wildlife refuges, and other protected areas in the study area are shown in green color.

We identified 35, 39, and 70 cells as Threat Hotspots for the top 5%, top 5%–10% and top 10%–20% richest cells of threatened species, respectively (Figure 4; Table 3). About 58% of these Threat Hotspots were identified as Threat Conservation Gaps (Figure 4; Table 3). The Threat Hotspots were concentrated in the five high mountain systems of the region. Zagros harbored 59 Threat Hotspots with 33 (61%) Threat Conservation Gaps, Alborz 23 Threat Hotspots with 11 (48%) Threat Conservation Gaps, Kopet Dagh 19 Threat Hotspots with 12 (63%) Threat Conservation Gaps, Azerbaijan Plateau 32 Threat Hotspots with 20 (63%) Threat Conservation Gaps, and Yazd‐Kerman 9 Threat Hotspots with 4 (44%) Threat Conservation Gaps. Outside the mentioned mountain ranges, only a single Threat Hotspot, which is also a Threat Conservation Gap, was found.

**TABLE 3:** Number of Threat Hotspots and number (percentage) of Threat Conservation Gaps.

	Threat hotspots	Threat conservation gaps (%)
Top 5% hotspots	35	14 (40)
Top 5%–10% hotspots	39	23 (59)
Top 10%–20% hotspots	70	47 (67)

## 4 Discussion

The Red List presented here is one of the most complete IUCN assessments of vascular plants of West Asian countries and the Irano‐Anatolian area, a global biodiversity hotspot. It is important to note, however, that the species assessed here have not yet been formally published on the IUCN global Red List. Still, this work provides a robust national‐scale baseline to support future studies and make the data more accessible to conservation practitioners. The big difference between the number of species in each IUCN category obtained here and those reported in the Red Book of Iran (Jalili and Jamzad 1999) likely is due to differences in the methods used. While we applied the standard methodology of IUCN, which is based on the distribution range of a species (IUCN 2012), the assessment in the Red Book seems to be subjective without standard methodology, including not considering the category CR at all. At the same time, it is important to acknowledge that in 1999 the available tools and methods for large‐scale Red List assessments were much more limited. The Red Book, therefore, represents a pioneering effort that was highly relevant for conservation in its time and today provides a valuable baseline for comparison with more recent and comprehensive assessments.

Only very few species were categorized in EN by Jalili and Jamzad (1999), which is unexpected for a region harboring a high number of range‐restricted species known from a few records or even the type locality only (Noroozi, Talebi, et al. 2019). Most of the locally endemic species with only a few records were categorized as DD by Jalili and Jamzad (1999), whereas here such species were categorized as CR or EN. We are aware that our knowledge about the distribution and biology of the rare and locally endemic species is far from complete and that in the future new localities may be found, but a long history of floristic exploration (Rechinger 1963–2015; Assadi et al. 1989–2018) and the large number of recent local floristic and vegetation studies render it unlikely that our knowledge on the distribution of range‐restricted species is substantially underestimated due to the lack of data. Therefore, we believe that the narrowly distributed species should be categorized as threatened species according to updated methodologies of the IUCN instead of being treated as DD.

This discrepancy in assessment between Jalili and Jamzad (1999) and our study can be partly explained by methodological differences. At that time, the IUCN criteria and tools available for Red List assessments were much more limited, and Jalili and Jamzad (1999) relied heavily on expert judgment, causing many narrowly distributed species with few records to be placed in the DD category. In contrast, our current assessment follows the updated IUCN methodology (IUCN 2012), which explicitly recommends assigning species with highly restricted ranges to threatened categories (CR or EN), even when detailed biological information may be lacking. In addition to methodological differences, part of the discrepancy with Jalili and Jamzad (1999) reflects changes in taxonomic coverage and updates over the past 25 years: we assessed 2753 endemic species versus 1727 species (mostly endemics, but partly non‐endemics rare in Iran) in their list, amounting to about 60 percent more species assessed in our study. Much of this increase arises from newly described or taxonomically revised, local endemics particularly within mega‐diverse genera such as *Astragalus*, *Cousinia*, and *Allium*, which tend to meet Criterion B thresholds. Moreover, Jalili and Jamzad (1999) intentionally omitted CR, which complicates direct comparison between their and our assessment. Because our assessment is range‐based and time‐series population data are largely unavailable, we cannot disentangle the relative contributions to the discrepancies in threat categories of genuine decline versus methodological and data issues (taxonomic updates); we, therefore, frame the comparison with the list of Jalili and Jamzad (1999) as indicative rather than as strictly like‐for‐like.

IUCN categories are strongly correlated with each other and also with endemic richness, although to a lesser extent for category CR (Table 2). The correlation between the number of endemic species and those classified as CR was lower (*r* = 0.75) compared to EN (*r* = 0.92) and VU (*r* = 0.94). This can be due to the smaller sample size of CR species, which reduces the robustness of the correlation coefficient. Additionally, many of the CR taxa have been described as new species in the last decades that are concentrated in certain regions due to targeted botanical surveys by specific researchers. For example, many newly described species of genera such as *Alchemilla*, *Centaurea*, and *Onosma* have been reported mainly from specific areas in western Iran. Such geographically biased discovery patterns may not align with the overall distribution of endemic species, which could explain the lower observed correlation.

Our data largely support the hypothesis that hotspots of endemic species strongly correspond to hotspots of rare and threatened species (Orme et al. 2005; Hurdu et al. 2016). Yet, the distribution of the Hotspots, inferred from indices of endemicity (Noroozi, Naqinezhad, et al. 2019) is similar to the distribution of Threat Hotspots, inferred from IUCN threat categories. The identified Threat Hotspots are clearly restricted to the five major mountain ranges of the Iranian Plateau, that is, Azerbaijan Plateau, Alborz, Zagros, Yazd‐Kerman, and Kopet Dagh‐Khorassan (Figure 4). These five mountain systems have been repeatedly identified as areas of endemism (Noroozi et al. 2018, 2021, 2023; Noroozi, Talebi, et al. 2019). This suggests that the identification of endemic hotspots or centres of endemism (using different biodiversity indices) can be a very useful method to approximate hotspots of threatened species. Only one Threat Hotspot is identified outside of the mentioned five mountain ranges, which is an isolated volcanic mountain in south east Iran (Taftan Mt., 3941 m a.s.l.). The large elevational range of this mountain supports heterogeneous habitats in different elevations and aspects. We already know that the topographic complexity and elevational amplitudes are associated with endemic diversity in this region (Noroozi et al. 2018). On the other hand, this mountain is highly isolated from other high mountains, and usually isolated regions are rich in endemic species (Steinbauer et al. 2016).

The number of threatened species is very high, being about three quarters of the many endemic species of Iran. This is likely due to the high proportion of range‐restricted species in the region (see also Noroozi, Talebi, et al. 2019; Noroozi et al. 2023), which is considered to be the result of heterogeneous climate and topography, isolation of habitats, and a rather low rate of species extinction in the last glacial periods (Noroozi et al. 2018; Zohary 1973). Potential threats resulting from range restriction are now exacerbated by several more or less anthropogenic factors. Applying the full set of IUCN criteria (A–E) would allow the inclusion of the impact of those factors, and it can be expected that using all criteria instead of only Criterion B of the IUCN Red List likely would increase the number of species classified as threatened.

**FIGURE 5:**
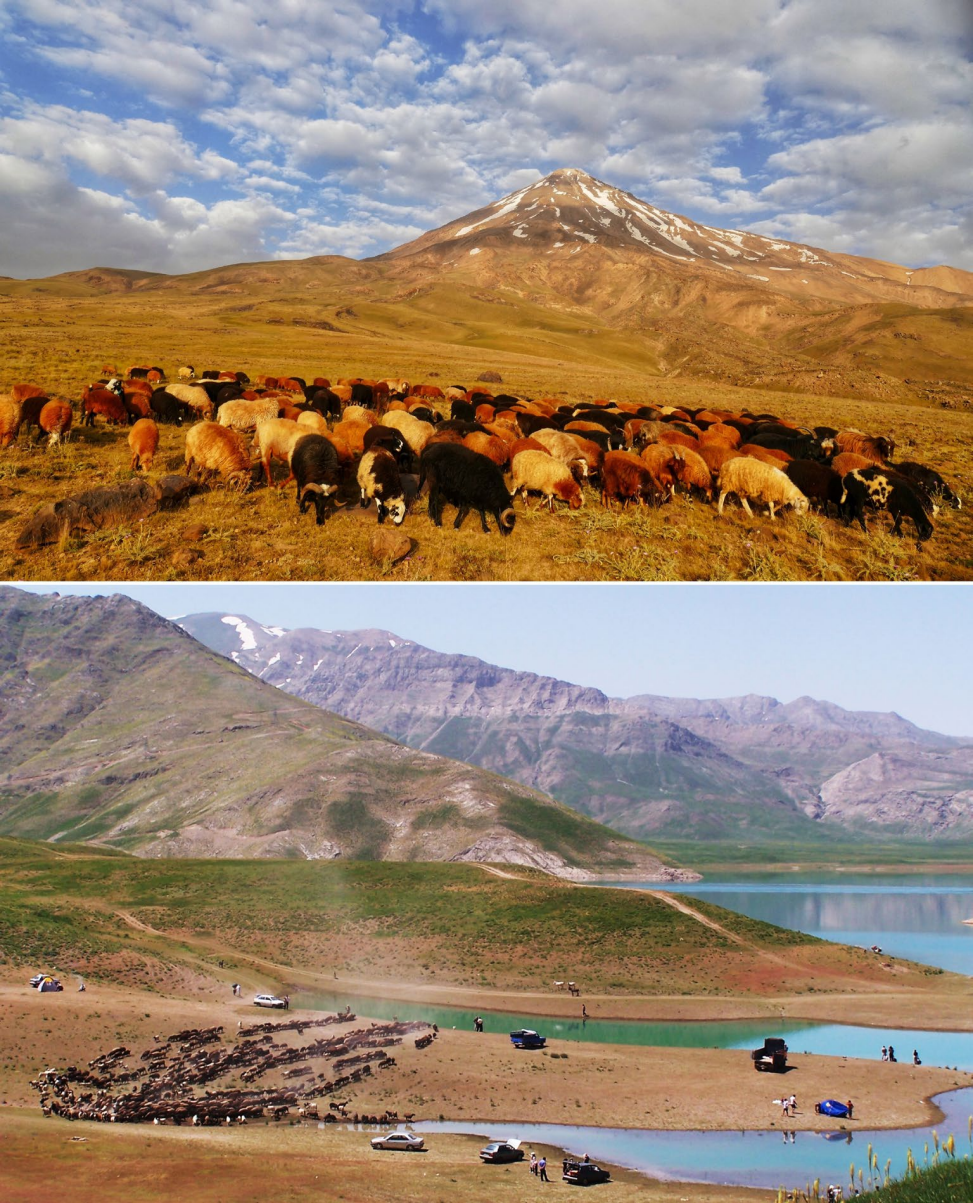
Overgrazing in Lar National Park, 2500–2700 m a.s.l., Central Alborz (photos: J.N.).

One of those anthropogenic factors is climate change, which may be stronger in the Middle East compared to other regions (the mean temperature is increasing almost twice as fast as the global average, and the precipitation regime is strongly changing: Zittis et al. 2022). This impact is more intense for alpine species, because their upwards migrations are usually limited by the lack of alternative suitable habitats at higher elevations (Dullinger et al. 2012; Gottfried et al. 1999). Besides global warming, endemic and rare species of Iran suffer from intensive grazing and trampling by sheep and goats that graze in all elevational zones and even reach the alpine habitats by the end of summer. Iran harbors the fifth largest population of sheep in the world and population sizes are still increasing (Jowkar et al. 2016). Even in some protected areas, such as Lar National Park (Figure 5), we can see huge herds of sheep and goats. Overgrazing clearly modified the species composition in these regions and caused a high abundance of grazing‐resistant plants such as different species of *Euphorbia*, *Cousinia*, *Papaver*, and thorn‐cushion plants. Outside the alpine zone, dam constructions have resulted in the drying up of several downstream lakes and wetlands and increased the rate of desertification (Akhani and Rudov 2018). Wildfires, particularly in rangelands and forests, are another source of threat. About 15,000 ha of Iranian forests burn each year (Kheshti 2020), destroying natural vegetation types in Zagros and Alborz.

Almost all known endemic species of Iran have been assessed, with only a small number currently classified as Data Deficient (DD); for those species more studies are needed to be able to evaluate their conservation status. Conservation priority should be given to establishing new protected areas in Threat Hotspots, particularly in identified Threat Conservation Gaps. Additionally, conservation efficiency and effectiveness of the existing protected areas of the region which are in general lower than the global average (Farashi and Shariati 2017; Kolahi et al. 2013), need to be increased. Our identification of Threat Conservation Gaps is solely based on the spatial extent of protected areas, without considering differences in management regimes or enforcement effectiveness. Previous studies have shown that such qualitative factors can significantly influence conservation outcomes (e.g., Geldmann et al. 2019). Therefore, while the < 10 percent coverage threshold provides a practical and objective metric to identify conservation gaps, it may overestimate or underestimate the actual conservation deficits in some areas. Improving data availability and incorporating management effectiveness assessments in future studies will be crucial to refine conservation prioritization.

## Author Contributions

**S. Khalvati:** formal analysis (lead), investigation (equal), methodology (lead), software (lead), visualization (lead), writing – original draft (lead). **A. Talebi:** data curation (equal), writing – review and editing (equal). **M. Doostmohammadi:** data curation (equal), writing – review and editing (equal). **G. M. Schneeweiss:** writing – review and editing (equal). **J. Noroozi:** conceptualization (lead), data curation (lead), funding acquisition (lead), methodology (lead), project administration (lead), supervision (lead), writing – original draft (supporting), writing – review and editing (lead).

## Conflicts of Interest

The authors declare no conflicts of interest.

## Supporting information


**Table S1:** ece372394‐sup‐0001‐TableS1.pdf.

## Data Availability

All data generated or analyzed during this study are included in this published article or in Supporting Information files.
