# Supplementary material for: A New Red List of Endemic Vascular Plants of Iran Identifies a High Proportion of Threatened Species and Major Conservation Gaps
Source: Ecol Evol. 2025 Nov 2;15(11):e72394. doi: 10.1002/ece3.72394 (PMC12579972; doi:10.1002/ece3.72394)
Supplement: Supplementary file 1 — Table S1: ece372394‐sup‐0001‐TableS1.pdf. [file ECE3-15-e72394-s001.pdf]

Supplementary file for:

Khalvati S, Talebi A, Doostmohammadi M, Schneeweiss GM, Noroozi J. A new Red list of endemic vascular plants of Iran identifies a high proportion of threatened species and major conservation gaps. Ecology and Evolution

Table S1. Endemic species of Iran and their IUCN Threat Categories, number of records, EOO values, and AOO values.

Species	Family	IUCN Threat Category	Record nr.	EOO (km^2)	AOO (km^2)
Acantholimon acmostegium Boiss. & Buhse	Plumbaginaceae	LC or NT	33	108840	128
Acantholimon alavae Rech.f. & Schiman-Czeika	Plumbaginaceae	EN	4	1758	16
Acantholimon albocalycinum Assadi & Mirtadzadini	Plumbaginaceae	EN	2	0	8
Acantholimon asphodelinum Mobayen	Plumbaginaceae	LC or NT	15	19175	56
Acantholimon assadii Mirtadz. & Bordbar	Plumbaginaceae	CR	1	0	4
Acantholimon austro-iranicus Rech.f. & Schiman-Czeika	Plumbaginaceae	VU	10	10408	40
Acantholimon bakhtiaricum Assadi	Plumbaginaceae	CR	1	0	4
Acantholimon bodeanum Bunge	Plumbaginaceae	LC or NT	27	83447	104
Acantholimon brachystachyum Boiss. ex Bunge	Plumbaginaceae	VU	9	137457	36
Acantholimon bromifolium Boiss. ex Bunge	Plumbaginaceae	LC or NT	24	71206	96
Acantholimon cephalotoides Rech.f. in DC.	Plumbaginaceae	VU	9	13323	36
Acantholimon chlorostegium Rech.f. & Schiman-Czeika	Plumbaginaceae	VU	10	32966	40
Acantholimon collare Koeie & Rech.f.	Plumbaginaceae	VU	8	42073	32
Acantholimon cupreo-olivascens Rech.f. & Schiman-Czeika	Plumbaginaceae	EN	4	5260	12
Acantholimon demavendicum Bornm.	Plumbaginaceae	LC or NT	15	20061	56
Acantholimon densiflorum Assadi	Plumbaginaceae	EN	2	0	8
Acantholimon eschkerense Boiss. & Hausskn.	Plumbaginaceae	LC or NT	15	23663	60
Acantholimon flabellum Assadi	Plumbaginaceae	EN	4	3601	16
Acantholimon flexuosum Boiss. & Hausskn. ex Bunge	Plumbaginaceae	LC or NT	25	240601	92
Acantholimon gadukense Mobayen	Plumbaginaceae	CR	2	0	4

Acantholimon glabratum Assadi	Plumbaginaceae	EN	2	0	8
Acantholimon gorganense Mobayen	Plumbaginaceae	EN	5	5325	20
Acantholimon gulistanum Bunge	Plumbaginaceae	CR	1	0	4
Acantholimon haesarensis Bornm. ex Rech.f. & Schiman-Czeika	Plumbaginaceae	EN	3	50	12
Acantholimon hamadannicum Assadi & Mahmoodi	Plumbaginaceae	EN	3	706	12
Acantholimon hormozganense Assadi	Plumbaginaceae	CR	1	0	4
Acantholimon horridum Bunge	Plumbaginaceae	EN	5	1028	20
Acantholimon hystrix Stapf	Plumbaginaceae	CR	1	0	4
Acantholimon incomptum Boiss. & Buhse	Plumbaginaceae	DD	NA	NA	NA
Acantholimon kermanense Assadi & Mirtadz.	Plumbaginaceae	EN	5	22117	20
Acantholimon melananthum Boiss.	Plumbaginaceae	EN	9	11597	32
Acantholimon mirtadzadinii Assadi	Plumbaginaceae	EN	3	69	12
Acantholimon mishaudaghense Mobayen	Plumbaginaceae	CR	3	12	12
Acantholimon mobayenii Assadi & Ghahreman	Plumbaginaceae	CR	1	0	4
Acantholimon modestum Bornm. ex Rech.f. & Schiman-Czeika	Plumbaginaceae	LC or NT	23	50376	84
Acantholimon moradii Assadi	Plumbaginaceae	EN	2	0	8
Acantholimon nigricans Mobayen	Plumbaginaceae	EN	7	4649	24
Acantholimon oliganthum Boiss.	Plumbaginaceae	VU	10	117898	36
Acantholimon ophiocladus Rech.f. & Schiman-Czeika	Plumbaginaceae	EN	2	0	8
Acantholimon quinquelobum Bunge	Plumbaginaceae	LC or NT	12	36552	48
Acantholimon restiaceum Bunge	Plumbaginaceae	EN	5	12119	20
Acantholimon rhodopolius Rech.f. & Schiman-Czeika	Plumbaginaceae	EN	3	1008	12
Acantholimon saadii Assadi & Zeraatkar	Plumbaginaceae	CR	1	0	4
Acantholimon scabrellum Boiss. & Hausskn.	Plumbaginaceae	LC or NT	15	46931	56
Acantholimon schahrudicum Bunge	Plumbaginaceae	LC or NT	27	204302	104
Acantholimon schirazianum Boiss.	Plumbaginaceae	EN	5	4767	20
Acantholimon scirpinum Bunge	Plumbaginaceae	VU	6	32653	24
Acantholimon scorpius (Jaub. & Spach) Boiss.	Plumbaginaceae	VU	7	283491	28
Acantholimon senganense Bunge	Plumbaginaceae	LC or NT	17	151522	68
Acantholimon serotinus Rech.f. & Schiman-Czeika	Plumbaginaceae	VU	8	62728	32

Acantholimon sirchense Assadi & Mirtadzadini	Plumbaginaceae	EN	5	404	16
Acantholimon talagonicum Boiss. in DC.	Plumbaginaceae	LC or NT	23	71146	88
Acantholimon termei Rech.f. & Schiman-Czeika	Plumbaginaceae	CR	1	0	4
Acantholimon tomentellum Boiss.	Plumbaginaceae	VU	8	6838	32
Acantholimon tragacanthinum (Jaub. & Spach) Boiss.	Plumbaginaceae	EN	5	17417	20
Acantholimon wendelboi Rech.f. & Schiman-Czeika	Plumbaginaceae	VU	10	95686	40
Acantholimon wilhelminae Rech.f.	Plumbaginaceae	CR	1	0	4
Acantholimon zaeifii Assadi	Plumbaginaceae	VU	11	6911	40
Acanthophyllum andersenii Rech.f. & Schiman-Czeika	Caryophyllaceae	CR	1	0	4
Acanthophyllum chloroleucum Rech.f. & Aell.	Caryophyllaceae	EN	2	0	8
Acanthophyllum crassifolium Boiss.	Caryophyllaceae	LC or NT	48	403294	192
Acanthophyllum ejtehadii Mahmoudi & Vaezi	Caryophyllaceae	EN	3	49	12
Acanthophyllum leucostegium Schiman-Czeika	Caryophyllaceae	VU	8	166482	32
Acanthophyllum pachycephalum Schiman-Czeika	Caryophyllaceae	LC or NT	19	156969	76
Acanthophyllum yasamin-nassehiae Joharchi & Pirani	Caryophyllaceae	CR	1	0	4
Acer iranicum Mohtash. & Rastegar	Sapindaceae	CR	1	0	4
Acer mazandaranicum Amini	Sapindaceae	CR	4	12	12
Acer zarei Amini	Sapindaceae	CR	1	0	4
Achillea aucheri Boiss.	Asteraceae	EN	6	37265	24
Achillea callichroa Boiss.	Asteraceae	CR	1	0	4
Achillea eriophora DC.	Asteraceae	LC or NT	37	443294	148
Achillea kellalensis Boiss. & Hausskn.	Asteraceae	EN	5	8307	20
Achillea oxyodonta Boiss.	Asteraceae	VU	12	12750	48
Achillea pachycephala Rech.f.	Asteraceae	VU	6	46639	24
Achillea talagonica Boiss.	Asteraceae	LC or NT	34	411349	136
Aconitum iranshahrii H. Riedl	Ranunculaceae	CR	1	0	4
Adonis globosa C. Steinb. ex Rech.f.	Ranunculaceae	VU	8	50607	32
Aeluropus laciniatus Khodashenas	Poaceae	EN	2	0	8
Aeluropus peterganicus Khodashenas	Poaceae	EN	4	2832	16
Aethionema alpinum Moazzeni & Noroozi	Boraginaceae	EN	4	66951	16

Aethionema cephalanthum (Bornm.) Bornm.	Brassicaceae	EN	2	0	4
Aethionema erinaceum (Boiss.) Khosravi & Mumm.	Brassicaceae	EN	2	0	8
Aethionema sabzevaricum Khosravi & Joharchi	Brassicaceae	EN	3	288	8
Aethionema semnanensis Mozaffarian	Brassicaceae	EN	6	3873	24
Aethionema stenopterum Boiss.	Brassicaceae	VU	12	15513	44
Aethionema umbellatum (Boiss.) Bornm.	Brassicaceae	VU	9	38860	36
Aethionema zagricum Moazzeni & Mahmoodi	Boraginaceae	EN	3	3533	12
Agropyron afghanicum Melderis in Bor	Poaceae	EN	4	343411	16
Agropyron brachyphyllum Boiss. & Hausskn. ex Boiss.	Poaceae	EN	2	0	4
Agropyron bulbosum Boiss.	Poaceae	CR	1	0	4
Agropyron gentryi Melderis in Bor	Poaceae	CR	1	0	4
Agrostis gariana Taheri	Poaceae	CR	1	0	4
Ajania semnanensis Sonboli	Asteraceae	EN	5	1699	20
Ajuga chamaecistus Ging. ex Benth.	Lamiaceae	LC or NT	235	761373	912
Ajuga saxicola Assadi & Jamzad	Lamiaceae	CR	1	0	4
Albraunia foveopilosa Speta	Plantaginaceae	LC or NT	16	19332	60
Alcea acaulis (Cav.) Alef.	Malvaceae	DD	NA	NA	NA
Alcea arbelensis Boiss. & Hausskn.	Malvaceae	DD	NA	NA	NA
Alcea assadii Pakravan	Malvaceae	CR	1	0	4
Alcea fasciculiflora Zohary	Malvaceae	DD	NA	NA	NA
Alcea flavovirens (Boiss. & Buhse) Iljin	Malvaceae	EN	5	34465	20
Alcea ghahremanii Pakravan & Assadi	Malvaceae	CR	1	0	4
Alcea glabrata Alef.	Malvaceae	LC or NT	65	303120	220
Alcea gorganica (Rech.f.	Malvaceae	VU	9	19315	36
Alcea ilamica Pakravan	Malvaceae	CR	1	0	4
Alcea iranshahrii Pakravan	Malvaceae	CR	1	0	4
Alcea koelzii I.Riedl	Malvaceae	VU	8	110611	32
Alcea kurdica (Schlecht.) Alef.	Malvaceae	LC or NT	11	129064	44
Alcea loftusii (Baker) Zohary	Malvaceae	EN	4	13862	16
Alcea mazandaranica Pakravan & Ghahreman	Malvaceae	CR	1	0	4

Alcea mozaffarianii Ghahreman	Malvaceae	CR	1	0	4
Alcea persarum Bornm.	Malvaceae	EN	3	477062	12
Alcea schirazana Alef	Malvaceae	LC or NT	17	72625	64
Alcea semnanica Pakravan	Malvaceae	CR	1	0	4
Alcea tarica Pakravan & Ghahreman	Malvaceae	VU	9	16546	32
Alcea teheranica Parsa	Malvaceae	EN	3	1116	12
Alcea wilhelminae I. Riedl	Malvaceae	LC or NT	20	36838	64
Alchemilla amardica Rothm.	Rosaceae	EN	4	369	16
Alchemilla angustiloba S.E.Fröhner	Rosaceae	CR	1	0	4
Alchemilla chloroporphyrea S.E.Fröhner & Faghir	Rosaceae	CR	1	0	4
Alchemilla citrina S. E. Fröhner	Rosaceae	LC or NT	16	48744	64
Alchemilla condensa S. E. Fröhner	Rosaceae	EN	4	11720	16
Alchemilla crassiflora S.E.Fröhner & Faghir	Rosaceae	CR	1	0	4
Alchemilla cyclocteis S.E.Fröhner & Faghir	Rosaceae	CR	1	0	4
Alchemilla diversistipula S.E.Fröhner & Faghir	Rosaceae	CR	1	0	4
Alchemilla farinosa S. E. Fröhner	Rosaceae	EN	4	2276	16
Alchemilla fluminea S. E. Fröhner	Rosaceae	EN	5	16494	20
Alchemilla gigantodus S. E. Fröhner	Rosaceae	VU	8	60148	32
Alchemilla hessii Bothm.	Rosaceae	VU	13	27704	48
Alchemilla mazandarana Naqinezhad & S. E. Fröhner	Rosaceae	EN	4	351	16
Alchemilla melancholica S. E. Fröhner	Rosaceae	VU	11	13475	44
Alchemilla microscopica S. E. Fröhner	Rosaceae	EN	4	19700	16
Alchemilla ochropoda S.E.Fröhner & Faghir	Rosaceae	CR	1	0	4
Alchemilla ochropodoides S.E.Fröhner & Faghir	Rosaceae	CR	1	0	4
Alchemilla paucidens S.E.Fröhner & Faghir	Rosaceae	CR	1	0	4
Alchemilla pectiniloba S. E. Fröhner	Rosaceae	EN	4	5895	16
Alchemilla plicatissima S. E. Fröhner	Rosaceae	EN	3	5563	12
Alchemilla polioporphyrea S.E.Fröhner & Faghir	Rosaceae	CR	1	0	4
Alchemilla rechingeri Rothm.	Rosaceae	CR	3	12	12
Alchemilla rotundata S.E.Fröhner & Faghir	Rosaceae	CR	1	0	4

Alchemilla scutellata S.E.Fröhner & Faghir	Rosaceae	CR	1	0	4
Alchemilla subpotentilla S.E.Fröhner & Faghir	Rosaceae	CR	1	0	4
Alchemilla surculosa S. E. Fröhner	Rosaceae	EN	2	0	8
Alkanna assadii Khajoei Nasab & Zeraatkar	Boraginaceae	DD	NA	NA	NA
Alkanna bracteosa Boiss.	Boraginaceae	LC or NT	27	143150	92
Alkanna frigida Boiss.	Boraginaceae	LC or NT	26	265437	104
Alkanna semiromica Ranjbar & Khalvati	Boraginaceae	CR	1	0	4
Allium abbasii R.M Fritsch	Amaryllidaceae	CR	1	0	4
Allium akaka S.G. Gmel. ex Schult. & Schult.f.	Amaryllidaceae	LC or NT	17	28732	64
Allium aladaghense Memariani & Joharchi	Amaryllidaceae	EN	4	2280	16
Allium alamutense Razyfard	Amaryllidaceae	VU	7	10367	28
Allium assadii Seisums	Amaryllidaceae	EN	5	5322	20
Allium assadiiA1396	Amaryllidaceae	CR	1	0	4
Allium austroiranicum R.M. Fritsch	Amaryllidaceae	LC or NT	71	195164	272
Allium autumniflorum F.O. Khass. & Akhani	Amaryllidaceae	CR	1	0	4
Allium aznavense R.M.Fritsch	Amaryllidaceae	CR	1	0	4
Allium bakhtiaricum Regel	Amaryllidaceae	LC or NT	21	106266	80
Allium bisotunense R.M. Fritsch	Amaryllidaceae	EN	4	2412	16
Allium brachyodon Boiss.	Amaryllidaceae	EN	3	18908	12
Allium brachyscapum Vved.	Amaryllidaceae	EN	5	428169	20
Allium breviscapum Stapf	Amaryllidaceae	VU	10	3248	36
Allium bungei Boiss.	Amaryllidaceae	EN	4	90497	16
Allium capitellatum Boiss.	Amaryllidaceae	VU	8	89785	32
Allium cardiostemon Fisch. & C.A. Mey.	Amaryllidaceae	VU	10	16478	40
Allium caspium (Pall.) M. Bieb.	Amaryllidaceae	LC or NT	41	288347	160
Allium cathodicarpum Wendelbo	Amaryllidaceae	LC or NT	29	133264	112
Allium chloroneurum Boiss.	Amaryllidaceae	CR	1	0	4
Allium chlorotepalum R.M. Fritsch & M. Jaeger	Amaryllidaceae	EN	3	511	12
Allium chrysantherum Boiss. & Reut.	Amaryllidaceae	LC or NT	15	106180	60
Allium clivorum R.M Fritsch	Amaryllidaceae	CR	1	0	4

Allium cristophii Trautv.	Amaryllidaceae	LC or NT	24	70465	92
Allium derderianum Regel	Amaryllidaceae	LC or NT	66	19298	240
Allium dolichovaginatum R.M Fritsch	Amaryllidaceae	CR	1	0	4
Allium egorovae M.V. Agab. & Ogan.	Amaryllidaceae	LC or NT	16	24504	56
Allium elburzense Wendelbo	Amaryllidaceae	LC or NT	49	33244	172
Allium ellisii Hook.f.	Amaryllidaceae	VU	13	5063	52
Allium esfahanicum R.M. Fritsch	Amaryllidaceae	VU	11	88609	44
Allium esfandiarii Matin	Amaryllidaceae	CR	1	0	4
Allium fedtschenkoi Nábelek	Amaryllidaceae	EN	6	143867	24
Allium grande Lipsky	Amaryllidaceae	VU	11	25369	40
Allium graveolens (R.M. Fritsch) R.M. Fritsch	Amaryllidaceae	LC or NT	32	110275	124
Allium haemanthoides Boiss. & Reut. ex Regel	Amaryllidaceae	LC or NT	14	59148	56
Allium hamedanense R.M. Fritsch	Amaryllidaceae	CR	1	0	4
Allium helicophyllum Vved.	Amaryllidaceae	VU	11	52323	36
Allium hooshidaryae Mashayekhi	Amaryllidaceae	EN	3	232	12
Allium iranshahrii R.M. Fritsch	Amaryllidaceae	EN	5	1075	20
Allium jaegeri R. M. Fritsch	Amaryllidaceae	CR	1	0	4
Allium jesdianum Boiss. & Buhse	Amaryllidaceae	EN	7	43265	28
Allium joharchii F.O. Khass. et Memariani	Amaryllidaceae	CR	1	0	4
Allium kazerouni Parsa	Amaryllidaceae	LC or NT	20	36895	80
Allium keusgenii R.M. Fritsch	Amaryllidaceae	EN	5	2058	20
Allium kirindicum Bornm.	Amaryllidaceae	CR	1	0	4
Allium koelzii (Wendelbo) K. Perss. & Wendelbo	Amaryllidaceae	LC or NT	37	289666	148
Allium kotschyi Boiss.	Amaryllidaceae	EN	2	0	8
Allium kuhrangense Akhavan	Amaryllidaceae	CR	1	0	4
Allium kuhsorkhense R.M. Fritsch & Joharchi	Amaryllidaceae	LC or NT	41	39300	160
Allium kurdistanicum Maroofi & R.M. Fritsch	Amaryllidaceae	EN	4	607	12
Allium lalesaricum Freyn & Bornm.	Amaryllidaceae	EN	3	17902	12
Allium latifolium Jaub. & Spach	Amaryllidaceae	VU	8	44476	32
Allium longipapillatum R.M. Fritsch & Matin	Amaryllidaceae	EN	2	0	8

Allium longivaginatum Wendelbo	Amaryllidaceae	VU	7	8041	24
Allium mahneshanense Razyfard	Amaryllidaceae	EN	4	916	16
Allium materculae Bordz.	Amaryllidaceae	LC or NT	17	33512	68
Allium matiniae N.Friesen & M.Abbasi	Amaryllidaceae	CR	1	0	4
Allium minutiflorum Regel	Amaryllidaceae	VU	10	49263	40
Allium moderense R.M. Fritsch	Amaryllidaceae	EN	2	0	8
Allium monophyllum Vved.	Amaryllidaceae	VU	8	9786	32
Allium montelburzense R.M. Fritsch	Amaryllidaceae	EN	4	93	12
Allium mozaffarianii Maroofi & R.M. Fritsch	Amaryllidaceae	CR	2	0	4
Allium najafdaricum R. M. Fritsch	Amaryllidaceae	CR	1	0	4
Allium orientoiranicum Neshati	Amaryllidaceae	LC or NT	12	34708	44
Allium parhamii Memariani	Amaryllidaceae	CR	1	0	4
Allium pseudobodeanum R.M. Fritsch & Matin	Amaryllidaceae	VU	8	9523	32
Allium pseudohollandicum R.M. Fritsch	Amaryllidaceae	VU	7	52253	28
Allium regelii Trautv.	Amaryllidaceae	VU	11	12801	44
Allium remediorum (R.M. Fritsch) R.M. Fritsch	Amaryllidaceae	LC or NT	19	61388	72
Allium sabalense R.M. Fritsch	Amaryllidaceae	EN	5	3253	20
Allium sahandicum R.M. Fritsch	Amaryllidaceae	LC or NT	12	12326	48
Allium sanandajense Maroofi & R.M. Fritsch	Amaryllidaceae	CR	1	0	4
Allium saralicum R.M. Fritsch	Amaryllidaceae	LC or NT	22	113416	88
Allium schisticola R.M.Fritsch, Moazzeni & Dolatyari	Amaryllidaceae	CR	1	0	4
Allium scotostemon Wendelbo	Amaryllidaceae	LC or NT	37	28220	144
Allium shatakiense Rech.f.	Amaryllidaceae	CR	1	0	4
Allium shelkovnikovii Grossh.	Amaryllidaceae	LC or NT	14	96034	56
Allium straussii Bornm.	Amaryllidaceae	EN	6	2737	24
Allium subakaka Razyfard & Zarre	Amaryllidaceae	LC or NT	14	31156	56
Allium subniveale Vendelbo	Amaryllidaceae	EN	3	25933	12
Allium subnotabile Wendelbo	Amaryllidaceae	CR	1	0	4
Allium tuchalense Khassanov &Noroozi	Amaryllidaceae	EN	10	92	24
Allium ubipetrense R.M. Fritsch	Amaryllidaceae	LC or NT	55	143492	212

Allium vescum Wendelbo	Amaryllidaceae	CR	1	0	4
Allium wendelboi Matin	Amaryllidaceae	CR	1	0	4
Allium zagricum R.M. Fritsch	Amaryllidaceae	LC or NT	11	16714	44
Allochrusa lutea Falat. & Mahmoodi	Caryophyllaceae	CR	1	0	4
Allochrusa persica (Boiss.) Boiss.	Caryophyllaceae	EN	5	2346	20
Alnus djavanshirii Zare	Betulaceae	CR	1	0	4
Alnus dolichocarpa Zare	Betulaceae	EN	4	17	12
Alnus hyrcana Sharifnia & Gholamiterojeni	Betulaceae	CR	1	0	4
Alnus longiflorescentia Sharifnia & Gholamiterojeni	Betulaceae	CR	1	0	4
Alococarpum erianthum (DC.) H. Riedl	Apiaceae	LC or NT	12	14381	48
Alrawia bellii (Baker) K. Persson & Wendelbo	Asparagaceae	LC or NT	13	106337	52
Alyssum bracteatum Boiss. & Buhse	Brassicaceae	LC or NT	13	125049	52
Alyssum hezarmasjedense Kavousi & Nazary	Brassicaceae	CR	1	0	4
Alyssum lanigerum DC.	Brassicaceae	LC or NT	32	543234	128
Alyssum mazandaranicum Mirzadeh & Assadi	Brassicaceae	EN	3	7187	12
Alyssum mozaffarianii Kavousi	Brassicaceae	EN	5	77	16
Alyssum muelleri Boiss. & Buhse	Brassicaceae	VU	9	412513	36
Alyssum persicum Boiss.	Brassicaceae	EN	7	110700	24
Alyssum polycladum Rech.f.	Brassicaceae	VU	11	242271	40
Alyssum stipitatum Kavousi & Dudley	Brassicaceae	CR	1	0	4
Alyssum turgidum Dudley	Brassicaceae	VU	8	18313	32
Amberboa lippii (L.) DC.	Asteraceae	CR	1	0	4
Amberboa maroofii Negaresh	Asteraceae	CR	1	0	4
Amberboa zanjanica Ranjbar & Negaresh	Asteraceae	CR	1	0	4
Amygdalus eburnea Spach	Rosaceae	LC or NT	64	895350	256
Amygdalus elaeagnifolia Spach	Rosaceae	LC or NT	66	230775	256
Amygdalus ghahremanii Maroofi, Attar & Vafadar	Rosaceae	CR	1	0	4
Amygdalus glauca Browicz	Rosaceae	EN	3	8	8
Amygdalus haussknechtii (C.K.Schneider.) Bornm.	Rosaceae	LC or NT	36	139659	140
Amygdalus kurdistanica Attar	Rosaceae	CR	1	0	4

Amygdalus kurdistanica Attar, Maroofi & vafadar	Rosaceae	CR	1	0	4
Amygdalus orazii Maroofi	Rosaceae	CR	1	0	4
Amygdalus paboti Browicz	Rosaceae	EN	3	25	12
Amygdalus reticulata Runemark ex Khatamsaz	Rosaceae	CR	2	0	4
Amygdalus wendelboi Freitag	Rosaceae	EN	5	2125	20
Anabasis calcarea (Charif & Aellen) Bokhari & Wendelbo	Amaranthaceae	LC or NT	27	338502	100
Anabasis firouzii Akhani	Amaranthaceae	CR	1	0	4
Anabasis haussknechtii Bunge ex Boiss.	Amaranthaceae	LC or NT	12	298957	48
Anchonium elichrysifolium (DC.) Boiss.	Brassicaceae	LC or NT	29	435077	112
Angelica urumiensis Mozaff	Apiaceae	CR	1	0	4
Angelica urumiensis Mozaff.	Apiaceae	CR	1	0	4
Anthemis austroiranica Rech.f. Aell. & Esfand.	Asteraceae	LC or NT	41	622274	164
Anthemis brachystephana Bornm. & Gauba	Asteraceae	LC or NT	18	33318	68
Anthemis bushehrica Iranshahr	Asteraceae	EN	3	296	12
Anthemis fungosa Boiss. & Hausskn.	Asteraceae	CR	1	0	4
Anthemis gayana Boiss.	Asteraceae	LC or NT	25	156037	96
Anthemis gilanica Bornm. & Gauba	Asteraceae	LC or NT	20	58105	76
Anthemis gillettii Iranshahr	Asteraceae	EN	4	1505	16
Anthemis gracilis Iranshahr	Asteraceae	CR	1	0	4
Anthemis hemistephana Boiss.	Asteraceae	EN	2	0	8
Anthemis lorestanica Iranshahr	Asteraceae	VU	10	55656	40
Anthemis mazandaranica Iranshahr	Asteraceae	EN	4	70208	16
Anthemis mirheydari Iranshahr	Asteraceae	EN	4	2845	16
Anthemis moghanica Iranshahr	Asteraceae	VU	10	33396	40
Anthemis persica Boiss.	Asteraceae	LC or NT	25	116996	96
Anthochlamys multinervis Rech.f.	Amaranthaceae	LC or NT	29	149144	112
Aphanopleura breviseta (Boiss.) Heywood & Jruy	Apiaceae	LC or NT	26	107918	104
Arabis ottonis-schulzii Bornm. & Gauba	Brassicaceae	EN	4	176	16
Arabis rimarum Rech.f.	Brassicaceae	EN	5	11022	20
Arenaria assadii F. Fadaie	Caryophyllaceae	CR	1	0	4

Arenaria bulica Stapf. Ex Williams	Caryophyllaceae	EN	2	0	8
Arenaria kandavanensis F. Fadaie	Caryophyllaceae	CR	1	0	4
Arenaria longibracteata F. Fadaie	Caryophyllaceae	CR	1	0	4
Arenaria minutissima Rech.f. & Esfand.	Caryophyllaceae	EN	2	0	8
Arenaria persica Boiss.	Caryophyllaceae	LC or NT	13	116727	52
Arenaria polycnemifolia Boiss.	Caryophyllaceae	VU	7	30525	28
Arenaria semiromica F. Fadaie	Caryophyllaceae	CR	1	0	4
Arenaria szowitsii Boiss.	Caryophyllaceae	VU	8	18244	32
Arenaria tetrasticha Boiss.	Caryophyllaceae	EN	4	73167	16
Arenaria yazdiana Assadi	Caryophyllaceae	EN	2	0	8
Arenaria zargariana Parsa	Caryophyllaceae	VU	9	27734	36
Argyrolobium trigonelloides Jaub. & Spach	Fabaceae	LC or NT	20	479360	76
Aristolochia hyrcana Davis & M.S.Khan	Aristolochiaceae	EN	6	346	24
Aristolochia olivieri Collengo	Aristolochiaceae	LC or NT	43	224052	168
Arnebia violascens H.Riedl	Boraginaceae	CR	1	0	4
Artemisia kermanensis Podl.	Asteraceae	VU	9	90635	36
Artemisia melanolepis Boiss.	Asteraceae	EN	8	50771	20
Arum giganteum Ghehreman	Araceae	VU	6	88024	24
Asparagus bojnurdensis Hamdi & Assadi	Liliaceae	EN	2	0	8
Asparagus khorasanensis Hamdi & Assadi	Asparagaceae	EN	2	0	8
Asparagus touranensis Hamdi & Assadi	Asparagaceae	EN	5	488997	20
Asperula brachyantha Boiss.	Rubiaceae	LC or NT	13	291830	52
Asperula fragillima Boiss. & Hausskn. ex Boiss.	Rubiaceae	VU	12	32714	44
Asperula gorganica Schonb.-Tem. & Ehrend.	Rubiaceae	EN	6	938	24
Asperula mazanderanica Ehrend.	Rubiaceae	EN	6	11398	24
Asperula microphylla Boiss.	Rubiaceae	VU	13	4081	52
Asperula rechingeri Ehrend. & Schonb.- Tem	Rubiaceae	LC or NT	18	37256	68
Asperula rezaiyensis Schonb.-Tem.	Rubiaceae	CR	2	0	4
Asperula seticornis Boiss.	Rubiaceae	EN	3	19	12
Asperula sherardioides (Boiss.) Jaub. & Spach	Rubiaceae	CR	1	0	4

Aster bachtiaricus Mozaffarian	Asteraceae	CR	1	0	4
Astragalus abadehensis Maassoumi & Podlech	Fabaceae	CR	1	0	4
Astragalus abharensis Maassoumi & Podlech	Fabaceae	EN	3	23	12
Astragalus abnormalis Rech.f.	Fabaceae	VU	9	81784	36
Astragalus absconditus Zarre & Podlech	Fabaceae	CR	1	0	4
Astragalus absentivus Maassoumi	Fabaceae	CR	1	0	4
Astragalus acutifolius Bunge	Fabaceae	CR	2	0	4
Astragalus adulterinus Podl.	Fabaceae	EN	3	22539	12
Astragalus aestimabilis Podlech	Fabaceae	CR	1	0	4
Astragalus aestivorum Podlech	Fabaceae	EN	2	0	8
Astragalus affinis Podlech & Zarre	Fabaceae	CR	1	0	4
Astragalus agassii Manden.	Fabaceae	DD	NA	NA	NA
Astragalus ahangarensis Zarre & Podlech	Fabaceae	EN	3	40112	12
Astragalus aharicus Maassoumi & Podlech	Fabaceae	VU	13	5074	40
Astragalus ahmad-parsae Maassoumi	Fabaceae	CR	1	0	4
Astragalus aiwadzhi B.Fedtsch.	Fabaceae	DD	NA	NA	NA
Astragalus akhanii Podlech	Fabaceae	CR	1	0	4
Astragalus akhundzadahensis Podlech & Zarre	Fabaceae	DD	NA	NA	NA
Astragalus alamkuhensis Maassoumi	Fabaceae	DD	NA	NA	NA
Astragalus alamouticus Maassoumi	Fabaceae	EN	2	0	8
Astragalus alavaanus Podlech	Fabaceae	EN	2	0	8
Astragalus albispinus	Fabaceae	LC or NT	17	230015	68
Astragalus alienus Podlech	Fabaceae	EN	4	3600	16
Astragalus aliomranii Maassoumi	Fabaceae	CR	1	0	4
Astragalus altimontanus Podlech & Maassoumi	Fabaceae	CR	1	0	4
Astragalus altiusculus Maassoumi & Ghahremani	Fabaceae	EN	4	13	8
Astragalus alyssiformis Maassoumi	Fabaceae	DD	NA	NA	NA
Astragalus ammodendroides Podlech & Zarre	Fabaceae	CR	2	0	4
Astragalus anacamptus Bunge	Fabaceae	EN	3	1033	12
Astragalus andabaddensis Maassoumi, Bagheri & F.Ghahrem.	Fabaceae	CR	1	0	4

Astragalus andabilensis Ranjbar & Mahmoudian	Fabaceae	CR	1	0	4
Astragalus anguranensis Podlech & Maassoumi	Fabaceae	VU	6	32568	24
Astragalus angustistipulatus Podlech	Fabaceae	EN	4	4453	16
Astragalus anodiophilus Zarre & Podlech	Fabaceae	CR	1	0	4
Astragalus anserinaefolius Boiss.	Fabaceae	LC or NT	44	300627	172
Astragalus arasbaranensis Maassoumi & Ranjbar	Fabaceae	CR	1	0	4
Astragalus archibaldii Podl.	Fabaceae	EN	5	132	16
Astragalus ardahalicus Parsa	Fabaceae	EN	9	5925	36
Astragalus argyrostachys Boiss.	Fabaceae	VU	10	3576	36
Astragalus ashtianensis Podlech & Maassoumi	Fabaceae	CR	1	0	4
Astragalus askius Bunge	Fabaceae	LC or NT	40	15949	152
Astragalus aspadanus Bunge	Fabaceae	LC or NT	15	57231	60
Astragalus aspreticola Podlech	Fabaceae	EN	2	0	8
Astragalus assadii Maassoumi & Podl.	Fabaceae	EN	4	130	16
Astragalus atricapillus Bornm.	Fabaceae	VU	10	19885	36
Astragalus atrokurdicus Maassoumi, F. Ghahrem., Bagheri & Podlech	Fabaceae	CR	1	0	4
Astragalus attarae Podlech	Fabaceae	EN	3	1516	12
Astragalus austrokhorasanicus Podlech	Fabaceae	CR	1	0	4
Astragalus austromahneshanensis Ghahrem.	Fabaceae	CR	1	0	4
Astragalus austrotaromensis Maassoumi, F. Ghahrem., Bagheri & Podlech	Fabaceae	CR	1	0	4
Astragalus avajensis Podlech	Fabaceae	CR	2	0	4
Astragalus avicennicus Parsa	Fabaceae	LC or NT	36	81487	136
Astragalus ayatollahii Nasseh, Joharchi & F.Ghahrem.	Fabaceae	DD	NA	NA	NA
Astragalus azizii Maassoumi	Fabaceae	CR	1	0	4
Astragalus aznabjurticus Grossh.	Fabaceae	LC or NT	19	86091	76
Astragalus aznaicus Podlech & Maassoumi	Fabaceae	CR	1	0	4
Astragalus baba-alliar Parsa	Fabaceae	VU	14	78980	56
Astragalus babakhanloui Maassoumi & Podl.	Fabaceae	LC or NT	20	66778	80
Astragalus badelehensis Maassoumi & Taheri	Fabaceae	DD	NA	NA	NA

Astragalus badelehensis Maassoumi & Taheri	Fabaceae	DD	NA	NA	NA
Astragalus baftensis Ranjbar & Maassoumi	Fabaceae	EN	2	0	8
Astragalus baharensis Ghahremaninejad	Fabaceae	CR	1	0	4
Astragalus bajgiranensis Podlech	Fabaceae	EN	2	0	8
Astragalus baraftabensis Maassoumi & Podlech	Fabaceae	EN	5	1213	20
Astragalus barnasariformis Maassoumi	Fabaceae	CR	1	0	4
Astragalus bashmaghensis Maassoumi & Podlech	Fabaceae	EN	4	1875	16
Astragalus bashmensis Maassoumi	Fabaceae	CR	1	0	4
Astragalus basilicus Maassoumi & Podl.	Fabaceae	VU	8	12298	32
Astragalus bavanatensis Zarre & Podlech	Fabaceae	EN	2	0	8
Astragalus bavanaticus Maassoumi	Fabaceae	CR	1	0	4
Astragalus bazarganii Podlech & Zarre	Fabaceae	VU	8	56264	32
Astragalus bazmanicus Podlech	Fabaceae	CR	2	0	4
Astragalus beckii Bornm.	Fabaceae	LC or NT	26	116432	100
Astragalus bejourensis Podlech & Maassoumi	Fabaceae	EN	5	2804	20
Astragalus belgheisicoides Podlech & Maassoumi	Fabaceae	EN	2	0	8
Astragalus belgheisicus Maassoumi	Fabaceae	LC or NT	12	91068	48
Astragalus biarjmandicus Podlech & Zarre	Fabaceae	DD	NA	NA	NA
Astragalus bifoliolatus Sirj. & Rech. f.	Fabaceae	DD	NA	NA	NA
Astragalus bijarensis Podlech & Sytin	Fabaceae	EN	4	1793	16
Astragalus birangae Maassoumi	Fabaceae	EN	2	0	8
Astragalus biserrula Bunge	Fabaceae	LC or NT	15	488249	60
Astragalus bodeanus Fisch.	Fabaceae	LC or NT	13	61963	52
Astragalus bojnurdensis Podlech	Fabaceae	EN	2	0	8
Astragalus bordschensis Bornm.	Fabaceae	EN	5	410	20
Astragalus borujenensis Ranjbar & Maassoumi	Fabaceae	EN	2	0	8
Astragalus botryophorus Maassoumi & Podl.	Fabaceae	EN	5	3048	20
Astragalus bounophilus Boiss. & Hohen.	Fabaceae	LC or NT	20	85643	80
Astragalus bozghoushensis Maassoumi, Mozaff. & Ramezani	Fabaceae	CR	1	0	4
Astragalus brachyodontus Boiss.	Fabaceae	LC or NT	154	452304	604

Astragalus bradosticus Maassoumi & Podlech	Fabaceae	EN	2	0	8
Astragalus brevicalycinus Maassoumi	Fabaceae	CR	1	0	4
Astragalus brevifructus Podlech in Podlech & Zarre	Fabaceae	CR	1	0	4
Astragalus brevipedunculatus Ranjbar	Fabaceae	DD	NA	NA	NA
Astragalus brevirhachis Tietz & Zarre	Fabaceae	DD	NA	NA	NA
Astragalus brevitomentosus Podlech	Fabaceae	EN	6	2592	24
Astragalus brunsianus Bornm.	Fabaceae	EN	3	434	12
Astragalus bukanensis Maassoumi & Podlech	Fabaceae	CR	1	0	4
Astragalus caespititius Podlech	Fabaceae	CR	1	0	4
Astragalus callainus Podlech	Fabaceae	VU	7	18761	28
Astragalus calliphysa Bunge	Fabaceae	LC or NT	62	301562	244
Astragalus campylanthoides Bornm.	Fabaceae	EN	3	3834	12
Astragalus campylanthus Boiss.	Fabaceae	LC or NT	32	219146	124
Astragalus canosus Maassoumi	Fabaceae	EN	4	354	12
Astragalus capax Maassoumi	Fabaceae	LC or NT	16	38709	64
Astragalus capito Boiss.	Fabaceae	EN	4	1311	16
Astragalus carmanicus Bornm.	Fabaceae	VU	7	9647	28
Astragalus catacamptus Bunge	Fabaceae	LC or NT	21	294574	84
Astragalus cemerinus Beck	Fabaceae	LC or NT	19	47915	76
Astragalus cephalanthus DC.	Fabaceae	LC or NT	76	258766	300
Astragalus chaetopodus Bunge	Fabaceae	VU	7	6102	28
Astragalus chahartaghensis Maassoumi & Podl.	Fabaceae	VU	7	16023	28
Astragalus chalaranthus Boiss. & Hausskn. in Boiss.	Fabaceae	LC or NT	23	72004	92
Astragalus chamanbidensis Maassoumi & Mozaff.	Fabaceae	CR	1	0	4
Astragalus chartostegius Boiss. & Hausskn.	Fabaceae	VU	12	12904	44
Astragalus chehregani Zarre & Podl.	Fabaceae	EN	4	219	16
Astragalus chichesticus Podlech & Maassoumi	Fabaceae	CR	1	0	4
Astragalus chrysanthus Boiss. & Hohen	Fabaceae	VU	12	11443	36
Astragalus chrysotrichus Boiss.	Fabaceae	LC or NT	31	181002	120
Astragalus ciceropsis Hamzeh'ee & Maassoumi	Fabaceae	CR	1	0	4

Astragalus circumlacustris Podlech & Sytin	Fabaceae	EN	2	0	8
Astragalus clivicola Podlech & Maassoumi	Fabaceae	EN	3	172	12
Astragalus coluteopsis Parsa	Fabaceae	VU	6	32017	24
Astragalus confertiformis Širj. & Rech.f.	Fabaceae	EN	5	3821	20
Astragalus confusus Bunge.	Fabaceae	LC or NT	19	16426	72
Astragalus controversus Maassoumi & Podl.	Fabaceae	EN	3	159	12
Astragalus craccinopsis Maassoumi	Fabaceae	CR	1	0	4
Astragalus crispus F. Ghahremaninejad	Fabaceae	CR	1	0	4
Astragalus culminatus Maassoumi	Fabaceae	CR	2	0	4
Astragalus curviflorus Boiss.	Fabaceae	LC or NT	24	127694	96
Astragalus cyclophyllon Beck	Fabaceae	LC or NT	54	169460	208
Astragalus cystosus Zarre & Podlech	Fabaceae	CR	2	0	4
Astragalus daenensis Boiss.	Fabaceae	LC or NT	18	75763	72
Astragalus daghdaghabadensis Maassoumi	Fabaceae	EN	5	30319	20
Astragalus damghanensis Podlech	Fabaceae	CR	1	0	4
Astragalus darlingtonii Podl.	Fabaceae	LC or NT	14	76304	52
Astragalus darrehbidensis Podlech & Zarre	Fabaceae	CR	1	0	4
Astragalus dejectus Maassoumi, F. Ghahrem. & Bagheri	Fabaceae	CR	1	0	4
Astragalus delutulus Maassoumi	Fabaceae	VU	7	24284	28
Astragalus demavendicola Bornm. & Gauba	Fabaceae	LC or NT	21	77812	84
Astragalus demavendicus Boiss. & Buhse	Fabaceae	LC or NT	21	32341	84
Astragalus demonstratus Maassoumi	Fabaceae	CR	1	0	4
Astragalus dengolanensis Podlech	Fabaceae	CR	1	0	4
Astragalus denticulatus Podl.	Fabaceae	CR	2	0	4
Astragalus dianat-nejadii F. Ghahremani	Fabaceae	EN	4	870	16
Astragalus dictyolobus Bunge	Fabaceae	LC or NT	23	73299	88
Astragalus dinawarii Bidarlord & F.Ghahrem.	Fabaceae	CR	1	0	4
Astragalus distans Fisch.	Fabaceae	EN	3	1144	12
Astragalus divandarrehensis Podlech	Fabaceae	LC or NT	17	7312	68
Astragalus diversipilosus Podlech	Fabaceae	EN	2	0	8

Astragalus diversus Podlech & Maassoumi	Fabaceae	EN	2	0	8
Astragalus doghrunensis Maassoumi & Podlech	Fabaceae	EN	4	3982	16
Astragalus dopolanicus Podlech	Fabaceae	EN	4	4845	16
Astragalus dorudensis Zarre & Podlech	Fabaceae	EN	2	0	8
Astragalus doshman-ziariensis Maassoumi & Podlech	Fabaceae	EN	3	11774	12
Astragalus dschuparensis Freyn & Bornm.	Fabaceae	LC or NT	15	110939	56
Astragalus durandianus Aitch. & Baker	Fabaceae	LC or NT	17	99659	68
Astragalus dysbatophilus Zarre & Podlech	Fabaceae	CR	1	0	4
Astragalus ebenoides Boiss.	Fabaceae	LC or NT	42	161439	168
Astragalus ebrahimabadensis Zarre & Podlech	Fabaceae	CR	1	0	4
Astragalus eburneus Bornm. & Gauba	Fabaceae	LC or NT	15	164559	60
Astragalus ecbatanus Bunge	Fabaceae	LC or NT	14	33948	52
Astragalus echidna Bunge	Fabaceae	LC or NT	16	54673	64
Astragalus edmondsonii Podlech	Fabaceae	EN	3	38	12
Astragalus elezgensis Maassoumi & Kazempour	Fabaceae	EN	4	22520	16
Astragalus elwendicus Bornm.	Fabaceae	VU	8	22095	28
Astragalus erinifolius Pau	Fabaceae	EN	2	0	8
Astragalus eriopodus Boiss.	Fabaceae	LC or NT	41	142649	160
Astragalus eriostomus Bornm.	Fabaceae	LC or NT	11	27951	44
Astragalus erubescens Podl.	Fabaceae	EN	4	10710	16
Astragalus erwinii-gaubae Sirj. & Rech.f.	Fabaceae	VU	8	9226	32
Astragalus erythrolepis Boiss.	Fabaceae	EN	2	0	8
Astragalus esferayenicus Podl. & Maassoumi	Fabaceae	EN	5	8557	20
Astragalus estahbanensis Maassoumi & Podlech	Fabaceae	CR	1	0	4
Astragalus eusarathron I.Deml & Podlech	Fabaceae	CR	2	0	4
Astragalus evanensis Maassoumi & Podl.	Fabaceae	EN	4	46	8
Astragalus expetitus Maassoumi	Fabaceae	DD	NA	NA	NA
Astragalus exspectatus Maassoumi	Fabaceae	EN	3	8479	12
Astragalus facetus Maassoumi & Podl.	Fabaceae	CR	1	0	4
Astragalus fagh-soleimanensis Maassoumi & Podlech	Fabaceae	VU	9	7777	36

Astragalus farsicus Sirj & Rech.f.	Fabaceae	VU	8	67060	32
Astragalus fasciculifolius Boiss.	Fabaceae	LC or NT	21	95438	80
Astragalus fausicola Podlech ex Bagheri	Fabaceae	CR	1	0	4
Astragalus ferruminatus Maassoumi	Fabaceae	EN	5	6963	20
Astragalus filifoliolatus Maassoumi	Fabaceae	EN	2	0	8
Astragalus firuzkuhensis Podl.	Fabaceae	CR	1	0	4
Astragalus fischeri Fisch.	Fabaceae	LC or NT	46	88453	184
Astragalus flexilipes Bornm.	Fabaceae	EN	5	7865	20
Astragalus floccosus Boiss	Fabaceae	LC or NT	94	567564	360
Astragalus foliosus Podlech	Fabaceae	CR	1	0	4
Astragalus fortuitus Maassoumi	Fabaceae	CR	1	0	4
Astragalus fragiferus Bunge	Fabaceae	LC or NT	15	96082	60
Astragalus fridae Rech.f.	Fabaceae	EN	13	9594	44
Astragalus fuliginosus Beck	Fabaceae	VU	10	46764	40
Astragalus gagnieui Maassoumi & Podl.	Fabaceae	EN	3	4413	12
Astragalus gamasiabensis Maassoumi	Fabaceae	EN	2	0	4
Astragalus gandomanicus Podlech	Fabaceae	CR	1	0	4
Astragalus garmashubensis Maassoumi	Fabaceae	CR	1	0	4
Astragalus gaubae Bornm.	Fabaceae	EN	3	5842	12
Astragalus ghahremanii Maassoumi & Podl.	Fabaceae	VU	6	51852	24
Astragalus ghamishluensis Dastpak	Fabaceae	CR	1	0	4
Astragalus ghanbarianii Maassoumi, Zarre & Podlech	Fabaceae	CR	1	0	4
Astragalus ghashghaicus Tietz & Zarre	Fabaceae	EN	5	8790	20
Astragalus ghouchanensis Souzani	Fabaceae	CR	1	0	4
Astragalus gifanicus Maassoumi & Podlech	Fabaceae	EN	3	258	12
Astragalus gigantifoliolatus Maassoumi & Maroofi	Fabaceae	CR	1	0	4
Astragalus gigantirostratus Maassoumi et al.	Fabaceae	EN	4	620	16
Astragalus gilvanensis Ranjbar & Nouri	Fabaceae	CR	1	0	4
Astragalus glaucacanthus Fisch.	Fabaceae	LC or NT	79	566453	316
Astragalus glaucops Bornm.	Fabaceae	VU	10	6537	32

Astragalus glaucopsiformis Maassoumi	Fabaceae	DD	NA	NA	NA
Astragalus glochidiatus Maassoumi	Fabaceae	CR	1	0	4
Astragalus glumaceus Boiss.	Fabaceae	LC or NT	37	182533	148
Astragalus gonabadensis Nasseh, Joharchi & F.Ghahrem.	Fabaceae	DD	NA	NA	NA
Astragalus griseus Boiss.	Fabaceae	LC or NT	31	532708	120
Astragalus gueldenstaedtiae Bunge	Fabaceae	EN	4	1476	16
Astragalus gulul-saranii Podlech	Fabaceae	EN	3	18111	12
Astragalus gypsaceus Beck	Fabaceae	LC or NT	12	100635	44
Astragalus gypsicola Maassoumi & Mozaffarian	Fabaceae	CR	1	0	4
Astragalus hajiabadensis Podlech & Maassoumi	Fabaceae	CR	1	0	4
Astragalus hajijafanensis Maassoumi	Fabaceae	CR	1	0	4
Astragalus hamadanus Boiss.	Fabaceae	LC or NT	13	167517	52
Astragalus hamadryadis Podlech	Fabaceae	CR	1	0	4
Astragalus harazensis Zarre & Podlech	Fabaceae	EN	3	291	12
Astragalus hecatae Zarre & Podlech	Fabaceae	CR	1	0	4
Astragalus heinzianus Maassoumi & Mazaff.	Fabaceae	CR	1	0	4
Astragalus hekmat-Safaviae Ghahrem.	Fabaceae	EN	3	136	12
Astragalus herbertii Maassoumi	Fabaceae	EN	3	480	12
Astragalus hermannii Freitag & Podl.	Fabaceae	EN	3	203	12
Astragalus heterodoxus Bunge.	Fabaceae	VU	11	23830	44
Astragalus heterophyllus Podlech	Fabaceae	EN	7	7999	28
Astragalus hirticalyx Bunge	Fabaceae	DD	NA	NA	NA
Astragalus hirtus Bunge	Fabaceae	EN	3	854	12
Astragalus holopsilus Bunge	Fabaceae	LC or NT	19	51839	72
Astragalus holosemius Bunge	Fabaceae	VU	10	21957	40
Astragalus homandicus Maassoumi & Podlech	Fabaceae	EN	2	0	8
Astragalus horridus Boiss.	Fabaceae	LC or NT	14	100624	52
Astragalus hotkanensis Maassoumi & Mirtadzadini	Fabaceae	EN	2	0	8
Astragalus huthianus Freyn & Bornm.	Fabaceae	VU	8	7826	32
Astragalus hymenocalyx Boiss.	Fabaceae	VU	12	15023	40

Astragalus hymenostegis Fisch. & C.A. Mey.	Fabaceae	VU	9	14466	36
Astragalus hypsogeton Bonge	Fabaceae	EN	5	9941	20
Astragalus ibicinus Boiss. & Haussk. in Boiss.	Fabaceae	LC or NT	17	47723	64
Astragalus ignotus Podlech	Fabaceae	CR	1	0	4
Astragalus ilachchiensis Ranjbar & Zarin	Fabaceae	CR	1	0	4
Astragalus imbecillus Maassoumi & Podl.	Fabaceae	CR	1	0	4
Astragalus impexus Podl.	Fabaceae	EN	6	56075	24
Astragalus inchebroonensis Maassoumi	Fabaceae	CR	1	0	4
Astragalus indistinctus Podl. & Maassoumi	Fabaceae	VU	12	61616	44
Astragalus indomitus Maassoumi	Fabaceae	DD	NA	NA	NA
Astragalus inexpectatus Maassoumi & Podl.	Fabaceae	CR	1	0	4
Astragalus inexspectatus Maassoumi & Podlech	Fabaceae	EN	5	6128	20
Astragalus innotabilis Podlech	Fabaceae	CR	1	0	4
Astragalus inquilinus Maassoumi	Fabaceae	EN	2	0	8
Astragalus insularis Maassoumi & Podlech	Fabaceae	CR	1	0	4
Astragalus iodotropis Boiss. & Hohen.	Fabaceae	LC or NT	22	10917	68
Astragalus iranicus Bunge	Fabaceae	LC or NT	173	715527	676
Astragalus iranshahrii Maassoumi & Podl.	Fabaceae	VU	11	34621	44
Astragalus ischredensis Bunge	Fabaceae	LC or NT	27	159195	108
Astragalus issatissensis Maassoumi & Mahmoodi	Fabaceae	CR	2	0	4
Astragalus jacobsii Podlech	Fabaceae	LC or NT	11	59266	44
Astragalus jamzadiae Maassoumi	Fabaceae	CR	1	0	4
Astragalus jaskensis Maassoumi	Fabaceae	CR	1	0	4
Astragalus jesdianus Boiss. & Buhse	Fabaceae	LC or NT	28	151015	112
Astragalus jessenii Bunge	Fabaceae	LC or NT	43	33285	152
Astragalus johannis Boiss.	Fabaceae	LC or NT	39	40871	148
Astragalus joharchii Ghahremani nejad & J. F. Gaskin	Fabaceae	EN	4	28655	16
Astragalus juladakensis Maassoumi	Fabaceae	CR	1	0	4
Astragalus kabutarlanensis Dehshiri & Maassoumi	Fabaceae	CR	1	0	4
Astragalus kadschoroides Ranjbar	Fabaceae	DD	NA	NA	NA

Astragalus kalatehensis Maassoumi & Kazempour	Fabaceae	CR	1	0	4
Astragalus kaleibarensis Podlech	Fabaceae	EN	2	0	8
Astragalus karl-heinzii Maassoumi	Fabaceae	CR	1	0	4
Astragalus kashafensis Podl.	Fabaceae	EN	2	0	8
Astragalus kashmarensis Maassoumi & Podl.	Fabaceae	VU	6	21659	24
Astragalus kaswinensis Bornm.	Fabaceae	CR	1	0	4
Astragalus kavirensis Freitag	Fabaceae	CR	1	0	4
Astragalus kazempourii Bagheri, Maassoumi & Mahmoodi	Fabaceae	CR	1	0	4
Astragalus kelishomensis Maassoumi, Mozaff. & Moradi	Fabaceae	EN	2	0	8
Astragalus kentrophyllus Podlech	Fabaceae	LC or NT	17	128226	68
Astragalus keredjensis Podlech	Fabaceae	LC or NT	24	11458	92
Astragalus kermanschahensis Bornm.	Fabaceae	EN	3	1228	12
Astragalus khadem-kandicus Maassoumi & Podlech	Fabaceae	EN	2	0	8
Astragalus khajehensis Ghahremani	Fabaceae	CR	1	0	4
Astragalus khajiboulaghensis Maassoumi	Fabaceae	EN	2	0	8
Astragalus khaneradarensis Sirj. & Rech.f.	Fabaceae	EN	3	3655	12
Astragalus kharvanensis Ranjbar	Fabaceae	CR	1	0	4
Astragalus khatamsazae Maassoumi	Fabaceae	CR	1	0	4
Astragalus khongensis Maassoumi	Fabaceae	CR	1	0	4
Astragalus khonikensis Nasseh & Joharchi	Fabaceae	CR	1	0	4
Astragalus khoshjailensis Sirj. & Rech.f.	Fabaceae	VU	15	10940	60
Astragalus khosrowabadensis Ramjbar & Karamian	Fabaceae	CR	1	0	4
Astragalus khunsarensis Zarre & Podlech	Fabaceae	CR	1	0	4
Astragalus kiamaky-daghensis Maassoumi & Podlech	Fabaceae	CR	1	0	4
Astragalus koelzii Barneby	Fabaceae	CR	1	0	4
Astragalus kohrudicus Bunge	Fabaceae	VU	10	47476	40
Astragalus kordloricus Zarre	Fabaceae	VU	8	4224	32
Astragalus kuhidashtehensis Podlech	Fabaceae	EN	7	3548	28
Astragalus kurdaicus Saposhn. & Summ.	Fabaceae	DD	NA	NA	NA
Astragalus lacus-valashti Maassoumi	Fabaceae	EN	5	68	16

Astragalus lalesarensis Bornm.	Fabaceae	EN	3	2031	12
Astragalus lambinonii Podl.	Fabaceae	EN	4	4764	16
Astragalus laricus Boiss. & Hohen.	Fabaceae	EN	2	0	4
Astragalus laristanicus Bornm. & Gauba	Fabaceae	LC or NT	13	138989	52
Astragalus lasiocalycinus Podlech & Maassoumi	Fabaceae	EN	2	0	8
Astragalus lateritiiformis Zarre	Fabaceae	LC or NT	22	21388	80
Astragalus lateritius Boiss & Hausskn. In Boiss.	Fabaceae	VU	10	28427	36
Astragalus latianicus Maassoumi & Ranjbar	Fabaceae	CR	3	12	12
Astragalus ledinghamii Barneby	Fabaceae	LC or NT	36	136703	144
Astragalus leonardii Maassoumi	Fabaceae	EN	2	0	8
Astragalus lepidus Podl.	Fabaceae	EN	4	595	16
Astragalus leptynticus Maassoumi	Fabaceae	VU	8	1675	32
Astragalus leucocerciformis Maassoumi & Ranjbar	Fabaceae	DD	NA	NA	NA
Astragalus leucophanus Bornm.	Fabaceae	EN	3	8047	12
Astragalus leucoptilus Boiss. & Hausskn.	Fabaceae	EN	4	47	16
Astragalus lignipes Akhavan & Maassoumi	Fabaceae	CR	1	0	4
Astragalus lilacinus Boiss.	Fabaceae	LC or NT	53	167121	196
Astragalus lisaricus Maassoumi	Fabaceae	DD	NA	NA	NA
Astragalus longicuspis Bunge	Fabaceae	LC or NT	17	71444	68
Astragalus longirostratus Pau	Fabaceae	LC or NT	15	19717	60
Astragalus longistylus Bunge	Fabaceae	LC or NT	45	323759	176
Astragalus lurorum Bornm.	Fabaceae	VU	9	9460	32
Astragalus lycioides Boiss.	Fabaceae	LC or NT	22	366545	88
Astragalus maabudii Ranjbar	Fabaceae	EN	3	4873	12
Astragalus maarofii Podlech & Maassoumi	Fabaceae	CR	2	0	4
Astragalus maassoumii Podl.	Fabaceae	LC or NT	13	127881	52
Astragalus macrosemius Boiss. & Hohen.	Fabaceae	EN	14	2413	28
Astragalus magistratus Maassoumi	Fabaceae	VU	11	6002	40
Astragalus magnibracteatus Maassoumi & Maroofi	Fabaceae	EN	2	0	4
Astragalus mahneshanensis Maassoumi & Moussavi	Fabaceae	EN	2	0	8

Astragalus makuensis Maassoumi, Bagheri & Rahimin.	Fabaceae	CR	1	0	4
Astragalus malaviensis Maassoumi & Mehrnia	Fabaceae	CR	1	0	4
Astragalus managettae Sirj. & Rech.f.	Fabaceae	VU	8	8561	32
Astragalus marandicus Podlech	Fabaceae	CR	1	0	4
Astragalus margonensis Ranjbar	Fabaceae	CR	1	0	4
Astragalus markasicus Podlech & Maassoumi	Fabaceae	CR	1	0	4
Astragalus masulehensis Ranjbar & Assadi	Fabaceae	DD	NA	NA	NA
Astragalus megalocystis Bunge	Fabaceae	EN	6	2531	20
Astragalus mehranensis Maassoumi & Mozaffarian	Fabaceae	CR	1	0	4
Astragalus mehrizianus Podlech & Maassoumi	Fabaceae	CR	1	0	4
Astragalus meimandicus Maassoumi & Vakili	Fabaceae	CR	1	0	4
Astragalus melanocalyx Boiss. & Buhse	Fabaceae	EN	5	8621	20
Astragalus melanodon Boiss.	Fabaceae	VU	11	134148	40
Astragalus membranostipulus Maassoumi	Fabaceae	EN	2	0	8
Astragalus memnonius Maassoumi & Podl.	Fabaceae	EN	3	187	12
Astragalus memoriosus Pakravan	Fabaceae	CR	2	0	4
Astragalus meshkinensis Podlech	Fabaceae	CR	1	0	4
Astragalus microfoliolatus Nasseh	Fabaceae	CR	1	0	4
Astragalus microphysa Boiss.	Fabaceae	LC or NT	14	66094	56
Astragalus minuticalycinus Podlech & Zarre	Fabaceae	CR	1	0	4
Astragalus minutulus Maassoumi	Fabaceae	CR	1	0	4
Astragalus modestus Boiss. & Hohen.	Fabaceae	VU	9	5810	32
Astragalus monanthemus Boiss.	Fabaceae	EN	6	2832	24
Astragalus monozyx Bornm.	Fabaceae	CR	1	0	4
Astragalus montis-alamkuhi Maassoumi	Fabaceae	DD	NA	NA	NA
Astragalus montis-bakhtiari Maassoumi & Sardari	Fabaceae	CR	1	0	4
Astragalus montismishoudaghi Sheikh Akbari Mehr	Fabaceae	CR	1	0	4
Astragalus montis-nacarouzii Maassoumi & Maroofi	Fabaceae	EN	2	0	8
Astragalus montis-parrowii Maassoumi & Nemati	Fabaceae	EN	2	0	8
Astragalus montis-varvashti Podlech	Fabaceae	CR	1	0	4

Astragalus mostafa-assadii Bagheri, Maassoumi, F. Ghahrem. & Podlech	Fabaceae	CR	1	0	4
Astragalus mozaffarianii Maassoumi	Fabaceae	CR	1	0	4
Astragalus mucronifolius Boiss.	Fabaceae	LC or NT	63	664223	244
Astragalus murinus Boiss.	Fabaceae	LC or NT	41	82879	160
Astragalus musaianus Maassoumi & Joharchi	Fabaceae	EN	2	0	8
Astragalus mutuus Podlech	Fabaceae	CR	1	0	4
Astragalus myriacanthus Boiss.	Fabaceae	LC or NT	85	446955	332
Astragalus naftabensis Sirj. & Rech.f.	Fabaceae	EN	7	1951	24
Astragalus nahavandicus Maassoumi	Fabaceae	VU	8	16363	32
Astragalus nalbandanicus Podlech	Fabaceae	EN	2	0	8
Astragalus neo-assadabadensis Podlech	Fabaceae	CR	1	0	4
Astragalus neoassadianus Ranjbar	Fabaceae	CR	1	0	4
Astragalus neochaldoranicus Podlech & Maassoumi	Fabaceae	VU	7	2578	28
Astragalus neo-iranshahrii Maassoumi & Amini Rad	Fabaceae	CR	1	0	4
Astragalus neomaassoumianus Ranjbar	Fabaceae	CR	1	0	4
Astragalus neomobayenii Maassoumi	Fabaceae	CR	2	0	4
Astragalus neo-mozaffarianii Maassoumi	Fabaceae	CR	1	0	4
Astragalus neopodlechii Maassoumi	Fabaceae	EN	5	41167	20
Astragalus neosytinii Ranjbar	Fabaceae	CR	2	0	4
Astragalus nervifolius Maassoumi, Podlech & Zarre	Fabaceae	CR	1	0	4
Astragalus neyshaburensis Podlech	Fabaceae	EN	5	355	20
Astragalus nezva-montis Podlech & Zarre	Fabaceae	EN	2	0	8
Astragalus nowroozii Podlech & Zarre	Fabaceae	CR	1	0	4
Astragalus nubicola Podlech	Fabaceae	EN	2	0	8
Astragalus nurabadensis Maassoumi & Podl.	Fabaceae	EN	3	18	12
Astragalus nurensis Boiss. & Buhse	Fabaceae	LC or NT	36	38720	136
Astragalus ochreatus Bunge	Fabaceae	VU	13	53584	48
Astragalus ochrochlorus Boiss. & Hohen.	Fabaceae	LC or NT	32	56510	124
Astragalus olangensis Maassoumi & Joharchi	Fabaceae	CR	1	0	4
Astragalus oligoflorus Maassoumi	Fabaceae	CR	1	0	4

Astragalus orientopersicus F.Ghahrem., Joharchi, Fereid. & Hoseini	Fabaceae	CR	1	0	4
Astragalus orthocarpoides Sirj. & Rech.f.	Fabaceae	EN	2	0	8
Astragalus ovigerus Boiss.	Fabaceae	EN	5	1020	20
Astragalus ovoideus Sirj. & Rech.f.	Fabaceae	LC or NT	84	387006	332
Astragalus pakravaniae Podlech & Maassoumi	Fabaceae	EN	4	164	12
Astragalus paralurges Bunge	Fabaceae	LC or NT	12	19158	48
Astragalus paralurgiformis F. Ghahrem., Maassoumi & Bagheri	Fabaceae	CR	1	0	4
Astragalus parvarensis Podlech & Sytin	Fabaceae	EN	3	111	12
Astragalus parvulus Bornm.	Fabaceae	EN	3	2428	12
Astragalus patrius Maassoumi	Fabaceae	LC or NT	19	86382	76
Astragalus paucifoliolatus Podlech	Fabaceae	LC or NT	21	55702	84
Astragalus pauperiflorus Bornm.	Fabaceae	LC or NT	17	7986	60
Astragalus pauxillis Maassoumi & F.Ghahremani	Fabaceae	EN	5	9404	20
Astragalus pediculariformis Maassoumi	Fabaceae	EN	4	1963	16
Astragalus pellitus Bunge	Fabaceae	LC or NT	48	519273	192
Astragalus pendulipodus Ranjbar & Karamian	Fabaceae	EN	2	0	8
Astragalus penetratus Maassoumi	Fabaceae	EN	5	6008	16
Astragalus pentanthus Boiss.	Fabaceae	LC or NT	27	96783	108
Astragalus perdurabilis Maassoumi	Fabaceae	DD	NA	NA	NA
Astragalus perdurans Podl.	Fabaceae	EN	7	15394	28
Astragalus pereshkhorranicus Maassoumi & F. Ghahremani	Fabaceae	CR	1	0	4
Astragalus persicus (DC.) Fisch. & C.A. Mey.	Fabaceae	VU	8	23982	32
Astragalus peymanii Maassoumi	Fabaceae	CR	1	0	4
Astragalus pileh-khasehensis Podlech & Maassoumi	Fabaceae	CR	1	0	4
Astragalus piranshahricus Maassoumi & Podl.	Fabaceae	VU	8	4328	32
Astragalus pish-chakensis Maassoumi	Fabaceae	DD	NA	NA	NA
Astragalus plagiophacos Maassoumi & Podlech	Fabaceae	EN	4	48113	16
Astragalus platifoliolatus Maassoumi	Fabaceae	CR	1	0	4
Astragalus platysematus Bunge	Fabaceae	LC or NT	19	17922	72
Astragalus plebejus Boiss.	Fabaceae	LC or NT	13	34711	52

Astragalus pluriflorus F. Ghahrem., Maassoumi, Bagheri & Podlech	Fabaceae	CR	1	0	4
Astragalus podoloboides Maassoumi	Fabaceae	CR	1	0	4
Astragalus podosphaerus Boiss. & Housskn.	Fabaceae	EN	4	2421	16
Astragalus poliotrichus Bornm.	Fabaceae	CR	1	0	4
Astragalus polyanthus Bunge	Fabaceae	EN	6	5229	24
Astragalus polystachys Maassoumi	Fabaceae	CR	1	0	4
Astragalus porphyrophysa Bornm. & Gauba	Fabaceae	LC or NT	15	92432	60
Astragalus pravitzii Podlech	Fabaceae	VU	7	5235	28
Astragalus protectus Maassoumi & Podlech	Fabaceae	CR	1	0	4
Astragalus pseudoantilibani Maassoumi	Fabaceae	DD	NA	NA	NA
Astragalus pseudocomosus Maassoumi	Fabaceae	CR	1	0	4
Astragalus pseudocyclophyllus Rech.f.	Fabaceae	EN	8	1246	28
Astragalus pseudofragiferus Tietz.	Fabaceae	CR	1	0	4
Astragalus pseudoibicinus Maassoumi & Podlech	Fabaceae	LC or NT	16	33140	60
Astragalus pseudoindurascens Sirj. & Rech.f.	Fabaceae	EN	5	7720	20
Astragalus pseudojohannis Maassoumi & Podlech	Fabaceae	EN	4	68	16
Astragalus pseudokurrumensis Sirj. & Rech.f.	Fabaceae	EN	3	2418	12
Astragalus pseudomacrostachys Maassoumi	Fabaceae	EN	4	4114	16
Astragalus pseudonigrescens Maassoumi	Fabaceae	CR	1	0	4
Astragalus pseudo-orthocarpus Ranjbar & Maassoumi	Fabaceae	CR	1	0	4
Astragalus pseudoparalurges F. Ghahrem., Maassoumi, Bagheri & Podlech	Fabaceae	CR	1	0	4
Astragalus pseudopellitus Podl.	Fabaceae	EN	3	2872	12
Astragalus pseudopersicus Maassoumi & Podlech	Fabaceae	EN	3	43	12
Astragalus pseudorobustus Podlech & Maassoumi	Fabaceae	LC or NT	34	48575	128
Astragalus pseudoshebarensis Podlech	Fabaceae	VU	11	55580	44
Astragalus pseudotortuosus Tietz & Zarre	Fabaceae	EN	3	226	12
Astragalus pseudozagrosicus Maassoumi & Podle.	Fabaceae	VU	8	12588	24
Astragalus psilostylus Bunge	Fabaceae	VU	7	14225	28
Astragalus ptychophyllus Boiss. in Kotschy	Fabaceae	LC or NT	34	82551	128

Astragalus punctatus Bunge	Fabaceae	LC or NT	15	9083	60
Astragalus qaratchaicus Maassoumi	Fabaceae	CR	1	0	4
Astragalus qeydarnabiensis Bagheri	Fabaceae	CR	1	0	4
Astragalus qohestanicus Nasseh & Maassoumi	Fabaceae	CR	1	0	4
Astragalus qorvehensis Podlech	Fabaceae	EN	2	0	8
Astragalus qoturensis Podlech	Fabaceae	CR	1	0	4
Astragalus quinquefoliolatus Bunge	Fabaceae	EN	5	11047	20
Astragalus rahiminejadii Ranjbar	Fabaceae	CR	1	0	4
Astragalus rashed-mohasseli Nasseh	Fabaceae	CR	1	0	4
Astragalus rassulovae Podlech	Fabaceae	EN	2	0	8
Astragalus raswendicus Hausskn. & Bornm.	Fabaceae	VU	8	28001	32
Astragalus razensis Nasseh & Joharchi	Fabaceae	CR	1	0	4
Astragalus recognitus Fisch.	Fabaceae	VU	6	14528	24
Astragalus reconditus Podlech & Maassoumi	Fabaceae	CR	1	0	4
Astragalus recurvatus Podlech	Fabaceae	EN	2	0	8
Astragalus regestus Maassoumi	Fabaceae	EN	4	6017	16
Astragalus remotiflorus Boiss.	Fabaceae	LC or NT	24	184764	92
Astragalus remotijugus Boiss. & Hohen. in Boiss. Diagn. Pl. Or. Nov. Ser. 1	Fabaceae	LC or NT	80	13681	284
Astragalus remotispicatus Bagheri & Maassoumi	Fabaceae	CR	1	0	4
Astragalus renzianus Podlech	Fabaceae	VU	8	11011	32
Astragalus repentinus Ekici & Podlech	Fabaceae	VU	11	68014	40
Astragalus reticulato-venosus Maassoumi & Podlech	Fabaceae	CR	1	0	4
Astragalus reuterianus Boiss.	Fabaceae	LC or NT	12	152667	48
Astragalus rhabdophorus Bornm.	Fabaceae	VU	10	21632	40
Astragalus rhodosemius Boiss. & Hausskn.	Fabaceae	LC or NT	104	408378	408
Astragalus rijabensis Maassoumi, Mozaff. & Bagheri	Fabaceae	CR	1	0	4
Astragalus rimarum Bornm.	Fabaceae	EN	3	200	12
Astragalus riouxii Rech.f.	Fabaceae	EN	3	9	8
Astragalus rollovii Grossh	Fabaceae	LC or NT	12	52829	48

Astragalus rosellus Sirj. & Rech.f.	Fabaceae	VU	11	28591	40
Astragalus rubicalyx Maassoumi	Fabaceae	DD	NA	NA	NA
Astragalus rubriflorus Bunge	Fabaceae	LC or NT	34	20348	124
Astragalus rubriphysa Maassoumi & Tayebi	Fabaceae	CR	1	0	4
Astragalus rubrocalycinus Maassoumi & Podl.	Fabaceae	VU	14	6577	56
Astragalus rubrolineatus Sirj. & Rech.f.	Fabaceae	EN	2	0	8
Astragalus rubrostriatus Bunge	Fabaceae	LC or NT	13	75769	52
Astragalus rudimentus Maassoumi	Fabaceae	VU	13	3339	44
Astragalus rufescens Freyn	Fabaceae	LC or NT	15	92315	60
Astragalus runemarkii Maassoumi & Podlech	Fabaceae	CR	1	0	4
Astragalus ruscifolius Boiss.	Fabaceae	LC or NT	20	300380	80
Astragalus saadatabadensis Podlech	Fabaceae	CR	1	0	4
Astragalus sabetii Podlech & Maassoumi	Fabaceae	CR	1	0	4
Astragalus sabzevarensis Podlech & Zarre	Fabaceae	CR	1	0	4
Astragalus saccatus Boiss.	Fabaceae	EN	4	10793	16
Astragalus saetiger Becht	Fabaceae	EN	5	3755	20
Astragalus safavii Podlech & Maassoumi	Fabaceae	EN	5	7834	20
Astragalus sahendi Fisch.	Fabaceae	EN	8	1399	28
Astragalus salavatabadensis Podlech	Fabaceae	CR	1	0	4
Astragalus salehabadensis Ranjbar & Zarin	Fabaceae	CR	1	0	4
Astragalus salmakiae Podlech & Zarre	Fabaceae	CR	1	0	4
Astragalus sanandajianus Tietz	Fabaceae	EN	2	0	8
Astragalus sarabensis Maassoumi & Podlech	Fabaceae	CR	1	0	4
Astragalus saremii Maassoumi	Fabaceae	CR	1	0	4
Astragalus sarzehensis Ranjbar	Fabaceae	CR	1	0	4
Astragalus savanatensis Ranjbar	Fabaceae	CR	1	0	4
Astragalus savellanicus Podlech	Fabaceae	VU	7	14424	28
Astragalus scapiger Ranjbar & Maassoumi	Fabaceae	VU	12	11212	48
Astragalus schmidii Podl.	Fabaceae	EN	4	5547	16
Astragalus sciureus Boiss. & Hohen. in Boiss.	Fabaceae	LC or NT	21	45077	80

Astragalus sclerocladus Bunge	Fabaceae	EN	7	857	24
Astragalus segregatus Zarre & Podlech	Fabaceae	CR	1	0	4
Astragalus seidabadensis Bunge	Fabaceae	EN	4	7160	16
Astragalus semiglabricarpus Maassoumi	Fabaceae	CR	1	0	4
Astragalus semilunatus Podlech	Fabaceae	EN	3	72	12
Astragalus semiromensis Podlech & Maassoumi	Fabaceae	VU	6	12871	24
Astragalus semitarius Zarre & Podlech	Fabaceae	CR	1	0	4
Astragalus semnanensis Bornm. & Rech.f.	Fabaceae	EN	8	82	28
Astragalus senilis Bornm.	Fabaceae	LC or NT	14	55880	56
Astragalus shabilensis Podlech & Maassoumi	Fabaceae	EN	3	166	12
Astragalus shahbazanicus Podlech	Fabaceae	EN	5	25299	20
Astragalus shahinii Podlech & Maassoumi	Fabaceae	CR	1	0	4
Astragalus shahsavaranicus Maassoumi	Fabaceae	DD	NA	NA	NA
Astragalus shahsavarii Maassoumi & Podlech	Fabaceae	EN	4	1737	16
Astragalus shebarensis Podlech	Fabaceae	DD	NA	NA	NA
Astragalus shehbazii Zarre & Podlech	Fabaceae	CR	1	0	4
Astragalus shuturunkuhensis Podlech	Fabaceae	EN	2	0	8
Astragalus siahcheshmehensis Maassoumi & Podlech	Fabaceae	EN	4	148	16
Astragalus simakanensis Maassoumi & Hatami	Fabaceae	CR	1	0	4
Astragalus singarensis Boiss. & Hausskn	Fabaceae	LC or NT	53	314639	196
Astragalus sirjaevii Zarre	Fabaceae	CR	2	0	4
Astragalus sisakhtianus Podlech & Maassoumi	Fabaceae	CR	1	0	4
Astragalus sitiens Bunge	Fabaceae	VU	12	507242	48
Astragalus sivendicus Podlech & Maassoumi	Fabaceae	CR	1	0	4
Astragalus sohrevardianus Bagheri, Maassoumi & F. Ghahrem.	Fabaceae	CR	1	0	4
Astragalus sojakii Podl.	Fabaceae	LC or NT	12	132270	48
Astragalus spachianiformis Podlech & Maassoumi	Fabaceae	EN	3	17233	12
Astragalus spachianus Boiss. & Buhse	Fabaceae	LC or NT	37	452349	144
Astragalus speciosus Boiss. & Hohen.	Fabaceae	VU	15	792	56
Astragalus sphaeranthus Boiss.	Fabaceae	CR	1	0	4

Astragalus stenolepis Fisch.	Fabaceae	LC or NT	21	5076	76
Astragalus stenostegius Boiss. & Hausskn.	Fabaceae	VU	10	45325	40
Astragalus stepporum Podl.	Fabaceae	EN	2	0	8
Astragalus straussii Bornm.	Fabaceae	LC or NT	21	73730	84
Astragalus strictissimus Podlech & Zarre	Fabaceae	CR	1	0	4
Astragalus subalpinus Boiss. Buhse	Fabaceae	VU	11	44717	40
Astragalus subaspadanus Maassoumi	Fabaceae	CR	1	0	4
Astragalus subbrevidens Maassoumi	Fabaceae	EN	2	0	8
Astragalus subglaberrimus Podlech & Maassoumi	Fabaceae	EN	2	0	8
Astragalus subkohrudicus Maassoumi	Fabaceae	CR	1	0	4
Astragalus sublaguriformis Bagheri, Maassoumi & F. Ghahrem.	Fabaceae	CR	1	0	4
Astragalus submitis Boiss. & Hohen.	Fabaceae	LC or NT	35	25040	136
Astragalus subpentanthus Maassoumi & Podlech	Fabaceae	EN	2	0	8
Astragalus subrecognitus Bagheri	Fabaceae	CR	1	0	4
Astragalus subrosulariformis Sirj. & Rech.f.	Fabaceae	CR	1	0	4
Astragalus subverus Maassoumi & Zarre	Fabaceae	DD	NA	NA	NA
Astragalus sumarensis Maassoumi	Fabaceae	CR	1	0	4
Astragalus susianus Boiss.	Fabaceae	LC or NT	33	62886	128
Astragalus sympileicalycinus Maassoumi & Nasseh	Fabaceae	CR	1	0	4
Astragalus sympileicarpus Rech.f.	Fabaceae	VU	8	13236	32
Astragalus tabrizianus Fisch.	Fabaceae	LC or NT	22	59231	84
Astragalus taebiae Zarre & Podlech	Fabaceae	CR	1	0	4
Astragalus tahbaziae Zarre & Podlech	Fabaceae	CR	1	0	4
Astragalus taleshensis Bidarlord, F.Ghahrem. & Maassoumi	Fabaceae	CR	1	0	4
Astragalus talimansurensis Sirj. & Rech.f.	Fabaceae	LC or NT	19	45152	76
Astragalus tarumensis Sirj. & Rech.f.	Fabaceae	LC or NT	15	71780	56
Astragalus tavakolii Maassoumi	Fabaceae	DD	NA	NA	NA
Astragalus teheranicus Boiss & Hohen. In Boiss.	Fabaceae	LC or NT	59	363917	236
Astragalus tekabensis Maasoumi & Maroofi	Fabaceae	CR	1	0	4
Astragalus tekabensis Maassoumi & Maroofi	Fabaceae	CR	2	0	4

Astragalus tenellus Bunge	Fabaceae	VU	8	32651	32
Astragalus tenuiramosus Podlech & Zarre	Fabaceae	VU	8	46336	32
Astragalus tenuiscapus Freyn & Bornm.	Fabaceae	VU	12	120499	48
Astragalus tenuissimus Zarre & Podlech	Fabaceae	CR	1	0	4
Astragalus titziae Ghahreman & Zarre	Fabaceae	EN	4	28	16
Astragalus torbathaydariyehensis Ranjbar & Zarin	Fabaceae	CR	1	0	4
Astragalus touranicus Freitag & Podl.	Fabaceae	VU	10	241908	40
Astragalus townsendii Zarre.	Fabaceae	EN	4	975	16
Astragalus trachyacanthos Fischer	Fabaceae	LC or NT	90	317806	340
Astragalus tricholobus DC.	Fabaceae	LC or NT	23	221452	92
Astragalus turgidus Podlech	Fabaceae	EN	5	6823	16
Astragalus turkman-chaiensis Maassoumi, Mozaff. & Ramezani	Fabaceae	CR	1	0	4
Astragalus tuyehensis Ghahreman	Fabaceae	EN	4	29960	16
Astragalus typhaeformis Maassoumi	Fabaceae	EN	5	5914	20
Astragalus urbanus Podl. & Maassoumi	Fabaceae	EN	3	8	8
Astragalus vanillae Boiss.	Fabaceae	LC or NT	32	399625	128
Astragalus vegetus Bunge	Fabaceae	LC or NT	81	289453	312
Astragalus veiskaramii Zarre	Fabaceae	CR	1	0	4
Astragalus vereskensis Maassoumi & Podl.	Fabaceae	LC or NT	30	32960	120
Astragalus vernaculus Podlech	Fabaceae	VU	6	147521	24
Astragalus vessalae Maassoumi & Podl.	Fabaceae	CR	1	0	4
Astragalus vicinalis Zarre & Podlech	Fabaceae	CR	1	0	4
Astragalus vulcanicus Bornm.	Fabaceae	EN	4	163	16
Astragalus wiesneri Maassoumi & Pahlevani	Fabaceae	CR	1	0	4
Astragalus xiphidiopsis Bornm.	Fabaceae	EN	5	974	20
Astragalus yazdii (Vassilcz.) Podlech & Maassoumi	Fabaceae	EN	5	36430	16
Astragalus yushensis T. Sabaii	Fabaceae	EN	6	84	20
Astragalus zagrosicus Boiss. & Hausskn.	Fabaceae	EN	6	22246	24
Astragalus zangooeianus Maassoumi	Fabaceae	EN	2	0	8
Astragalus zanjanensis Podlech & Maassoumi	Fabaceae	VU	12	14832	48

Astragalus zarreianus Ranjbar	Fabaceae	CR	1	0	4
Astragalus zerdanus Boiss.	Fabaceae	VU	8	21340	28
Astragalus zoshkensis Ghahremaninejad	Fabaceae	EN	5	1713	20
Astragalus zourabadensis Zarre & Podlech	Fabaceae	DD	NA	NA	NA
Asyneuma multicaule (Boiss.) Rech.f. Schiman-Czeina	Campanulaceae	DD	NA	NA	NA
Atractylis delvarii Mozaffarian	Asteraceae	CR	1	0	4
Atraphaxis aucheri Jaub. & Spach	Polygonaceae	EN	3	12	12
Atraphaxis binaludensis S. Tavakkoli	Polygonaceae	CR	1	0	4
Atraphaxis ephedroides S.Tavakkoli, Kaz.Osaloo & Mozaff.	Polygonaceae	EN	2	0	8
Atraphaxis intricata Mozaffarian	Polygonaceae	CR	2	0	4
Atraphaxis radkanensis Tavakkoli, Osaloo & Mozaff.	Polygonaceae	CR	2	0	4
Atraphaxis suaedifolia Jaub & Spach.	Polygonaceae	EN	3	1506	12
Azilia eryngioides (Pau) Hedge & Lamond	Apiaceae	VU	7	6280	28
Ballota platyloma Rech.f.	Lamiaceae	VU	13	25845	52
Bellevalia assadii Wendelbo	Asparagaceae	CR	1	0	4
Bellevalia cyanopoda Wendelbo	Asparagaceae	EN	4	4846	16
Bellevalia decolorans Bornm.	Asparagaceae	VU	10	190678	40
Bellevalia heweri Wendelbo	Asparagaceae	CR	2	0	4
Bellevalia koeiei Rech.f.	Asparagaceae	EN	2	0	8
Bellevalia multicolor Wendelbo	Asparagaceae	CR	2	0	4
Bellevalia shirazana Parsa	Asparagaceae	EN	3	11882	12
Bellevalia tabriziana Turrill	Asparagaceae	EN	5	2230	20
Bellevalia tristis Bornm.	Asparagaceae	VU	9	12757	32
Bellevalia wendelboi Maassoumi & Jafari	Asparagaceae	CR	1	0	4
Biarum platyspathum Bornm.	Araceae	EN	3	763	12
Biarum straussii Engl.	Araceae	LC or NT	12	165340	48
Bienertia kavirense Akhani	Amaranthaceae	CR	1	0	4
Bromus frigidus Boiss. & Hausskn.	Poaceae	EN	3	12223	12
Brossardia papyracea Boiss.	Brassicaceae	LC or NT	19	190342	76
Brotera persica Spreng.	Lamiaceae	DD	NA	NA	NA

Bufonia calycina Boiss. & Hausskn.	Caryophyllaceae	EN	3	1402	12
Bufonia capitata Bornm.	Caryophyllaceae	CR	1	0	4
Bufonia capsularis Boiss. & Hausskn. in Boiss.	Caryophyllaceae	LC or NT	18	90263	68
Bufonia darvishii Zeraatkar	Caryophyllaceae	CR	1	0	4
Bufonia enervis Boiss.	Caryophyllaceae	LC or NT	11	223685	44
Bufonia hebecalyx Boiss.	Caryophyllaceae	CR	1	0	4
Bufonia iranica Z.Rostami, Assadi & F.Ghahrem.	Caryophyllaceae	CR	1	0	4
Bufonia koelzii Rech.f.	Caryophyllaceae	LC or NT	16	14519	60
Bufonia kotschyana Boiss.	Caryophyllaceae	LC or NT	45	292253	172
Bufonia macrocarpa Ser. in DC.	Caryophyllaceae	LC or NT	15	93554	60
Bufonia micrantha Boiss. & Hausskn.	Caryophyllaceae	CR	1	0	4
Bufonia stapfii Bornm.	Caryophyllaceae	CR	1	0	4
Bunium luristanicum Rech.f.	Apiaceae	LC or NT	14	305308	56
Bunium wolffii Kljuykov	Apiaceae	LC or NT	15	127568	60
Bupleurum flexile Bornm. ex Gauba	Apiaceae	VU	10	24258	36
Bupleurum ghahremanii Mozaffarian	Apiaceae	EN	2	0	8
Bupleurum gilanicum Mozaff.	Apiaceae	CR	1	0	4
Bupleurum iranicum Bidarlord & Lyskov	Apiaceae	CR	1	0	4
Bupleurum wolffianum Bornm. in Wolff in Engler	Apiaceae	CR	1	0	4
Caccinia actinobole Bunge	Boraginaceae	EN	4	13798	16
Caccinia kotschyi Boiss.	Boraginaceae	VU	7	74869	28
Caccinia monandra (Bunge) I.M. Johnston	Boraginaceae	DD	NA	NA	NA
Caccinia strigosa Boiss.	Boraginaceae	VU	10	13527	40
Calendula aurantiaca Kotschy ex Boiss.	Asteraceae	CR	1	0	4
Calligonum alatosetosum Maassoumi & Kazempour	Polygonaceae	CR	1	0	4
Calligonum bungei Boiss.	Polygonaceae	LC or NT	14	469496	56
Calligonum denticulatum Bunge ex Boiss.	Polygonaceae	VU	8	375314	32
Calligonum laristanicum Rech.f. & Schiman-Czeika	Polygonaceae	VU	6	41474	24
Calligonum persicum (Boiss. & Buhse) Boiss.	Polygonaceae	VU	10	387268	36
Calligonum schizopterum Rech.f. & Schiman-Czeika	Polygonaceae	EN	5	160891	16

Calligonum spinosetosum Maassoumi & Batooli	Polygonaceae	CR	1	0	4
Calligonum stenopterum Bunge ex Boiss.	Polygonaceae	EN	4	65552	16
Camelinopsis alborzica Doostmohammadi	Brassicaceae	CR	1	0	4
Campanula candida DC.	Campanulaceae	VU	10	15515	40
Campanula escalerae Rech.f. & Schiman-Czeika	Campanulaceae	CR	1	0	4
Campanula gilliatii Milne-Redh.	Campanulaceae	EN	3	85	12
Campanula hermannii Rech.f.	Campanulaceae	EN	2	0	8
Campanula humillima DC.	Campanulaceae	LC or NT	15	47996	56
Campanula hystricula Pau	Campanulaceae	CR	1	0	4
Campanula kermanica (Rech.f.	Campanulaceae	VU	7	9866	28
Campanula kurdistanica Advay & Maroofi	Campanulaceae	CR	1	0	4
Campanula lamondiae Rech.f. Akad. Mus. Naturwiss. Log: 173 (1973).	Campanulaceae	EN	2	0	4
Campanula lourica Boiss.	Campanulaceae	LC or NT	22	16927	80
Campanula luristanica Freyn	Campanulaceae	EN	10	15693	36
Campanula oreodoxa Arjmandi & Memariani	Campanulaceae	EN	2	0	8
Campanula persepolitana Ky. in Boiss.	Campanulaceae	EN	5	72890	20
Centaurea alfonsoi Negaresh	Asteraceae	CR	1	0	4
Centaurea amadanensis Sch.Bip.	Asteraceae	LC or NT	39	154328	156
Centaurea ardabilica Ranjbar & Heydari	Asteraceae	CR	1	0	4
Centaurea aucheri Wagenitz	Asteraceae	LC or NT	40	328252	160
Centaurea aziziana Rech.f.	Asteraceae	VU	10	89552	40
Centaurea bachtiarica Hayek & Bornm.	Asteraceae	CR	1	0	4
Centaurea bavegehensis Ranjbar & Negaresh	Asteraceae	CR	1	0	4
Centaurea congesta Wagenitz	Asteraceae	VU	8	11553	32
Centaurea daneshvarii Negaresh & Oreizi	Asteraceae	DD	NA	NA	NA
Centaurea dezfulica Ranjbar & S.Mohamadi	Asteraceae	CR	1	0	4
Centaurea elbursensis Boiss. & Buhse	Asteraceae	EN	2	0	8
Centaurea elymaitica Mozaff.	Asteraceae	CR	1	0	4
Centaurea gabrielae (Bornm.) Wagenitz	Asteraceae	VU	11	44262	44
Centaurea galactochroa Rech.f.	Asteraceae	EN	3	12	12

Centaurea gaubae (Bornm.) Wagenitz	Asteraceae	LC or NT	19	102998	76
Centaurea geluensis Boiss. & Hausskn.	Asteraceae	EN	2	0	4
Centaurea ghahremanii Wagenitz & Esfand	Asteraceae	CR	1	0	4
Centaurea gilanica Bornm.	Asteraceae	VU	12	78679	40
Centaurea golestanica Akhani & Wagenitz	Asteraceae	CR	1	0	4
Centaurea incanescens (DC.) Sch.Bip.	Asteraceae	VU	8	4826	32
Centaurea intricata Boiss.	Asteraceae	LC or NT	22	205138	84
Centaurea ispahanica Boiss.	Asteraceae	LC or NT	25	137065	100
Centaurea kabirkuhensis Mozaff., F. Ghahrem. & Fereid.	Asteraceae	CR	1	0	4
Centaurea kamalnejadii Negaresh	Asteraceae	CR	2	0	4
Centaurea kamyaranensis Ranjbar & Negaresh	Asteraceae	CR	1	0	4
Centaurea kandavanensis Wagenitz	Asteraceae	EN	8	3065	24
Centaurea karamianiae Negaresh	Asteraceae	EN	2	0	8
Centaurea khuzestanica Mozaffarian	Asteraceae	CR	1	0	4
Centaurea kuhdashtensis Ranjbar & S.Mohamadi	Asteraceae	CR	1	0	4
Centaurea lachnopus Rech.f.	Asteraceae	EN	3	8	8
Centaurea luristanica Rech.f.	Asteraceae	EN	5	28574	20
Centaurea masjedsoleymanensis Ranjbar & Askari	Asteraceae	CR	1	0	4
Centaurea microlonchoides Boiss.	Asteraceae	VU	6	166634	24
Centaurea mozaffarianii Negaresh	Asteraceae	CR	1	0	4
Centaurea orumiensis Ranjjbar & Negaresh	Asteraceae	CR	1	0	4
Centaurea pabotii Wagenitz	Asteraceae	VU	7	15323	28
Centaurea palanganensis Ranjbar & Askari	Asteraceae	CR	1	0	4
Centaurea paradoxa Mozaffarian	Asteraceae	CR	1	0	4
Centaurea procera Mozaff.	Asteraceae	CR	1	0	4
Centaurea rahiminejadii Negaresh	Asteraceae	CR	1	0	4
Centaurea ravansarensis Ranjbar & Negaresh	Asteraceae	CR	1	0	4
Centaurea salmasensis Ranjbar & Heydari	Asteraceae	CR	1	0	4
Centaurea sanandajensis Ranjbar & Negaresh	Asteraceae	CR	1	0	4
Centaurea schmidii Wagenitz	Asteraceae	CR	1	0	4

Centaurea serowensis Negaresh	Asteraceae	DD	NA	NA	NA
Centaurea shahuensis Ranjbar & Negaresh	Asteraceae	CR	1	0	4
Centaurea shehbazii Ranjbar & Negaresh	Asteraceae	CR	1	0	4
Centaurea solitaria Ranjbar & Negaresh	Asteraceae	CR	1	0	4
Centaurea tabriziana Ranjbar & Heydari	Asteraceae	CR	1	0	4
Centaurea urvillei DC.	Asteraceae	LC or NT	13	60743	52
Centaurea ustulata DC.	Asteraceae	LC or NT	19	149771	76
Centaurea wendelboi Wagenitz.	Asteraceae	EN	2	0	8
Centaurea xeranthemoides Rech.f.	Asteraceae	EN	6	8495	20
Centaurea zagrosmontana Ranjbar & Heydari	Asteraceae	CR	1	0	4
Centaurea zangulensis ranjbar & negaresh	Asteraceae	CR	1	0	4
Cephalaria bojnordensis Ranjbar & Z.Ranjbar	Caprifoliaceae	CR	1	0	4
Cephalaria chaldoranensis Ranjbar & Z. Ranjbar	Caprifoliaceae	CR	1	0	4
Cephalaria golestanica Ranjbar & Z. Ranjbar	Caprifoliaceae	EN	3	19	8
Cephalaria integerrima (Bornmüller) Ranjbar & Z. Ranjbar	Caprifoliaceae	CR	1	0	4
Cephalaria juncea Boiss.	Caprifoliaceae	LC or NT	13	108712	48
Cephalaria kleinii Ranjbar & Ranjbar	Caprifoliaceae	CR	1	0	4
Cephalaria kurdistanica Maroofi, Tabad & Rastegar	Caprifoliaceae	DD	NA	NA	NA
Cephalaria qeydarensis Ranjbar & Z. Ranjbar	Caprifoliaceae	CR	1	0	4
Cephalaria subindivisa (Boissier) Szabó	Caprifoliaceae	EN	3	52	12
Cephalaria torbatejamensis Ranjbar & Z. Ranjbar	Caprifoliaceae	EN	3	3628	12
Cephalorrhynchus brassicifolius (Boiss.) Tuisl	Asteraceae	LC or NT	35	134113	136
Cephalorrhynchus gorganicus (Rech.f. & Esfand.) Tuisl	Asteraceae	EN	3	11001	12
Cephalorrhynchus microcephalus (DC.) Schchian	Asteraceae	LC or NT	57	368590	228
Cerastium alvandicum Poursakhi, Assadi & F.Ghahrem.	Caryophyllaceae	CR	1	0	4
Cerastium assadii Poursakhi & F.Ghahrem.	Caryophyllaceae	CR	1	0	4
Cerastium bazofticum Poursakhi, Assadi & F.Ghahrem.	Caryophyllaceae	CR	1	0	4
Cerastium cylindricum Poursakhi & Assadi	Caryophyllaceae	CR	1	0	4
Cerastium persicum Boiss.	Caryophyllaceae	EN	3	4449	12
Cerasus brachypetala Boiss.	Rosaceae	LC or NT	26	172971	104

Cerasus chorassanica Pojark.	Rosaceae	EN	4	3801	16
Cerasus paradoxa Dehshiri & Mozaff.	Rosaceae	CR	1	0	4
Cerasus yazdiana Mozaff.	Rosaceae	CR	1	0	4
Chaenorhinum foroughii Speta	Plantaginaceae	CR	1	0	4
Chaenorhinum grossecostatum Speta	Plantaginaceae	EN	6	22019	20
Chaerophyllum nivale Hedge & Lamond	Apiaceae	EN	5	308	20
Chamaegeron asterellus (Bornm.) Botsch.	Asteraceae	VU	8	28932	28
Chamaegeron keredjensis (Bornm. & Gauba) Grierson	Asteraceae	CR	1	0	4
Chenopodium khorasanica Hamdi & Malekloo	Amaranthaceae	CR	1	0	4
Chesneya kotschyi Boiss.	Fabaceae	EN	4	518	16
Cicer spiroceras Jaub. & Spach	Fabaceae	LC or NT	49	331041	184
Cicer stapfianum Rech.f.	Fabaceae	CR	2	0	4
Cicer subaphyllum Boiss.	Fabaceae	EN	3	224	12
Cicer tragacanthoides Jaub. & Spach	Fabaceae	LC or NT	21	436575	76
Cicerbita polyclada (Boiss.) Beauverd	Asteraceae	CR	1	0	4
Cirsium bracteosum DC.	Asteraceae	EN	3	23368	12
Cirsium gadukense Petrak	Asteraceae	CR	1	0	4
Cirsium iranicum Petrak	Asteraceae	CR	1	0	4
Cirsium lappaceum M.Bieb.	Asteraceae	LC or NT	18	120521	72
Cirsium pyramidale Bornm.	Asteraceae	EN	2	0	8
Cirsium spectabile DC.	Asteraceae	LC or NT	14	181674	56
Clastopus vestitus (Desv.) Boiss.	Brassicaceae	LC or NT	14	62971	52
Clematis iranica Habibi, Ghorbani & Azizian	Ranunculaceae	CR	1	0	4
Clematis khuzestanica Dinarvand	Ranunculaceae	CR	1	0	4
Cleome foliolosa DC.	Capparaceae	EN	4	80634	16
Codonocephalum stenocalathium Rech.f.	Asteraceae	CR	1	0	4
Colchicum bakhtiaricum Matin & Iranshahr	Colchicaceae	CR	1	0	4
Colchicum varians (Freyn & Bornm.) Czernjak.	Colchicaceae	LC or NT	20	127559	80
Colchicum wendelboi K. Persson	Colchicaceae	VU	9	59510	36
Colpodium gillettii Bor	Poaceae	CR	2	0	4

Colutea gifana Parsa	Fabaceae	CR	1	0	4
Colutea persica Boiss.	Fabaceae	LC or NT	18	562700	72
Colutea porphyrogramma Rech.f.	Fabaceae	LC or NT	12	10638	48
Colutea uniflora C. Back in Stapf	Fabaceae	EN	4	338	16
Consolida linarioides (Boiss.) Munz	Ranunculaceae	EN	4	5676	16
Consolida lorestanica Iranshahr	Ranunculaceae	EN	3	287	12
Consolida teheranica (Boiss.) Rech.f.	Ranunculaceae	LC or NT	20	49821	76
Consolida trigonelloides (Boiss.) Munz	Ranunculaceae	LC or NT	12	61350	48
Convolvulus ammocharis Boiss. et Hausskn.	Convolvulaceae	EN	2	0	8
Convolvulus argyracanthus Rech.f.	Convolvulaceae	DD	NA	NA	NA
Convolvulus bazmanensis Ranjbar, Ezazi & F. Ghahrem.	Convolvulaceae	CR	1	0	4
Convolvulus cephalophorus Boiss.	Convolvulaceae	EN	6	17937	24
Convolvulus elymaiticus Mozaff. Iran. J. Bot 16(2): 208 (2010)	Convolvulaceae	CR	1	0	4
Convolvulus eremophilus Boiss. et Buhse	Convolvulaceae	LC or NT	32	421913	128
Convolvulus gonocladus Boiss.	Convolvulaceae	LC or NT	39	183848	156
Convolvulus gracillimus Rech.f.	Convolvulaceae	DD	NA	NA	NA
Convolvulus hormodensis Ranjbar & Ezazi	Convolvulaceae	CR	1	0	4
Convolvulus iranicus J.R.I.Wood & Scotland	Convolvulaceae	CR	1	0	4
Convolvulus koieanus Bornm. ex Koeie	Convolvulaceae	EN	3	4273	12
Convolvulus oxysepalus Boiss.	Convolvulaceae	LC or NT	24	256702	96
Convolvulus schirazianus Boiss.	Convolvulaceae	VU	10	65885	40
Convolvulus stapfii Rech.f.	Convolvulaceae	VU	10	19737	40
Convolvulus turrillianus Parsa	Convolvulaceae	LC or NT	20	198782	76
Convolvulus urosepalus Pau	Convolvulaceae	VU	6	17254	24
Corydalis firouzii Wendelbo	Papaveraceae	CR	1	0	4
Cotoneaster assadii Khatamsaz	Rosaceae	EN	5	42	12
Cotoneaster esfandiarii Khatamsaz	Rosaceae	CR	1	0	4
Cotoneaster mazandaranicus R. Niaki & Attar	Rosaceae	CR	1	0	4
Cotoneaster nima-yushiji R. Niaki & Attar	Rosaceae	CR	1	0	4
Cotoneaster persicus Pojark.	Rosaceae	LC or NT	37	205427	144

Cousinia adenostegia Rech.f.	Asteraceae	EN	2	0	8
Cousinia adenosticta Bornm.	Asteraceae	EN	12	378	32
Cousinia aggregata DC.	Asteraceae	LC or NT	30	60604	112
Cousinia akredii Bornm. & Gauba	Asteraceae	EN	5	555	20
Cousinia albescens C. Winkl. & Strauss	Asteraceae	EN	3	332	12
Cousinia albida DC.	Asteraceae	EN	2	0	8
Cousinia alexeenkoana Bornm.	Asteraceae	VU	9	104004	36
Cousinia alfredii Bornm. & Gauba	Asteraceae	CR	1	0	4
Cousinia amicorum Tscherneva	Asteraceae	CR	1	0	4
Cousinia amplissima (Boiss.) Boiss.	Asteraceae	LC or NT	21	183381	84
Cousinia arakensis Attar & Djavadi	Asteraceae	VU	9	6428	36
Cousinia araneosa DC.	Asteraceae	LC or NT	29	124563	108
Cousinia archibaldii Rech.f.	Asteraceae	EN	8	7885	32
Cousinia arctotidifolia Bunge	Asteraceae	LC or NT	15	148178	60
Cousinia ardalensis Attar & Djavadi	Asteraceae	EN	4	126	12
Cousinia argentea Mehregan & Assadi	Asteraceae	EN	4	19	16
Cousinia assadii Attar	Asteraceae	EN	2	0	8
Cousinia assyriaca Jaub. & Spech	Asteraceae	LC or NT	15	35953	60
Cousinia atrobracteata Attar	Asteraceae	CR	1	0	4
Cousinia atropatana Bunge	Asteraceae	EN	2	0	8
Cousinia attariae Assadi & Joharchi	Asteraceae	EN	3	14	12
Cousinia azerbaidjanica Djavadi	Asteraceae	CR	1	0	4
Cousinia bachtiarica Boiss. & Hausskn.	Asteraceae	VU	8	10287	32
Cousinia barbeyi C.Winkl.	Asteraceae	VU	6	14192	24
Cousinia barezica Assadi	Asteraceae	CR	1	0	4
Cousinia bazoftensis Attar	Asteraceae	EN	3	299	12
Cousinia beauverdiona Bornm.	Asteraceae	CR	1	0	4
Cousinia belangeri DC.	Asteraceae	LC or NT	39	103932	152
Cousinia bienerti Bunge	Asteraceae	EN	3	33	12
Cousinia bijarensis Rech.f.	Asteraceae	EN	4	1984	16

Cousinia bobekii Rech.f.	Asteraceae	LC or NT	20	4771	80
Cousinia boissieri Buhse	Asteraceae	EN	2	0	8
Cousinia bornmulleriana Attar & Maroofi	Asteraceae	CR	1	0	4
Cousinia boyerahmadica Rastegar, Attar & Mirtadzadini	Asteraceae	EN	2	0	8
Cousinia brevicaulis Attar, Mozaff. & Mirtadz.	Asteraceae	CR	1	0	4
Cousinia calcitrapa Boiss.	Asteraceae	LC or NT	45	239404	180
Cousinia calocephala Jaub. & Spech	Asteraceae	LC or NT	209	329824	780
Cousinia calolepis Boiss.	Asteraceae	EN	4	6558	16
Cousinia candolleana Jaub. & Spech	Asteraceae	EN	2	0	8
Cousinia canescens DC.	Asteraceae	EN	5	53824	20
Cousinia cavarae Bornm.	Asteraceae	CR	1	0	4
Cousinia chaetocephala Kult.	Asteraceae	VU	6	1080	24
Cousinia chamaepeuce Boiss.	Asteraceae	VU	7	2105	28
Cousinia chlorocephala C. A. May.	Asteraceae	CR	1	0	4
Cousinia chlorosphaera Bornm.	Asteraceae	LC or NT	17	8332	64
Cousinia chrysacantha Jaub. & Spech	Asteraceae	LC or NT	15	24645	60
Cousinia chrysandra Bornm. & Gauba	Asteraceae	CR	1	0	4
Cousinia commutata Bunge	Asteraceae	VU	10	6411	36
Cousinia concinna Boiss. & Hausskn.	Asteraceae	LC or NT	28	7756	100
Cousinia concolor Bunge	Asteraceae	EN	2	0	8
Cousinia contumax C. Winkl. & Bornm.	Asteraceae	EN	2	0	8
Cousinia cordifolia Djavadi & Attar	Asteraceae	CR	1	0	4
Cousinia crassipes Kult.	Asteraceae	CR	1	0	4
Cousinia crispa Jaub. & Spech	Asteraceae	LC or NT	46	11046	128
Cousinia curvibracteata Mehregan	Asteraceae	EN	2	0	8
Cousinia cylindracea Boiss.	Asteraceae	LC or NT	41	336507	160
Cousinia cylindrocephala Jaub. & Spach	Asteraceae	CR	1	0	4
Cousinia czerniakowskae Kult.	Asteraceae	CR	1	0	4
Cousinia dalahuensis Attar & Ghahreman	Asteraceae	EN	2	0	8
Cousinia dasylepis Kult.	Asteraceae	CR	1	0	4

Cousinia decipiens Boiss. & Hohen.	Asteraceae	VU	8	3412	32
Cousinia decumbens Rech.f.	Asteraceae	CR	3	8	8
Cousinia deluensis Attar, Mozaffarian & Mirtadzadini	Asteraceae	CR	2	0	4
Cousinia denaensis Attar & Djavadi	Asteraceae	EN	5	1869	20
Cousinia deserti Bunge	Asteraceae	CR	1	0	4
Cousinia diezii Rech.f.	Asteraceae	EN	2	0	8
Cousinia dipterocarpa Bornm. & Rech.f.	Asteraceae	EN	2	0	8
Cousinia discolor Bunge	Asteraceae	CR	1	0	4
Cousinia disfulensis Bornm.	Asteraceae	CR	1	0	4
Cousinia eburnea Bornm.	Asteraceae	CR	2	0	4
Cousinia edmondsonii Rech.f.	Asteraceae	EN	2	0	8
Cousinia ekbatanensis Bornm.	Asteraceae	EN	6	8136	24
Cousinia elata Boiss. & Buhse	Asteraceae	LC or NT	30	90738	116
Cousinia elburzensis Attar	Asteraceae	CR	1	0	4
Cousinia elymaitica Attar, Mozaffarian & Mirtadzadini	Asteraceae	CR	1	0	4
Cousinia erinacea Jaub. & Spach	Asteraceae	EN	6	388	24
Cousinia eriobasis Bunge	Asteraceae	VU	10	31083	40
Cousinia eriophylla (Kult.) Bornm.	Asteraceae	EN	2	0	8
Cousinia eriorhiza Bornm.	Asteraceae	CR	2	0	4
Cousinia esfandiarii Rech.f. & Aell.	Asteraceae	VU	11	45331	44
Cousinia euchlora Bornm. & Rech.f.	Asteraceae	CR	1	0	4
Cousinia fabrorum Rech.f.	Asteraceae	LC or NT	12	140072	48
Cousinia falcinella Bornm.	Asteraceae	CR	1	0	4
Cousinia farimanensis Assadi	Asteraceae	CR	1	0	4
Cousinia firuzkuhensis Rech.f.	Asteraceae	EN	2	0	8
Cousinia fragilis C. Winkl. & Bornm.	Asteraceae	CR	2	0	4
Cousinia fragillima Rech.f.	Asteraceae	CR	1	0	4
Cousinia gaharensis Attar & Djavadi	Asteraceae	CR	1	0	4
Cousinia gatchsaranica Mehregan	Asteraceae	EN	3	846	12
Cousinia gaubae Bornm.	Asteraceae	EN	6	750	24

Cousinia gedrosiaca Bornm.&Gauba	Asteraceae	EN	4	6143	16
Cousinia ghahremanii Mirtadz. & Attar	Asteraceae	CR	2	0	4
Cousinia gilanica Bornm.	Asteraceae	EN	3	9308	12
Cousinia gilliatii Rech.f.	Asteraceae	EN	3	35	12
Cousinia ginuae Attar	Asteraceae	EN	2	0	8
Cousinia glaucopsis Bornm. & Rech.f.	Asteraceae	EN	3	4744	12
Cousinia gmelini C. Winkl.	Asteraceae	EN	4	3450	16
Cousinia golestanica Attar	Asteraceae	EN	1	0	4
Cousinia gracilis Boiss.	Asteraceae	VU	8	31377	32
Cousinia hablitzii C. A. MEY.	Asteraceae	CR	1	0	4
Cousinia hamosa C. A. MEY.	Asteraceae	EN	2	0	8
Cousinia harazensis Rech.f.	Asteraceae	EN	5	47	20
Cousinia hazarensis Mirtadzadini & Attar	Asteraceae	CR	1	0	4
Cousinia heliantha Bunge	Asteraceae	EN	5	14540	20
Cousinia hergtiana Bornm.	Asteraceae	EN	4	4822	16
Cousinia hololeuca Bunge	Asteraceae	CR	1	0	4
Cousinia horrida Kult.	Asteraceae	EN	2	0	4
Cousinia hypochionea Bornm.	Asteraceae	CR	2	0	4
Cousinia hypoleuca Boiss.	Asteraceae	LC or NT	12	91122	48
Cousinia ilicifolia Jaub. & Spech	Asteraceae	CR	1	0	4
Cousinia incompta DC.	Asteraceae	CR	1	0	4
Cousinia inflata Boiss. & Hausskn.	Asteraceae	CR	1	0	4
Cousinia iranica C. Winkl. & Strauss.	Asteraceae	EN	2	0	8
Cousinia iranshahriana Attar & Maroofi	Asteraceae	EN	4	5094	16
Cousinia iranshahrii Rech.f.	Asteraceae	CR	1	0	4
Cousinia irritans Rech.f.	Asteraceae	VU	11	78249	40
Cousinia isfahanica Assadi	Asteraceae	CR	1	0	4
Cousinia Jaeobsii Rech.f.	Asteraceae	EN	2	0	4
Cousinia joharchii Assadi & Mehregan	Asteraceae	CR	1	0	4
Cousinia kadereitii Mehregan & Assadi	Asteraceae	CR	2	0	4

Cousinia kandavanensis Attar	Asteraceae	EN	1	0	4
Cousinia karkasensis Mehregan & Djavadi	Asteraceae	EN	3	12	12
Cousinia kashanensis Rech.f. & Esfand.	Asteraceae	CR	1	0	4
Cousinia keredjensis Bornm. & Gauba	Asteraceae	EN	4	48	16
Cousinia kermanensis Mirtadzadini & Attar	Asteraceae	CR	1	0	4
Cousinia kermanshahensis Attar, Ghahreman & Assadi	Asteraceae	EN	2	0	8
Cousinia khorasanica Djavadi & Attar	Asteraceae	CR	1	0	4
Cousinia kilouyensis Djavadi & Attar	Asteraceae	CR	1	0	4
Cousinia komidjanensis Mehregan	Asteraceae	CR	1	0	4
Cousinia kornhuberi Heimerl	Asteraceae	EN	4	11934	16
Cousinia korowiakowi C.Winkl.	Asteraceae	CR	1	0	4
Cousinia kotschyi Boiss.	Asteraceae	LC or NT	90	540316	360
Cousinia kurdistanica Attar	Asteraceae	CR	1	0	4
Cousinia labrorum Rech.f.	Asteraceae	EN	2	0	8
Cousinia lactiflora Rech.f.	Asteraceae	CR	1	0	4
Cousinia lasiandra Bunge	Asteraceae	VU	7	13564	28
Cousinia lasiolepis Boiss.	Asteraceae	EN	4	101676	16
Cousinia lepida Bunge ex Boiss.	Asteraceae	VU	7	4234	28
Cousinia lignosissima Rech.f.	Asteraceae	CR	1	0	4
Cousinia longibracteata Attar & Mirtadz.	Asteraceae	CR	1	0	4
Cousinia longifolia C. Winkl. & Bornm.	Asteraceae	LC or NT	12	137194	44
Cousinia lordeganensis Mehregan	Asteraceae	EN	3	12	12
Cousinia lucida DC.	Asteraceae	EN	2	0	8
Cousinia lurestanica Attar & Djavadi	Asteraceae	CR	1	0	4
Cousinia lurorum (Bornm.) Bornm.	Asteraceae	LC or NT	26	34392	88
Cousinia maassoumii Assadi	Asteraceae	EN	2	0	8
Cousinia macrocephala C. A. Mey.	Asteraceae	LC or NT	26	50553	100
Cousinia maeroptera C. A. MEY.	Asteraceae	EN	5	13548	20
Cousinia manouchehrii Rech.f. & Esfan.	Asteraceae	CR	1	0	4
Cousinia masulehensis Attar & Rastegar	Asteraceae	EN	3	41	12

Cousinia mehreganii Assadi	Asteraceae	EN	2	0	8
Cousinia meluarmanica Rech.f.	Asteraceae	EN	4	1214	16
Cousinia meshhedensis Bornm. & Rech.f.	Asteraceae	VU	9	67826	32
Cousinia microcephala C. A. Mey.	Asteraceae	VU	7	1092	28
Cousinia minuticapitata Attar	Asteraceae	CR	1	0	4
Cousinia mobayenii Ghahreman & Attar	Asteraceae	EN	3	74	12
Cousinia monocephala Bunge	Asteraceae	EN	3	33	12
Cousinia mozdouranensis Djavadi & Attar	Asteraceae	EN	2	0	8
Cousinia mutehensis Rech.f.	Asteraceae	CR	1	0	4
Cousinia nekarmanica Rech.f.	Asteraceae	EN	3	662	12
Cousinia neurocentra Bunge	Asteraceae	VU	7	67403	28
Cousinia noeana Boiss.	Asteraceae	VU	12	3868	48
Cousinia nujianensis Attar	Asteraceae	CR	1	0	4
Cousinia oligocephala Boiss.	Asteraceae	EN	3	559	12
Cousinia orthoclada Hausskn. & Bornm.	Asteraceae	VU	12	22597	44
Cousinia oshtorankuhensis Attar	Asteraceae	EN	2	0	8
Cousinia ottonis Bornm.	Asteraceae	CR	2	0	4
Cousinia papillosa Djavadi & Attar	Asteraceae	CR	1	0	4
Cousinia parsana Ghahreman, Iranshahr & Attar	Asteraceae	CR	1	0	4
Cousinia pasargardensis Attar	Asteraceae	CR	1	0	4
Cousinia pergamacea	Asteraceae	LC or NT	48	17103	176
Cousinia persica Djavadi & Attar	Asteraceae	CR	1	0	4
Cousinia perspolitana Attar & Ghahr.	Asteraceae	CR	3	8	8
Cousinia pinarocephala boiss	Asteraceae	LC or NT	23	65968	92
Cousinia piptocephala Bunge	Asteraceae	LC or NT	18	383840	72
Cousinia platyacantha Bunge	Asteraceae	EN	3	1184	12
Cousinia platyptera Bornm. & C. Winkl.	Asteraceae	CR	1	0	4
Cousinia platyraphis Kult.	Asteraceae	CR	1	0	4
Cousinia pseudocandolleana Assadi	Asteraceae	CR	1	0	4
Cousinia pugionifera Jaub. & Spech	Asteraceae	VU	9	14041	36

Cousinia pulcherantha Attar & Mirtadzadini	Asteraceae	EN	3	8	8
Cousinia qarehbilensis Rech.f.	Asteraceae	CR	1	0	4
Cousinia raphiocephala Rech f.	Asteraceae	EN	4	114	16
Cousinia raphiostegia Rech.f.	Asteraceae	CR	1	0	4
Cousinia rechingerae Bornm.	Asteraceae	VU	6	2392	24
Cousinia rechingerorum Bornm.	Asteraceae	CR	1	0	4
Cousinia recurvata DC.	Asteraceae	VU	8	170866	32
Cousinia remingerorum Bornm.	Asteraceae	CR	1	0	4
Cousinia renominata Rech.f.	Asteraceae	CR	1	0	4
Cousinia rhabdodes Bornm. & Rech.f.	Asteraceae	CR	1	0	4
cousinia rhaphiocephala Rech.f.	Asteraceae	CR	1	0	4
cousinia sabalanica Attar	Asteraceae	CR	1	0	4
Cousinia sabzavarensis Rech.f.	Asteraceae	EN	2	0	8
Cousinia sagittata C.Winkl. & Strauss	Asteraceae	LC or NT	84	39470	308
Cousinia sahandica Attar & Djavadi	Asteraceae	EN	3	1241	12
Cousinia sakawensis Boiss. & Hausskn.	Asteraceae	CR	1	0	4
Cousinia saloukensis Mehregan	Asteraceae	CR	1	0	4
Cousinia sardashtensis Rech.f.	Asteraceae	CR	1	0	4
Cousinia sarzehensis Attar	Asteraceae	CR	1	0	4
Cousinia schindleriana Bornm. & Gauba	Asteraceae	CR	1	0	4
Cousinia schiraziana Attar	Asteraceae	EN	2	0	8
Cousinia seidlitzii Bunge	Asteraceae	VU	10	49814	40
Cousinia semnanensis Attar, Mirtadz. & Sotoodeh	Asteraceae	EN	2	0	8
Cousinia shahuensis Attar	Asteraceae	CR	1	0	4
Cousinia shahvarica Rech.f.	Asteraceae	VU	16	15853	60
Cousinia sharifii Attar & Amini Rad	Asteraceae	CR	1	0	4
Cousinia shebliensis Ghahreman, Iranshahr & Attar	Asteraceae	CR	1	0	4
Cousinia sheidaii Attar	Asteraceae	CR	2	0	4
Cousinia shulabadensis Attar & Ghahreman	Asteraceae	EN	3	85	12
Cousinia sicigera C. Winkl. & Bornm.	Asteraceae	VU	8	20020	32

Cousinia silvanica Attar	Asteraceae	CR	1	0	4
Cousinia silyboides Jaub. & Spach	Asteraceae	LC or NT	15	31536	60
Cousinia spathulata Kult.	Asteraceae	CR	1	0	4
Cousinia sphaerocephala Jaub. & Spech	Asteraceae	VU	9	271	32
Cousinia stahliana Bornm. & Gauba	Asteraceae	VU	12	47083	40
Cousinia straussii Hausskn. & Winkl. ex Winkl.	Asteraceae	EN	5	11237	20
Cousinia subinflata Bornm.	Asteraceae	CR	1	0	4
Cousinia subpectinata Mirtadz.	Asteraceae	CR	1	0	4
Cousinia tabriziana Bunge	Asteraceae	VU	11	8132	44
Cousinia taybadensis Djavadi & Attar	Asteraceae	CR	1	0	4
Cousinia tenuifolia C. A. Mey.	Asteraceae	LC or NT	11	27553	44
Cousinia tenuiramula Rech.f.	Asteraceae	CR	2	0	4
Cousinia termei Rech.f.	Asteraceae	CR	1	0	4
Cousinia tetanocephala Bornm. & Gauba	Asteraceae	CR	1	0	4
Cousinia thamnodes Borss. & Hausskn.	Asteraceae	EN	3	7088	12
Cousinia touchalensis Attar	Asteraceae	CR	1	0	4
Cousinia trachylepis Bunge	Asteraceae	VU	9	1321	36
Cousinia trachyphyllaria Bornm. & Rech.f. .	Asteraceae	EN	4	705	16
Cousinia urumiensis Bornm.	Asteraceae	LC or NT	18	53193	72
Cousinia verbascifolia Bunge	Asteraceae	LC or NT	41	167387	164
Cousinia wendelboi Rech.f.	Asteraceae	CR	1	0	4
Cousinia wilhelminae Rech.f.	Asteraceae	EN	3	332	12
Cousinia xiphiolepis Boiss.	Asteraceae	EN	7	234	24
Cousinia yasujensis Attar	Asteraceae	CR	1	0	4
Cousinia zardkuhensis Attar & Ghahreman	Asteraceae	EN	3	166	12
Crataegus aminii Khatamsaz	Rosaceae	CR	1	0	4
Crataegus assadii Khatamsaz	Rosaceae	CR	1	0	4
Crataegus babakhanloui Khatamsaz	Rosaceae	EN	6	27419	24
Crataegus coriifolia Sharifnia & Zarrinkolah	Rosaceae	EN	4	1792	16
Crataegus grossidentata Sharifnia & K. I. Chr	Rosaceae	EN	3	52	12

Crataegus hatamii Hamzeh ' ee	Rosaceae	EN	4	29	12
Crataegus khatamsazae Hamzeh ' ee	Rosaceae	EN	4	2433	16
Crataegus persica A.Pojark.	Rosaceae	EN	3	2184	12
Crataegus zagrica Khatamsaz	Rosaceae	EN	3	306	12
Crataegus zarrei Donmez	Rosaceae	CR	1	0	4
Crepis alfredii Bornm.	Asteraceae	CR	1	0	4
Crepis demavendi Bornm.	Asteraceae	EN	5	513	20
Crepis elbrusensis Boiss.	Asteraceae	LC or NT	15	144028	52
Crepis elymaitica Bornm.	Asteraceae	EN	4	9377	16
Crepis frigida (Boiss. & Balansa) Babc	Asteraceae	DD	NA	NA	NA
Crepis gaubae Bornm.	Asteraceae	EN	3	38	12
Crepis heterotricha DC.	Asteraceae	VU	11	208387	44
Crepis khorassanica Boiss.	Asteraceae	EN	4	81667	16
Crepis semnanensis N. Heidarnia & M. Assadi	Asteraceae	EN	2	0	8
Crepis straussii Bornm.	Asteraceae	VU	9	38099	36
Crocus almehensis Brickell & Mathew	Iridaceae	CR	1	0	4
Crocus azerbaijanicus Dolatyari & Rukšāns	Iridaceae	CR	1	0	4
Crocus chiaicus Dolatyari & Rukšāns	Iridaceae	CR	1	0	4
Crocus chionophilus Dolatyari & Rukšāns	Iridaceae	CR	1	0	4
Crocus dolatyarii Rukšāns	Iridaceae	EN	2	0	8
Crocus gilanicus Mathew	Iridaceae	EN	5	5045	20
Crocus gunae Rukšāns	Iridaceae	CR	1	0	4
Crocus iranicus Rukšāns	Iridaceae	CR	1	0	4
Crocus marandicus Dolatyari & Rukšāns	Iridaceae	CR	1	0	4
Crocus pseudoiranicus Dolatyari & Rukšāns	Iridaceae	CR	1	0	4
Crocus reinhardii Rukšāns	Iridaceae	CR	1	0	4
Crotalaria assadii Zaeifi	Fabaceae	VU	7	10096	28
Crucianella gilanica Trin.	Rubiaceae	LC or NT	56	295026	216
Crucianella platyphylla Ehrend. & Schonb.-Tem.	Rubiaceae	EN	2	0	8
Cuscuta haussknechtii Yunck.	Convolvulaceae	CR	1	0	4

Cyanus ouramanicus Ranjbar & Negaresh	Asteraceae	CR	1	0	4
Cyanus persicus Ranjbar & Negaresh	Asteraceae	CR	1	0	4
Cyanus tabrizianus Ranjbar & Negaresh	Asteraceae	CR	1	0	4
Cyclotrichium depauperatum (Bunge) Manden. & Scheng.	Lamiaceae	EN	5	21895	20
Cyclotrichium haussknechtii (Bunge) Manden. & Scheng.	Lamiaceae	EN	2	0	8
Cyclotrichium straussii (Bornm.) Rech.f.	Lamiaceae	EN	4	12305	16
Cymbalaria bakhtiarica Podlech & Iranshahr	Plantaginaceae	CR	1	0	4
Cynoglossum semnanicum Khatamsaz	Boraginaceae	CR	1	0	4
Cyperus celans Kukkonen	Cyperaceae	CR	1	0	4
Daucus littoralis Smith in Sibth. & Smith	Apiaceae	VU	11	142408	44
Delphinium aquilegifolium (Boiss.) Bornm.	Ranunculaceae	LC or NT	25	29455	92
Delphinium elbursense Rech.f.	Ranunculaceae	LC or NT	25	125922	84
Delphinium jacobsii Iranshahr	Ranunculaceae	EN	2	0	8
Delphinium khorasanicum Sharifnia & Hasanbarani	Ranunculaceae	EN	2	0	8
Delphinium lalesaricum Iranshahr	Ranunculaceae	EN	2	0	8
Delphinium lanigerum Boiss. & Hohen.	Ranunculaceae	VU	15	190080	56
Delphinium saniculifolium Boiss.	Ranunculaceae	LC or NT	33	506845	132
Delphinium tuberosum Auch. ex Boiss.	Ranunculaceae	LC or NT	29	498930	116
Delphinium ursinum Rech.f.	Ranunculaceae	VU	14	40109	48
Demavendia pastinacifolia (Boiss. & Hausskn.) Pimenov	Apiaceae	LC or NT	17	539970	68
Deyeuxia kermanensis Assadi	Poaceae	CR	1	0	4
Deyeuxia parsana Bor	Poaceae	EN	4	149	16
Dianthus agrostolepis Rech.f.	Caryophyllaceae	EN	4	3957	16
Dianthus austroiranicus Lemperg in Rech.f.	Caryophyllaceae	VU	10	103997	40
Dianthus binaludensis Rech.f.	Caryophyllaceae	EN	2	0	8
Dianthus denaicus Assadi	Caryophyllaceae	CR	1	0	4
Dianthus diversifolius Assadi	Caryophyllaceae	CR	1	0	4
Dianthus elymaiticus hausskn. & Bornm.	Caryophyllaceae	CR	1	0	4
Dianthus hafezii Assadi	Caryophyllaceae	EN	2	0	8
Dianthus hyrcanicus Rech.f.	Caryophyllaceae	EN	4	3565	16

Dianthus macranthoides Hausskn. ex Bornm.	Caryophyllaceae	LC or NT	34	364180	132
Dianthus mazanderanicus Rech.f.	Caryophyllaceae	EN	2	0	8
Dianthus pseudocrinitus Behroozian & Joharchi	Caryophyllaceae	CR	1	0	4
Dianthus rudbaricus Assadi	Caryophyllaceae	CR	2	0	4
Dianthus sahandicus Assadi	Caryophyllaceae	CR	1	0	4
Dianthus seidlitzii Boiss	Caryophyllaceae	CR	5	16	16
Dianthus stapfii Lemperg in Rech.f.	Caryophyllaceae	VU	6	36083	24
Dianthus stenocephalus Boiss.	Caryophyllaceae	EN	5	8575	20
Dianthus subaphyllus (Lemperg) Rech.f.	Caryophyllaceae	LC or NT	15	114140	60
Dianthus szowitsianus Boiss.	Caryophyllaceae	LC or NT	31	175204	112
Dianthus tabrisianus Bienert ex Boiss.	Caryophyllaceae	VU	10	87034	40
Diaphanoptera gharehbilensis Fadaie	Caryophyllaceae	CR	1	0	4
Diaphanoptera khorasanica Rech.f.	Caryophyllaceae	EN	2	0	8
Diaphanoptera stenocalycina Rech.f. & Schiman-Czeika	Caryophyllaceae	EN	3	23	12
Diceratella canescens (Boiss.) Boiss.	Brassicaceae	LC or NT	14	130573	56
Dichodon tenuifolium Poursakhi, Assadi & F.Ghahrem.	Caryophyllaceae	CR	1	0	4
Dichoropetalum viarium Lyskov & Kljuykov	Apiaceae	CR	1	0	4
Dicyclophora persica Boiss.	Apiaceae	LC or NT	66	326455	260
Didymophysa aucheri Boiss.	Brassicaceae	LC or NT	27	589898	100
Dielsiocharis kotschyi (Boiss.) O. E. Schulz.	Brassicaceae	LC or NT	18	576306	72
Dionysia × kowsariana Zeraatkar & Khajoei	Primulaceae	CR	1	0	4
Dionysia alipourii Lidén & Mehregan	Primulaceae	DD	NA	NA	NA
Dionysia archibaldii Wendelbo	Primulaceae	EN	4	71	16
Dionysia aretioides (Lehm.)Boiss.	Primulaceae	LC or NT	16	9254	60
Dionysia assadii Borjian	Primulaceae	CR	1	0	4
Dionysia aubrietioides Jamzad & Mozaffarian	Primulaceae	EN	2	0	8
Dionysia avia Mehregan, Younesi & Lidén	Primulaceae	CR	1	0	4
Dionysia bazoftica Jamzad	Primulaceae	CR	1	0	4
Dionysia bokharii Lidén & Mehregan	Primulaceae	DD	NA	NA	NA
Dionysia brinkii Lidén, Mauser, A.Ghaderi, Weststr. & Mehregan	Primulaceae	DD	NA	NA	NA

Dionysia bryoides Boiss.	Primulaceae	LC or NT	18	35158	64
Dionysia caespitosa (Duby) Boiss.	Primulaceae	VU	12	28806	48
Dionysia crassifolia Mehregan & Lidén	Primulaceae	DD	NA	NA	NA
Dionysia cristagalli Lidén	Primulaceae	EN	2	0	8
Dionysia curviflora Bunge	Primulaceae	CR	3	8	8
Dionysia diapensiifolia Boiss.	Primulaceae	LC or NT	14	19769	56
Dionysia esfandiarii Wendelbo	Primulaceae	CR	2	0	4
Dionysia gaubae Bornm.	Primulaceae	EN	2	0	8
Dionysia haussknechtii Bornm. & Strauss	Primulaceae	VU	12	19656	48
Dionysia iranica Jamzad	Primulaceae	CR	1	0	4
Dionysia iranshahrii Wendelbo	Primulaceae	EN	2	0	4
Dionysia jamzadiae Lidén, M.Irvine, Alvén & Mehregan	Primulaceae	EN	2	0	8
Dionysia janthina Bornm. & Winkler	Primulaceae	CR	1	0	4
Dionysia juniperina Mauser, Mehregan & Lidén	Primulaceae	DD	NA	NA	NA
Dionysia khatamii Mozaff.	Primulaceae	CR	1	0	4
Dionysia khuzistanica Jamzad	Primulaceae	CR	1	0	4
Dionysia lamingtonii Stapf	Primulaceae	EN	2	0	8
Dionysia leucotricha Bornm.	Primulaceae	LC or NT	26	16567	100
Dionysia lurorum Wendelbo	Primulaceae	CR	1	0	4
Dionysia mallos Mehregan, Weststr. & Lidén	Primulaceae	DD	NA	NA	NA
Dionysia mariae Lidén & Mehregan	Primulaceae	DD	NA	NA	NA
Dionysia michauxii (Duby)Boiss.	Primulaceae	EN	2	0	4
Dionysia moazzenii Lidén & Mehregan	Primulaceae	DD	NA	NA	NA
Dionysia mozaffarianii Liden	Primulaceae	CR	1	0	4
Dionysia oreodoxa Bornm.	Primulaceae	EN	3	597	12
Dionysia revoluta Boiss.	Primulaceae	LC or NT	25	202135	100
Dionysia rhaptodes Bunge	Primulaceae	EN	4	5101	16
Dionysia robusta Younesi	Primulaceae	CR	1	0	4
Dionysia sarvestanica Jamzad & Grey-Wilson	Primulaceae	EN	3	630	12
Dionysia sawyeri (Watt)Wendelbo	Primulaceae	EN	5	1979	20

Dionysia splendens Alipour, Mehregan & Lidén	Primulaceae	CR	1	0	4
Dionysia tacamahaca Lidén	Primulaceae	CR	1	0	4
Dionysia termeana Wendelbo	Primulaceae	EN	4	121	12
Dionysia tjeerdsmae Lidén & Mehregan	Primulaceae	DD	NA	NA	NA
Dionysia viva Lidén & Zetterlund	Primulaceae	CR	1	0	4
Dionysia zagrica Grey-Wilson	Primulaceae	EN	5	1056	20
Dionysia zetterlundii Lidén	Primulaceae	EN	2	0	8
Dionysia zschummelii Lidén	Primulaceae	CR	1	0	4
Diplotaenia cachrydifolia Boiss.	Apiaceae	LC or NT	26	13151	100
Diplotaenia damavandica Mozaff.	Apiaceae	VU	12	1659	44
Dolichorrhiza persica (Boiss.) B. Nord.	Asteraceae	EN	3	5706	12
Dorema ammoniacum D. Don	Apiaceae	LC or NT	26	445235	104
Dorema aucheri Boiss.	Apiaceae	LC or NT	24	422153	96
Doronicum bracteatum Edmondson	Asteraceae	EN	5	2308	20
Doronicum wendelboi Edmondson	Asteraceae	EN	4	4531	16
Dracocephalum aucheri Boiss.	Lamiaceae	LC or NT	50	60140	164
Dracocephalum ghahremanii Jamzad	Lamiaceae	EN	3	11	8
Dracocephalum kotschyi Boiss.	Lamiaceae	LC or NT	157	562007	584
Dracocephalum surmandinum Rech.f.	Lamiaceae	EN	4	1928	16
Drymocallis damghanensis Naderi & Faghir	Rosaceae	EN	2	0	8
Ducrosia assadii Alava	Apiaceae	VU	8	8058	32
Echinophora cinerea (Boiss.) Hedge & Lamond	Apiaceae	LC or NT	15	78572	60
Echinophora platyloba DC.	Apiaceae	LC or NT	53	647578	212
Echinops abazariae Mozaff.	Asteraceae	EN	3	203	12
Echinops arachniolepis Rech.f.	Asteraceae	CR	2	0	4
Echinops aucheri Boiss.	Asteraceae	VU	10	73566	40
Echinops austro-iranicus Mozaff.	Asteraceae	LC or NT	14	17888	56
Echinops avajensis Mozaff.	Asteraceae	VU	6	8230	24
Echinops bakhtiaricus Rech.f.	Asteraceae	VU	9	18092	36
Echinops barezicus Montaz. & Mozaff.	Asteraceae	CR	1	0	4

Echinops barezicus Montazer. & Mozaff.	Asteraceae	LC or NT	7	22502	16
Echinops cephalotes DC.	Asteraceae	LC or NT	39	181896	152
Echinops ceratophorus Boiss.	Asteraceae	LC or NT	17	90383	68
Echinops cervicornis Bornm.	Asteraceae	VU	7	12724	28
Echinops chorassanicus Bunge	Asteraceae	LC or NT	43	160403	172
Echinops cyanocephalus Boiss.	Asteraceae	EN	2	0	8
Echinops delicatus Mozaff.	Asteraceae	CR	1	0	4
Echinops dichrous Boiss. & Hausskh.	Asteraceae	LC or NT	12	56871	44
Echinops disfulensis Bornm.	Asteraceae	CR	1	0	4
Echinops ecbatanus Bornm.	Asteraceae	LC or NT	23	102561	92
Echinops elbursensis Rech.f.	Asteraceae	VU	13	3236	48
Echinops elymaiticus Bornm.	Asteraceae	LC or NT	19	52843	76
Echinops endotrichus Rech.f.	Asteraceae	VU	7	8742	28
Echinops erioceras Bornm.	Asteraceae	EN	6	9510	24
Echinops eriophorus Bornm.	Asteraceae	CR	1	0	4
Echinops farsicus Rech.f.	Asteraceae	LC or NT	12	37451	48
Echinops gedrosiacus Bornm.	Asteraceae	DD	NA	NA	NA
Echinops glanduloso-punctatus Rech.f.	Asteraceae	EN	2	0	8
Echinops haussknechtii Boiss.	Asteraceae	LC or NT	12	95769	48
Echinops hebelepis DC.	Asteraceae	EN	2	0	8
Echinops heteromorphus Bunge	Asteraceae	VU	8	6445	32
Echinops iranshahrii Rech.f.	Asteraceae	VU	7	2137	28
Echinops jesdianus Boiss.	Asteraceae	EN	6	5569	24
Echinops kazerunensis Mozaff.	Asteraceae	CR	2	0	4
Echinops keredjensis Rech.f.	Asteraceae	LC or NT	19	16697	68
Echinops kermanshahanicus Mozaff.	Asteraceae	VU	11	24099	44
Echinops khansaricus Mozaff.	Asteraceae	VU	8	1641	28
Echinops khuzistanicus Mozaff.	Asteraceae	EN	4	1346	16
Echinops koelzii Rech.f.	Asteraceae	LC or NT	21	153178	80
Echinops kotschyi Boiss.	Asteraceae	EN	6	1360	24

Echinops kurdicus Boiss. & Hausskh.	Asteraceae	CR	1	0	4
Echinops lalesarensis Bornm.	Asteraceae	VU	6	76652	24
Echinops laricus Mozaff.	Asteraceae	EN	2	0	8
Echinops lasiolepis Bunge	Asteraceae	VU	7	8858	28
Echinops leiopolyceroides Mozaff.	Asteraceae	LC or NT	13	76216	52
Echinops longipenicillatus Mozaff. & Ghahr.	Asteraceae	LC or NT	15	148887	60
Echinops macrophyllus Boiss. & Hausskn.	Asteraceae	LC or NT	56	235471	212
Echinops mosulensis Rech.f.	Asteraceae	LC or NT	49	272404	184
Echinops nizvanus Rech.f.	Asteraceae	VU	7	9722	28
Echinops pabotii Rech.f.	Asteraceae	CR	1	0	4
Echinops pachyphyllus Rech.f.	Asteraceae	VU	6	1406	24
Echinops persepolitanus Rech.f.	Asteraceae	VU	10	14614	40
Echinops polychromus Rech.f.	Asteraceae	VU	7	2902	24
Echinops procerus Mozaff.	Asteraceae	EN	3	20	8
Echinops psammophilus Mozaff.	Asteraceae	VU	7	6122	28
Echinops quercetorum Mozaff.	Asteraceae	CR	1	0	4
Echinops robustus Bunge	Asteraceae	LC or NT	16	356022	64
Echinops sabzevarensis Mozaff.	Asteraceae	EN	5	81997	20
Echinops shahrudensis Mozaff. & Ghahreman	Asteraceae	CR	2	0	4
Echinops shulabadensis Mozaff.	Asteraceae	EN	4	9417	16
Echinops sojakii Rech . F.	Asteraceae	VU	8	11709	32
Echinops taftanicus Mozaff.	Asteraceae	CR	1	0	4
Echinops viscidulus Mozaff.	Asteraceae	LC or NT	13	5559	52
Echium khuzistanicum Mozaffarian	Boraginaceae	CR	1	0	4
Elaeosticta nodosa (Boiss.) Boiss.	Apiaceae	LC or NT	32	122202	128
Elburzia fenestrata (Boiss.) Hedge	Brassicaceae	VU	10	4493	36
Elymus zagricus Assadi	Poaceae	CR	1	0	4
Eminium jaegeri Bogner & P. C. Boyce	Araceae	CR	1	0	4
Ephedra laristanica Assadi	Ephedraceae	EN	4	10892	16
Eranthis kurdica Rukšāns	Ranunculaceae	EN	3	7227	12

Eremostachys lanata Jamzad	Lamiaceae	EN	2	0	8
Ergocarpon cryptanthum (Rech.f.) C. C. Townsend	Apiaceae	LC or NT	16	58150	64
Erigeron hyrcanicus Bornm. & Vierh.	Asteraceae	VU	10	14898	40
Eriocycla ghafooriana Akhani	Apiaceae	EN	3	2560	12
Eriocycla olivieri (Boiss.) Wolff	Apiaceae	LC or NT	24	14461	92
Eritrichium gracillimum Rech.f.	Boraginaceae	EN	2	0	8
Erodium dimorphum Wendelbo	Geraniaceae	EN	3	12	12
Erodium ghahremanii Assadi & Fakhr-Ranjbari	Geraniaceae	EN	2	0	8
Eryngium iranicum Mozaff.	Apiaceae	EN	1	0	4
Erysimum damirliense Moazzeni & Mahmoudi	Brassicaceae	EN	7	448	16
Erysimum elbrusense Boiss.	Brassicaceae	LC or NT	35	493478	116
Erysimum elymaiticum Mozaffarian	Brassicaceae	EN	2	0	8
Erysimum frigidum Boiss. & Hausskn.	Brassicaceae	EN	2	0	4
Erysimum gelidum Bunge	Brassicaceae	EN	5	121	12
Erysimum hezarense Moazzeni	Brassicaceae	EN	3	840	12
Erysimum koelzii Polatschek & Rech.f.	Brassicaceae	EN	2	0	8
Erysimum nanum Boiss. & Hohen. in Boiss.	Brassicaceae	EN	3	15722	12
Erysimum nasturtioides Boiss. & Hausskn.	Brassicaceae	EN	6	22804	24
Erysimum polatschekii Moazzeni, Assadi & Al-Shehbaz	Brassicaceae	CR	1	0	4
Euphorbia acanthodes Akhani	Euphorbiaceae	CR	1	0	4
Euphorbia aellenii Rech.f.	Euphorbiaceae	EN	2	0	8
Euphorbia austro-iranica Pahlevani	Euphorbiaceae	VU	6	65668	24
Euphorbia belgheisi Pahlevani	Euphorbiaceae	CR	1	0	4
Euphorbia chamanbidensis Nasseh	Euphorbiaceae	CR	1	0	4
Euphorbia connata Boiss. in DC.	Euphorbiaceae	VU	8	43042	32
Euphorbia decipiens Boiss. & Buhse	Euphorbiaceae	LC or NT	28	296022	104
Euphorbia elwendica Stapf	Euphorbiaceae	CR	1	0	4
Euphorbia erythradenia Boiss.	Euphorbiaceae	VU	9	134471	36
Euphorbia ferdowsiana Pahlevani	Euphorbiaceae	CR	1	0	4
Euphorbia gorenflotii Mobayen	Euphorbiaceae	CR	1	0	4

Euphorbia gypsicola Rech.f. & Aell.	Euphorbiaceae	CR	1	0	4
Euphorbia halophila Bronm. & Gauba	Euphorbiaceae	CR	2	0	4
Euphorbia iranshahri Pahlevani	Euphorbiaceae	VU	9	8692	36
Euphorbia kavirensis Pahlevani	Euphorbiaceae	EN	6	28749	24
Euphorbia khabrica Pahlevani	Euphorbiaceae	CR	1	0	4
Euphorbia khorasanica Saeidi & Ghayorm.	Euphorbiaceae	CR	1	0	4
Euphorbia malleata Boiss. in DC.	Euphorbiaceae	EN	2	0	8
Euphorbia mazandaranica Pahlevani	Euphorbiaceae	EN	5	1485	20
Euphorbia mirzakhaniana Pahlevani	Euphorbiaceae	EN	5	2096	16
Euphorbia plebeia Boiss.	Euphorbiaceae	EN	4	8374	16
Euphorbia sahendi Bornm.	Euphorbiaceae	EN	9	20	20
Euphorbia spartiformis Mobayen	Euphorbiaceae	CR	1	0	4
Euphorbia sulphurea Pahlevani	Euphorbiaceae	CR	1	0	4
Euphorbia teheranica Boiss. in DC.	Euphorbiaceae	LC or NT	17	75726	68
Farsetia assadii Kavousi	Brassicaceae	CR	1	0	4
Ferula alliacea Boiss.	Apiaceae	EN	4	97	16
Ferula behboudiana (Rech.f. & Esfand.) Chamberlain	Apiaceae	EN	7	2498	28
Ferula flabelliloba Rech.f. & Aell.	Apiaceae	EN	5	936	20
Ferula gabrielii Rech.f.	Apiaceae	EN	4	95152	16
Ferula hezarlalehzarica Ajani	Apiaceae	EN	6	7025	24
Ferula hirtella Boiss.	Apiaceae	LC or NT	18	180290	72
Ferula kashanica Rech.f.	Apiaceae	EN	6	12257	24
Ferula lutensis Rech.f.	Apiaceae	EN	5	134633	20
Ferula macrocolea Boiss.	Apiaceae	LC or NT	17	109700	68
Ferula microcolea (Boiss.) Boiss.	Apiaceae	LC or NT	23	231837	92
Ferula persica Willd.	Apiaceae	LC or NT	15	12288	60
Ferula pseudalliacea Rech.f.	Apiaceae	VU	11	28174	44
Ferula serpentinica Rech.f.	Apiaceae	EN	4	247	12
Ferula sharifii Rech.f. & Esfand.	Apiaceae	EN	3	84313	12
Ferula stenocarpa Boiss. & Hausskn. in Boiss.	Apiaceae	LC or NT	33	181015	132

Ferula tabasensis Rech.f.	Apiaceae	VU	6	65026	24
Ferula xylorhachis Rech.f.	Apiaceae	VU	9	250366	36
Ferulago carduchorum Boiss. & Haisskn.	Apiaceae	LC or NT	21	486140	84
Ferulago contracta Boiss. & Hausskn.	Apiaceae	VU	10	73725	40
Ferulago phialocarpa Rech.f. & H. Riedl	Apiaceae	EN	5	20228	20
Ferulago trifida Boiss.	Apiaceae	CR	1	0	4
Fessia gilanica Zubov & Rukšāns	Asparagaceae	CR	1	0	4
Festuca akhanii Tzvelev.	Poaceae	CR	1	0	4
Festuca iranica E.B.Alexeev	Poaceae	CR	1	0	4
Festuca rechingeri E.B.Alexeev	Poaceae	CR	1	0	4
Festuca sabalanica E.B.Alexeev	Poaceae	CR	1	0	4
Filago pertomentosa F. Ghahrem. & Akhundzadeh	Asteraceae	EN	3	1367	12
Fortuynia garcinii (Burm.) Shuttlew.	Brassicaceae	LC or NT	38	635056	152
Fritillaria avromanica Advay & Tek?en	Liliaceae	CR	1	0	4
Fritillaria avromanica M. Adway & M. Tekşn	Liliaceae	EN	4	810	16
Fritillaria chlorantha Hausskn. & Bornm.	Liliaceae	VU	10	18130	40
Fritillaria kordestanica Advay	Liliaceae	EN	4	3050	16
Fritillaria kotschyana Herbert	Liliaceae	LC or NT	21	100869	80
Fritillaria olivieri Baker	Liliaceae	VU	9	23580	36
Fritillaria selenica Advay	Liliaceae	CR	1	0	4
Fritillaria shehbazii Advay	Liliaceae	CR	1	0	4
Fritillaria straussii Bornm.	Liliaceae	VU	7	55056	28
Fritillaria zagrica Stapf	Liliaceae	LC or NT	42	387760	148
Gagea calcicola Zarrei & Wilkin	Liliaceae	VU	9	9681	32
Gagea iranica Zarrei & Zarre	Liliaceae	VU	8	21096	32
Gagea robusta Zarrei & Wilkin	Liliaceae	CR	1	0	4
Gagea uliginosa Siehe & Pascher	Liliaceae	VU	8	28572	28
Gagea wendelboi Rech.f.	Liliaceae	EN	3	716	12
Gaillonia dezfulensis Naanaie & Assadi	Rubiaceae	CR	1	0	4
Gaillonia eriantha Jaub. & Spach	Rubiaceae	LC or NT	12	53643	48

Galium aucheri Boiss.	Rubiaceae	VU	10	15439	40
Galium decumbens (Ehrend.) Ehrend. & Schonb.-Tem.	Rubiaceae	LC or NT	20	63720	72
Galium delicatulum Boiss. & Hohen.	Rubiaceae	CR	3	12	12
Galium diploprion Boiss. & Hohen. in Boiss.	Rubiaceae	LC or NT	11	52870	44
Galium elbursense Bornm. & Gauba ex Bornm.	Rubiaceae	EN	2	0	8
Galium iranicum Hausskn. ex Bornm.	Rubiaceae	EN	2	0	8
Galium problematicum (Ehrend.) Ehrend. & Schonb.-Tem.	Rubiaceae	EN	6	1356	24
Galium schoenbeck-Temesyae Ehrend.	Rubiaceae	CR	2	0	4
Galium sojakii Ehrend. & Schonb.-Tem.	Rubiaceae	EN	3	850	12
Galium tehranicum Moussavi, Ghahreman & Attar	Rubiaceae	CR	1	0	4
Gelasia attariana E.Hatami, Mirtadz. & Ebrahimi	Asteraceae	CR	1	0	4
Geranium persicum Schönbeck-Temesy	Geraniaceae	LC or NT	17	231749	64
Geum iranicum Khatamsaz	Rosaceae	CR	2	0	4
Girgensohnia imbricata Bunge	Amaranthaceae	VU	7	85277	28
Gladiolus persicus Boiss.	Iridaceae	LC or NT	20	130439	80
Glaucium calycinum Boiss.	Papaveraceae	EN	3	28260	12
Glaucium contortuplicatum Boiss.	Papaveraceae	EN	5	19076	20
Glaucium elegantissimum Mobayen	Papaveraceae	CR	1	0	4
Glaucium golestanicum Gran & Sharifnia	Papaveraceae	CR	1	0	4
Glaucium mathiolifolium Mobayen	Papaveraceae	CR	1	0	4
Glyceria hyrcana Assadi	Poaceae	CR	1	0	4
Graellsia integrifolia (Rech.f.) Rech.f.	Brassicaceae	VU	7	6445	28
Graellsia stylosa (Boiss. & Hohen.) Poulter	Brassicaceae	LC or NT	23	2230	80
Grantia arachnoidea Boiss.	Asteraceae	EN	4	6171	16
Grantia discoidea Bunge ex Boiss.	Asteraceae	EN	3	1394	12
Gundelia microcephala (Bornm.) Vitek	Asteraceae	DD	NA	NA	NA
Gundelia rosea Al-Taey & Hossain	Asteraceae	DD	NA	NA	NA
Gundelia tehranica Vitek & Noroozi	Asteraceae	EN	6	20	8
Gypsophila acantholimoides Bornm.	Caryophyllaceae	EN	4	5865	16
Gypsophila adenophora Boiss. & Buhse	Caryophyllaceae	CR	1	0	4

Gypsophila alvandica Falat., F.Ghahrem. & Assadi	Caryophyllaceae	CR	1	0	4
Gypsophila bazorganica Rech.f.	Caryophyllaceae	CR	1	0	4
Gypsophila caricifolia Boiss.	Caryophyllaceae	LC or NT	18	102239	72
Gypsophila elymaitica Mozaffarian	Caryophyllaceae	EN	3	1094	12
Gypsophila iranica Barkoudah	Caryophyllaceae	EN	3	12880	12
Gypsophila leioclada Rech.f.	Caryophyllaceae	EN	3	342	8
Gypsophila lurorum Rech.f.	Caryophyllaceae	EN	5	7182	20
Gypsophila melampoda Bienert ex Boiss.	Caryophyllaceae	EN	2	0	8
Gypsophila mucronifolia Rech.f.	Caryophyllaceae	VU	9	1022	36
Gypsophila persica Barkoudah	Caryophyllaceae	LC or NT	21	205707	84
Gypsophila platyphylla Boiss.	Caryophyllaceae	EN	4	32606	16
Gypsophila polyclada Fenzl ex Boiss.	Caryophyllaceae	EN	3	91	12
Gypsophila pseudomelampoda Gauba & Rech.f. in Rech.f.	Caryophyllaceae	VU	7	57442	28
Gypsophila pseudopallida Falat., Assadi & F.Ghahrem.	Caryophyllaceae	CR	1	0	4
Gypsophila rupestris Mozaffarian	Caryophyllaceae	EN	2	0	8
Gypsophila saponarioides Bornm. & Gauba	Caryophyllaceae	EN	3	476	12
Gypsophila wilhelminae Rech.f.	Caryophyllaceae	CR	1	0	4
Gypsophila xanthochlora Rech.f.	Caryophyllaceae	LC or NT	15	11907	60
Gypsophila yazdiana Falat., F.Ghahrem. & Assadi	Caryophyllaceae	CR	1	0	4
Halanthium alaeflavum Assadi	Amaranthaceae	EN	2	0	8
Halimocnemis azarbaijanensis Assadi	Amaranthaceae	EN	2	0	8
Halimocnemis mamamensis (Bge.) Assadi	Amaranthaceae	LC or NT	14	58108	52
Halotis pedunculata Assadi	Amaranthaceae	EN	3	14560	12
Haplophyllum bakhteganicum Soltani & Khosravi	Rutaceae	EN	5	6329	20
Haplophyllum buhsei Boiss.	Rutaceae	EN	2	0	8
Haplophyllum canaliculatum Boiss.	Rutaceae	LC or NT	27	83977	104
Haplophyllum dasygynum C. Townsend	Rutaceae	EN	2	0	8
Haplophyllum furfuraceum Bunge ex Boiss.	Rutaceae	LC or NT	33	67001	132
Haplophyllum glaberrimum Bunge ex Boiss.	Rutaceae	LC or NT	27	426729	104
Haplophyllum laeviusculum C.C.Townsend	Rutaceae	VU	6	7507	24

Haplophyllum laristanicum C.C.Townsend	Rutaceae	CR	1	0	4
Haplophyllum lissonotum C.C.Townsend	Rutaceae	VU	10	33750	40
Haplophyllum rechingeri C. Townsend	Rutaceae	EN	3	4100	12
Haplophyllum rubro-tinctum C. Townsend	Rutaceae	LC or NT	15	40719	60
Haplophyllum stapfianum Hand.-Mzt.	Rutaceae	EN	5	4264	20
Haplophyllum virgatum Spach	Rutaceae	VU	7	27771	28
Haplophyllum viridulum Sojak	Rutaceae	EN	6	3732	24
Haussknechtia elymaitica Boiss.	Apiaceae	EN	4	4914	16
Hedysarum alamutense H. Nafsi & Kaz. Osaloo	Fabaceae	EN	3	359	12
Hedysarum al-shehbazii Ranjbar	Fabaceae	CR	1	0	4
Hedysarum bojnordense Ranjbar & Joharchi	Fabaceae	EN	3	598	12
Hedysarum callithrix Bunge ex Boiss.	Fabaceae	EN	3	145393	12
Hedysarum criniferum Boiss.	Fabaceae	LC or NT	28	137140	112
Hedysarum damghanicum Rech.f.	Fabaceae	CR	2	0	4
Hedysarum elbursense Bornm. & Gauba	Fabaceae	CR	1	0	4
Hedysarum glabrifoliolatum Ranjbar	Fabaceae	CR	1	0	4
Hedysarum gypsophilum Dehshiri	Fabaceae	CR	1	0	4
Hedysarum halophilum Bornm. & Gauba	Fabaceae	CR	1	0	4
Hedysarum hyrcanum Bornm. & Gauba	Fabaceae	EN	2	0	4
Hedysarum johartchii Ranjbar	Fabaceae	EN	3	69	8
Hedysarum kalatense Dehshiri	Fabaceae	CR	1	0	4
Hedysarum longipedunculatum Ranjbar & Joharchi	Fabaceae	CR	1	0	4
Hedysarum marandense Mozaffarian	Fabaceae	CR	1	0	4
Hedysarum neyshaboricum Ranjbar	Fabaceae	CR	1	0	4
Hedysarum orumiehense Ranjbar	Fabaceae	CR	1	0	4
Hedysarum papillosum Boiss.	Fabaceae	EN	2	0	8
Hedysarum paucifoliolatum Ranjbar & Joharchi	Fabaceae	CR	1	0	4
Hedysarum persicum Bidarlord, F.Ghahrem. & Mozaff.	Fabaceae	CR	1	0	4
Hedysarum plumosum Boiss. & Hausskn.	Fabaceae	CR	1	0	4
Hedysarum pseudomacranthum Assadi, Chagham. & F.Ghahrem.	Fabaceae	CR	1	0	4

Hedysarum renzii Rech.f.	Fabaceae	CR	1	0	4
Heldreichia longifolia Boiss.	Brassicaceae	EN	2	0	8
Helianthemum assadii Ghahremaninejad & Gholamian	Cistaceae	VU	6	100676	24
Helianthemum sinuspersicum Gholamian & Ghahremaninejad	Cistaceae	EN	2	0	8
Helichrysum artemisioides Boiis. & Hausskn.	Asteraceae	VU	8	34512	32
Helichrysum athanaton Georgiadou & Rech.f.	Asteraceae	EN	5	48958	20
Helichrysum davisianum Rech.f.	Asteraceae	EN	4	25	16
Helichrysum globiferum Boiss.	Asteraceae	LC or NT	32	298726	128
Helichrysum kermanicum Mozaffarian & Rajaei	Asteraceae	CR	1	0	4
Helichrysum leucocephalum Boiss.	Asteraceae	LC or NT	29	348813	116
Helichrysum makranicum (Rech.f. & Esfand.) Rech.f.	Asteraceae	EN	2	0	8
Helichrysum oligocephalum DC.	Asteraceae	LC or NT	58	317521	224
Helichrysum persicum F.Ghahrem. & Noori	Asteraceae	CR	1	0	4
Heliocarya monandra Bunge	Boraginaceae	EN	4	526	16
Heliotropium agdense Bunge	Boraginaceae	VU	10	271951	40
Heliotropium aucheri	Boraginaceae	LC or NT	35	96360	136
Heliotropium denticulatum Boiss. et Hausskn. in Boiss.	Boraginaceae	EN	4	16021	16
Heliotropium disciforme Akhani	Boraginaceae	EN	5	2550	20
Heliotropium esfahanicum Khatamsaz	Boraginaceae	CR	1	0	4
Heliotropium esfandiarii Akhani & Riedl	Boraginaceae	LC or NT	16	11782	60
Heliotropium gypsaceum Rech.f.	Boraginaceae	EN	3	740	12
Heliotropium kaserunense Bronm.	Boraginaceae	CR	1	0	4
Heliotropium khayyamii Akhani	Boraginaceae	CR	1	0	4
Heliotropium samoliflorum Bunge	Boraginaceae	LC or NT	26	162201	104
Heliotropium shirazicum Mozaffarian	Boraginaceae	CR	1	0	4
Heliotropium ziegleri Akhani	Boraginaceae	EN	6	178	24
Heracleum anisactis Boiss. & Hohen.	Apiaceae	VU	10	18986	40
Heracleum gorganicum Rech.f.	Apiaceae	EN	5	11696	20
Heracleum kurdistanicum Rastegar, Maroofi & Tabad	Apiaceae	CR	1	0	4
Heracleum rawianum C. C. Towns.	Apiaceae	EN	6	37109	20

Heracleum rechingeri Manden.	Apiaceae	EN	5	1953	20
Hertia angustifolia (DC.) O. Kuntze	Asteraceae	LC or NT	43	312041	172
Hesperis bakhtiarica Eslami-Farouji, Assadi & Khodayari	Brassicaceae	DD	NA	NA	NA
Hesperis borbasii Dvorák	Brassicaceae	CR	1	0	4
Hesperis ilamica Eslami-Farouji, Khodayari & Assadi	Brassicaceae	EN	4	1017	16
Hesperis leucoclada Boiss.	Brassicaceae	VU	7	94139	28
Hesperis luristanica Dvo?ák	Brassicaceae	EN	2	0	8
Hesperis nivalis Boiss. & Hausskn.	Brassicaceae	LC or NT	15	170157	60
Heteroderis pusilla Boiss.	Asteraceae	DD	NA	NA	NA
Hieracium azerbaijanense Lack	Asteraceae	EN	3	60	8
Hieracium cheirifolium Boiss. & Hausskn.	Asteraceae	EN	5	2104	20
Hieracium piranshahricum Tavakkoli & Assadi	Asteraceae	EN	2	0	8
Hyacinthella persica (Boiss. ex Buhse) Chouard	Asparagaceae	EN	5	37	20
Hymenocephalus rigidus Jaub. & Spech	Asteraceae	EN	2	0	8
Hymenocrater incanus Bunge	Lamiaceae	LC or NT	26	133589	100
Hymenocrater oxyodontus Rech.f.	Lamiaceae	VU	6	70218	24
Hymenocrater platystegius Rech.f.	Lamiaceae	LC or NT	16	60052	64
Hymenocrater yazdianus Rech.f.	Lamiaceae	EN	7	33008	28
Hyoscyamus bornmulleri Khatamsaz	Solanaceae	CR	1	0	4
Hyoscyamus kotschyanus pojark.	Solanaceae	LC or NT	17	154413	64
Hyoscyamus kurdicus Bornm.	Solanaceae	VU	6	175454	24
Hyoscyamus malekianus Parsa	Solanaceae	EN	8	59	24
Hyoscyamus tenuicaulis Schonbeck-Temesy	Solanaceae	LC or NT	34	335873	136
Hypericopsis persica Boiss.	Frankeniaceae	EN	7	3224	28
Hypericum dogonbadanicum Assadi	Hypericaceae	EN	6	20	20
Hypericum fursei N.Robson	Hypericaceae	CR	1	0	4
Indigofera sinuspersica Mozaffarian	Fabaceae	CR	1	0	4
Inula persica F.Ghahrem. & Narimisa	Asteraceae	CR	1	0	4
Inula rajamandii Narimisa & F.Ghahrem.	Asteraceae	CR	1	0	4
Iranecio elbrusensis (Boiss.) B. Nord	Asteraceae	LC or NT	18	25114	68

Iranecio oligolepis (Boiss.) B. Nord.	Asteraceae	EN	4	46	12
Iris barnumiae Baker & Foster	Iridaceae	VU	15	4978	56
Iris ferdowsii Joharchi & Memariani	Iridaceae	VU	9	1633	36
Iris meda Stapf	Iridaceae	LC or NT	18	140729	72
Iris pseudomeda Salimb. & H.Saeidi	Iridaceae	DD	NA	NA	NA
Isatis campylocarpa Boiss.	Brassicaceae	LC or NT	19	55374	72
Isatis gaubae Bornm.	Brassicaceae	LC or NT	20	30656	80
Isatis koeiei Rech.f.	Brassicaceae	LC or NT	27	701009	104
Isatis pachycarpa Rech.f.	Brassicaceae	EN	2	0	8
Isatis raphanifolia Boiss.	Brassicaceae	LC or NT	37	426365	148
Isatis rugulosa Bunge ex Boiss.	Brassicaceae	VU	9	59497	36
Isatis zarrei Al-Shehbaz	Brassicaceae	VU	8	444	32
Johrenia golestanica Rech.f.	Apiaceae	EN	3	8	8
Johrenia ramosissima Mozaff.	Apiaceae	EN	4	365	16
Johreniopsis oligactis (Rech.f. & H.Riedl.) M. Pimen.	Apiaceae	CR	1	0	4
Johreniopsis scoparia (Boiss.) Pimenov	Apiaceae	LC or NT	19	115344	76
Johreniopsis stricticaulis (Rech.f.) M. Pimen.	Apiaceae	EN	5	4363	20
Jurinea boreoiranica Mirtadz. & Naderi	Asteraceae	EN	8	149	20
Jurinea bungei Boiss.	Asteraceae	VU	6	23808	24
Jurinea cartilaginea Mozaff.	Asteraceae	CR	1	0	4
Jurinea catharinae Iljin.	Asteraceae	VU	6	11538	24
Jurinea cordata Boiss. & Hausskn.	Asteraceae	EN	2	0	8
Jurinea eriobasis DC.	Asteraceae	VU	9	43351	36
Jurinea gabrielae Bornm.	Asteraceae	CR	1	0	4
Jurinea gedrosiaca Bornm.	Asteraceae	EN	2	0	8
Jurinea giviensis Mirtadz.	Asteraceae	CR	1	0	4
Jurinea heterophylla (Jaub. & Spech) Borss.	Asteraceae	VU	8	76876	32
Jurinea inuloides Boiss. & hausskn	Asteraceae	CR	1	0	4
Jurinea jedresiaca Bornm.	Asteraceae	CR	1	0	4
Jurinea khorassanica Joharchi & Mirtadzadini	Asteraceae	CR	1	0	4

Jurinea kopetensis Rech.f.	Asteraceae	EN	2	0	8
Jurinea leptoloba DC.	Asteraceae	VU	7	4373	28
Jurinea meda Bornm.	Asteraceae	LC or NT	25	77729	88
Jurinea mobayenii Ghahreman & Mirtadzadini	Asteraceae	CR	1	0	4
Jurinea monocephala Aitch. & Hemsl.	Asteraceae	LC or NT	21	48730	84
Jurinea multicaulis DC.	Asteraceae	EN	3	2271	12
Jurinea prasinophylla Rech.f.	Asteraceae	EN	2	0	8
Jurinea proteoides Boiss & Hausskn.	Asteraceae	CR	1	0	4
Jurinea radians Boiss.	Asteraceae	LC or NT	20	549207	80
Jurinea sharifiana Rech.f.	Asteraceae	EN	2	0	8
Jurinea stenocalathia Rech.f.	Asteraceae	VU	6	137973	24
Jurinea stenocarpa Mirtadz.	Asteraceae	EN	4	241	12
Jurinea viciosoi Pau	Asteraceae	EN	2	0	8
Jurinella frigida (Boiss.) Wagenitz	Asteraceae	LC or NT	42	32784	144
Jurinella microcephala (Boiss.) Wagenitz	Asteraceae	LC or NT	21	70107	80
Kalakia marginata (Boiss.) Alava	Apiaceae	LC or NT	19	33047	72
Kaviria zehzadii (Akhani) Akhani	Amaranthaceae	CR	3	8	8
Kelussia odoratissima Mozaff.	Apiaceae	EN	2	0	8
Kickxia iranica Zeraatkar & F. Ghahrem.	Plantaginaceae	CR	1	0	4
Klasea nana Ranjbar & Negaresh	Asteraceae	CR	1	0	4
Klasea sanandajensis Ranjbar & Negaresh	Asteraceae	CR	1	0	4
Lactuca azerbaijanica Rech.f.	Asteraceae	EN	4	2056	12
Lactuca birjandica Mozaff.	Asteraceae	CR	1	0	4
Lactuca denaensis N. Kilian & Djavadi	Asteraceae	CR	1	0	4
Lactuca gilanica Mozaff.	Asteraceae	CR	1	0	4
Lactuca hazaranensis Djavadi & N. Kilian	Asteraceae	CR	1	0	4
Lactuca polyclada Boiss.	Asteraceae	EN	2	0	8
Lagochilus alutaceus Bunge	Lamiaceae	LC or NT	12	172422	48
Lagochilus aucheri Boiss.	Lamiaceae	LC or NT	138	596065	532
Lagochilus khorassanicus Zeraatkar, F. Ghahrem. & Joharchi	Lamiaceae	LC or NT	23	33262	92

Lagochilus kotschyanus Boiss.	Lamiaceae	LC or NT	27	172236	104
Lagochilus lasiocalyx (Stapf) Jamzad	Lamiaceae	LC or NT	15	53698	60
Lagochilus lorestanicus Dehshiri & Mozaffarian	Lamiaceae	CR	1	0	4
Lagochilus macracanthus Fisch. & C.A. Mey. in Schrenk	Lamiaceae	LC or NT	18	177775	72
Lagochilus quadridentatus Jamzad	Lamiaceae	CR	1	0	4
Lamium bakhtiaricum Jamzad	Lamiaceae	CR	1	0	4
Laser rechingeri Akhani	Apiaceae	EN	4	39	12
Lathyrus alamutensis Mozaffarian	Fabaceae	CR	1	0	4
Launaea acanthodes (Boiss.) O. Kuntze	Asteraceae	LC or NT	130	870191	512
Launaea bornmuelleri (Hausskn. Ex BORBN.) Bornm.	Asteraceae	CR	1	0	4
Launaea peistocarpa (Boiss.) Rech.f.	Asteraceae	EN	4	7519	16
Lavandula sublepidota Rech.f.	Lamiaceae	EN	3	243	12
Leontodon stenocalathius Rech.f.	Asteraceae	EN	3	266	12
Leopoldia tabriziana Jafari	Asparagaceae	EN	2	0	8
Lepechiniella fursei H. Riedl	Boraginaceae	CR	1	0	4
Lepechiniella persica (Boiss.) H. Riedl	Boraginaceae	EN	5	10237	20
Lepechiniella wendelboi H. Riedl in Wendelbo	Boraginaceae	VU	7	20317	28
Lepidium khalkhalicum Bidarlord & D.A.German	Brassicaceae	EN	2	0	8
Lepidium pabotii Al-Shahbaz	Brassicaceae	CR	1	0	4
Leucopoa pseudosclerophylla (Krivot.) Bor	Poaceae	EN	4	3032	16
Leutea avicennae Mozaff. Bot. Zhurnal	Apiaceae	CR	1	0	4
Leutea cupularis (Boiss.) M. Pimen.	Apiaceae	LC or NT	15	105416	60
Leutea elbursensis Mozaff. Bot. Zhurnal	Apiaceae	EN	6	566	20
Leutea gracillima M. Pimen.	Apiaceae	EN	3	76	12
Leutea kurdistanica Mozaff. Bot. Zhurnal	Apiaceae	EN	2	0	8
Leutea nematoloba (Rech.f.) M. Pimen.	Apiaceae	EN	5	347	16
Leutea polyscias (Boiss.) M. Pimen.	Apiaceae	EN	6	468	20
Leutea rechingeri (Leute) Pimenov	Apiaceae	EN	5	669	20
Ligularia persica Boiss.	Asteraceae	VU	12	4044	44
Linaria azerbaijanensis Hamdi & Assadi	Plantaginaceae	LC or NT	16	55405	64

Linaria birjandensis Hamdi	Plantaginaceae	EN	2	0	8
Linaria boushehrensis Hamdi & Assadi	Plantaginaceae	EN	5	9044	20
Linaria elymaitica (Boiss.) Kuprian.	Plantaginaceae	LC or NT	23	234701	92
Linaria farsensis S. M. M Hamdi & M. Assadi	Plantaginaceae	EN	4	64650	16
Linaria golestanensis Hamdi & Assadi	Plantaginaceae	EN	7	2411	28
Linaria guilanensis Hamdi & Assadi	Plantaginaceae	EN	4	2068	16
Linaria iranica Hamdi & Assadi	Plantaginaceae	CR	1	0	4
Linaria karajensis Hamdi & Assadi	Plantaginaceae	EN	3	47	12
Linaria kavirensis Hamdi & Assadi	Plantaginaceae	VU	7	375234	28
Linaria khalkhalensis Hamdi & Assadi	Plantaginaceae	EN	3	243	12
Linaria khorasanensis Hamdi & Assadi	Plantaginaceae	VU	7	14824	28
Linaria lineolata Boiss.	Plantaginaceae	LC or NT	56	68396	212
Linaria mazandaranensis Hamdi & Assadi	Plantaginaceae	LC or NT	14	29387	52
Linaria michauxii Chav.	Plantaginaceae	LC or NT	65	880612	256
Linaria nurensis Miller	Plantaginaceae	LC or NT	31	115023	124
Linaria orientalis Hamdi & Assadi	Plantaginaceae	CR	1	0	4
Linaria remotiflora Patzak	Plantaginaceae	VU	10	104620	40
Linaria semnanensis Hamdi & Assadi	Plantaginaceae	EN	3	198	12
Linaria shahroudensis Hamdi & Assadi	Plantaginaceae	EN	5	16	16
Lindelofia kandavanensis Bornm. & Gauba	Boraginaceae	EN	5	465	20
Linum khorassanicum Joharchi & Behroozian	Linaceae	EN	4	246	16
Linum persicum Ky. ex Boiss.	Linaceae	EN	4	4608	16
Lomatopodium staurophyllum (Rech.f.) Rech.f.	Apiaceae	VU	9	38117	36
Malabaila isfahanica Alava	Apiaceae	CR	1	0	4
Malabaila kotschyi Boiss.	Apiaceae	LC or NT	25	571015	96
Malabaila porphyrodiscus Stapf & Wettst.	Apiaceae	LC or NT	33	132897	132
Marrubium eriocephalum Seybold	Lamiaceae	CR	3	8	8
Marrubium procerum Bunge	Lamiaceae	EN	2	0	8
Matthiola dumulosa Boiss. & Buhse	Brassicaceae	LC or NT	14	158895	56
Matthiola iranica Zeraatkar, Mahmoodi, F.Ghahrem. & Maassoumi	Brassicaceae	VU	8	36749	32

Matthiola ovatifolia (Boiss.) Boiss.	Brassicaceae	LC or NT	20	349627	80
Matthiola revoluta Bunge ex Boiss.	Brassicaceae	LC or NT	19	682455	76
Matthiola shehbazii Ranjbar & Karami	Brassicaceae	CR	1	0	4
Matthiola shiraziana Zeraatkar, Khosravi, F. Ghahrem., Al-Shehbaz & Assadi	Brassicaceae	VU	10	10958	36
Matthiola subglabra Ponert	Brassicaceae	CR	1	0	4
Mattiastrum pygmaeum Rech.f.	Boraginaceae	EN	2	0	8
Mentha mozaffarianii Jamzad	Lamiaceae	VU	7	9497	28
Michauxia koeieana Rech.f.	Campanulaceae	EN	3	1018	12
Michauxia stenophylla Boiss. & Hausskn.	Campanulaceae	EN	4	4087	16
Micrantha multicaulis (Boiss.) F. Dvorák	Brassicaceae	LC or NT	11	30413	44
Micromeria hedgei Rech.f.	Lamiaceae	LC or NT	21	78985	80
Milium atropatanum Maroofi	Poaceae	EN	3	338	12
Minuartia acuminata Turrill	Caryophyllaceae	EN	4	11853	12
Minuartia aucheriana (Boiss.) Bornm.	Caryophyllaceae	EN	4	4101	16
Minuartia khorassanica Assadi & Mostafavi	Caryophyllaceae	CR	1	0	4
Minuartia lineata Bornm.	Caryophyllaceae	LC or NT	35	77013	124
Minuartia sabalanica Assadi & Mostafavi	Caryophyllaceae	CR	1	0	4
Moltkia gypsacea Rech.f. & Aellen,	Boraginaceae	DD	NA	NA	NA
Mozaffariania insignis Pimen. & Maassoumi Bot. Zhurn. 87	Apiaceae	EN	5	8528	20
Muscari kurdicum Maroofi	Asparagaceae	CR	1	0	4
Muscari pseudomuscari (Boiss. & Buhse) Wendelbo	Asparagaceae	EN	5	117850	20
Myopordon aucheri Boiss.	Asteraceae	CR	2	0	4
Myopordon damavandica Mozaff.	Asteraceae	EN	2	0	8
Myopordon hyrcanum (Bornm.) Wagenitz	Asteraceae	EN	4	31	16
Myopordon persicum Boiss.	Asteraceae	EN	4	193	16
Myosotis anomala H.Riedl	Boraginaceae	VU	9	19610	36
Myosotis koelzii H.Rield	Boraginaceae	EN	3	113	12
Nannorrhops baluchestanica Khodashenas	Arecaceae	CR	1	0	4
Nanorrhinum baluchestanicum Naanaie	Plantaginaceae	CR	1	0	4

Nanorrhinum campyloceras (Rech.f. & Esfand.) Naanaie	Plantaginaceae	LC or NT	30	158127	116
Nanorrhinum chasmophyticum (Wendelbo) Naanaie	Plantaginaceae	EN	3	46432	12
Nanorrhinum khuzestanicum Naanaie	Plantaginaceae	LC or NT	13	35036	52
Nectaroscordum koelzii Wendelbo	Amaryllidaceae	VU	8	50274	32
Neocryptodiscus persicus (Boiss.) Hedge & Lamond	Apiaceae	EN	6	7617	24
Nepeta adenoclada Bornm.	Lamiaceae	EN	2	0	8
Nepeta allotria Rech.f.	Lamiaceae	EN	2	0	4
Nepeta archibaldii Rech.f.	Lamiaceae	EN	7	44	24
Nepeta assadii Jamzad	Lamiaceae	VU	6	57844	24
Nepeta assurgens Hausskn. & Bornm.	Lamiaceae	VU	11	32473	40
Nepeta asterotricha Rech.f.	Lamiaceae	EN	9	76	36
Nepeta azadkouhensis Saberamoli	Lamiaceae	CR	1	0	4
Nepeta bakhtiarica Rech.f.	Lamiaceae	VU	7	58704	28
Nepeta balouchestanica Jamzad & Ingr.	Lamiaceae	EN	2	0	4
Nepeta Bazoftica Jamzad	Lamiaceae	CR	1	0	4
Nepeta binaloudensis Jamzad	Lamiaceae	VU	11	1087	40
Nepeta bokhonica Jamzad	Lamiaceae	CR	1	0	4
Nepeta bornmuelleri Hausskn. & Bornm.	Lamiaceae	EN	6	4262	24
Nepeta cephalotes Boiss.	Lamiaceae	LC or NT	16	31947	56
Nepeta chionophila Boiss. & Hausskn.	Lamiaceae	EN	6	1143	24
Nepeta crassifolia Boiss. & Buhse	Lamiaceae	LC or NT	56	350783	204
Nepeta crispa Willd.	Lamiaceae	LC or NT	16	62732	56
Nepeta denudata Benth. in DC.	Lamiaceae	LC or NT	35	46394	136
Nepeta depauperata Benth. in DC.	Lamiaceae	LC or NT	18	127963	72
Nepeta dschuparensis Bornm.	Lamiaceae	VU	15	49065	56
Nepeta eremokosmos Rech.f.	Lamiaceae	VU	13	3168	52
Nepeta gloeocephala Rech.f.	Lamiaceae	VU	11	5001	44
Nepeta hormozganica Jamzad	Lamiaceae	VU	8	50292	32
Nepeta hymenodonta Boiss.	Lamiaceae	CR	1	0	4
Nepeta iranshahrii Rech.f.	Lamiaceae	EN	4	203	16

Nepeta iraqo-iranica Haloob, Bordbar & Qader	Lamiaceae	EN	2	0	8
Nepeta koieana Rech.f.	Lamiaceae	CR	1	0	4
Nepeta lasiocephala Benth.	Lamiaceae	VU	12	85080	44
Nepeta laxiflora Benth.	Lamiaceae	LC or NT	48	171006	188
Nepeta mahanensis Jamzad & Simmonds	Lamiaceae	EN	2	0	8
Nepeta makuensis Jamzad	Lamiaceae	EN	3	116	12
Nepeta minuticephala Jamzad	Lamiaceae	EN	2	0	8
Nepeta monocephala Rech.f.	Lamiaceae	EN	4	1196	12
Nepeta natanzensis Jamzad	Lamiaceae	EN	6	54552	20
Nepeta oxyodonta Boiss.	Lamiaceae	LC or NT	66	148216	256
Nepeta pogonosperma Jamzad & Assadi	Lamiaceae	VU	12	5323	40
Nepeta prostrata Benth.	Lamiaceae	LC or NT	17	20013	68
Nepeta racemosa Lam.	Lamiaceae	LC or NT	56	81018	204
Nepeta rivularis Bornm.	Lamiaceae	EN	5	34	16
Nepeta sahandica Noroozi & Ajani	Lamiaceae	EN	4	5928	12
Nepeta schiraziana Boiss.	Lamiaceae	LC or NT	38	415264	148
Nepeta sessilifolia Bunge	Lamiaceae	LC or NT	27	36712	104
Nepeta shahmirzadensis Assadi & Jamzad	Lamiaceae	EN	3	228	12
Nepeta straussii Hausskn. & Bornm.	Lamiaceae	LC or NT	16	35976	60
Noccaea apterocarpa (Rech.f. & Aellen) Al-Shehbaz & Menke	Brassicaceae	VU	10	27883	40
Noccaea tenuis (Boiss. & Buhse) F. K. Mey.	Brassicaceae	EN	5	2817	20
Noccidium tuberculatum F. K. Mey.	Brassicaceae	CR	1	0	4
Nonea anchusoides Boiss. & Buhse	Boraginaceae	LC or NT	11	53716	44
Nonea hypoleia Bornm.	Boraginaceae	EN	2	0	8
Nonea iranica Falatoury & Pakravan	Boraginaceae	VU	7	92863	28
Nonea persica Boiss.	Boraginaceae	LC or NT	62	651027	248
Onobrychis alamutensis Amirah.	Fabaceae	CR	1	0	4
Onobrychis alborzensis Ranjbar & Hajmoradi	Fabaceae	EN	5	2541	20
Onobrychis andalanica Bornm.	Fabaceae	EN	3	2235	12
Onobrychis assadii Ranjbar	Fabaceae	CR	1	0	4

Onobrychis atropatanoides Amirab.	Fabaceae	CR	1	0	4
Onobrychis aucheri Boiss.	Fabaceae	EN	4	35282	16
Onobrychis aurea Ranjbar	Fabaceae	CR	1	0	4
Onobrychis avajensis Ranjbar	Fabaceae	EN	2	0	8
Onobrychis avanakensis Amirahm.	Fabaceae	DD	NA	NA	NA
Onobrychis chaldoranensis Toluei, Ranjbar & Wink	Fabaceae	CR	1	0	4
Onobrychis depauperata Boiss.	Fabaceae	CR	1	0	4
Onobrychis elymaitica Boiss. & Hausskn. in Boiss.	Fabaceae	EN	2	0	8
Onobrychis farimanensis Ranjbar & Askari	Fabaceae	CR	1	0	4
Onobrychis garinensis Dehshiri	Fabaceae	CR	1	0	4
Onobrychis gaubae Bornm.	Fabaceae	LC or NT	21	112412	80
Onobrychis gypsicola Rech.f.	Fabaceae	VU	6	16555	24
Onobrychis heliocarpa Boiss.	Fabaceae	EN	4	8835	16
Onobrychis iranensis Amirabadizadeh & Ghanavati	Fabaceae	VU	10	64096	40
Onobrychis iranshahrii Rech.f.	Fabaceae	EN	2	0	8
Onobrychis kermanensis (Sirj. & Rech.f.) Rech.f.	Fabaceae	EN	2	0	8
Onobrychis longipes Bunge in Boiss.	Fabaceae	CR	1	0	4
Onobrychis luristanica Rech.f.	Fabaceae	EN	4	2349	16
Onobrychis marandensis Amirabadi. & Ghanavati	Fabaceae	EN	3	71	12
Onobrychis mazanderanica Rech.f.	Fabaceae	VU	11	20197	44
Onobrychis melanotricha Boiss.	Fabaceae	LC or NT	52	386356	208
Onobrychis mozaffarianii Amirabadizadeh	Fabaceae	CR	1	0	4
Onobrychis oshnaviyehensis Ranjbar	Fabaceae	EN	2	0	8
Onobrychis oxyptera Boiss.	Fabaceae	EN	6	291	24
Onobrychis persica Sirj. & Rech.f.	Fabaceae	LC or NT	13	120127	52
Onobrychis plantago Bornm.	Fabaceae	EN	3	701	12
Onobrychis psoraleifolia Boiss.	Fabaceae	LC or NT	16	60991	60
Onobrychis ptychophylla Sirj. & Rech.f.	Fabaceae	CR	1	0	4
Onobrychis rechingerorum Wendelbo	Fabaceae	CR	2	0	4
Onobrychis sanandajensis Amirabadizadeh & Ghanavati	Fabaceae	CR	1	0	4

Onobrychis scrobiculata Boiss.	Fabaceae	LC or NT	35	93136	140
Onobrychis semnanensis Širj. & Rech.f.	Fabaceae	DD	NA	NA	NA
Onobrychis sojakii Rech.f.	Fabaceae	EN	2	0	8
Onobrychis subnitens Bornm.	Fabaceae	VU	10	45369	40
Onobrychis susiana Nab.	Fabaceae	EN	2	0	8
Onobrychis sylvatica Amirab.	Fabaceae	DD	NA	NA	NA
Onobrychis szovitsii Boiss.	Fabaceae	EN	2	0	8
Onobrychis talagonica Rech.f.	Fabaceae	EN	4	24	16
Onopordon caramanicum (Bornm.) Bornm.	Asteraceae	LC or NT	13	286331	52
Onosma alburzensis Attar, Amini Rad & Mirtadz.	Boraginaceae	EN	3	1842	12
Onosma assadi Mehrabian & Mozaff.	Boraginaceae	EN	5	578	12
Onosma atrii Mehrabian & Khajoei	Boraginaceae	CR	1	0	4
Onosma azarbaidjanensis Mehrabian	Boraginaceae	CR	1	0	4
Onosma bakhteganensis Mozaff. & Mehrabian	Boraginaceae	EN	3	760	12
Onosma bilabiata Boiss. & Buhse	Boraginaceae	VU	11	50249	44
Onosma bisotunensis Attar & Hamzeh'ee	Boraginaceae	CR	1	0	4
Onosma chrysochaetum Bornm.	Boraginaceae	VU	7	128584	28
Onosma deltiphylla Attar, Mirtadz. & Sotoodeh	Boraginaceae	DD	NA	NA	NA
Onosma demavendica H.Riedl	Boraginaceae	LC or NT	14	84212	56
Onosma fareghanensis Attar, Mirtadz. & Sotoodeh	Boraginaceae	EN	5	19581	20
Onosma ghahremanii Attar & Naqinezhad	Boraginaceae	CR	1	0	4
Onosma humilis Attar, Sotoodeh & Mirtadz.	Boraginaceae	DD	NA	NA	NA
Onosma iranshahrii Ghahreman & Atter	Boraginaceae	CR	1	0	4
Onosma kerendica Attar & Mirtadz.	Boraginaceae	EN	2	0	8
Onosma khatamsazii Attar, Mirtadz. & Sotoodeh	Boraginaceae	VU	8	21683	32
Onosma khorassanica Attar & Joharchi	Boraginaceae	EN	2	0	8
Onosma khorramabadensis Attar, Mirtadz. & Sotoodeh	Boraginaceae	EN	2	0	8
Onosma kilouyense Boiss. & Hausskn	Boraginaceae	LC or NT	44	235488	164
Onosma kotschyi Boiss.	Boraginaceae	LC or NT	37	423111	148
Onosma lorestanica Attar & Sotoodeh	Boraginaceae	EN	2	0	8

Onosma maculata Ranjbar & Almasi	Boraginaceae	CR	1	0	4
Onosma mahabadensis Ranjbar & Almasi	Boraginaceae	EN	2	0	8
Onosma maharluensis Attar, Mirtadz. & Sotoodeh	Boraginaceae	VU	8	10327	24
Onosma marivanensis Mozaff. & Mehrabian	Boraginaceae	CR	1	0	4
Onosma moussavi Mehrabian & Amini Rad	Boraginaceae	CR	1	0	4
Onosma mozaffariani Mehrabian.	Boraginaceae	CR	1	0	4
Onosma pachypoda Boiss.	Boraginaceae	LC or NT	23	213978	88
Onosma persica Mehrabian & Naghizadeh	Boraginaceae	CR	1	0	4
Onosma platyphylla H.Riedl	Boraginaceae	LC or NT	15	52644	60
Onosma sabalanica Ponert	Boraginaceae	EN	5	1438	16
Onosma sahandica Attar & Sotoodeh	Boraginaceae	CR	1	0	4
Onosma sanandajensis Mozaff. & Mehrabian	Boraginaceae	CR	1	0	4
Onosma sarvestanica Mozaff. & Mehrabian	Boraginaceae	EN	2	0	8
Onosma shehbazii Advay, Attar & S.A.Ahmad	Boraginaceae	CR	1	0	4
Onosma sheidaii Mehrabian	Boraginaceae	CR	1	0	4
Onosma stenosiphon	Boraginaceae	LC or NT	29	645996	116
Onosma straussii (Riedl) Khatamsaz	Boraginaceae	LC or NT	16	85683	60
Onosma targevarensis Mozaff. & Mehrabian	Boraginaceae	CR	2	0	4
Onosma wendelboii Mozaff. & Mehrabian	Boraginaceae	CR	1	0	4
Onosma zagrica Dehshiri	Boraginaceae	CR	1	0	4
Ophrys kurdistanica Renz	Orchidaceae	EN	2	0	8
Ophrys turcomanica Renz	Orchidaceae	CR	1	0	4
Opoidia galbanifera Lindl.	Apiaceae	CR	1	0	4
Opsicarpium insignis Mozaff.	Apiaceae	VU	8	21160	32
Oreophysa microphylla (Jaub. & Spach) Browicz	Fabaceae	VU	14	1956	56
Ornithogalum boissieri Bidarlord & F.Ghahrem.	Asparagaceae	CR	1	0	4
Ornithogalum khuzestanicum Heidaryan	Asparagaceae	EN	2	0	8
Ornithogalum marivanense Maroofi & Ghaderi	Asparagaceae	CR	1	0	4
Ornithogalum pycnanthum Wendelbo	Asparagaceae	EN	5	6516	20
Ornithogalum sanandajense Maroofi	Asparagaceae	CR	1	0	4

Orobanche eriophora Bornm & Gauba	Orobanchaceae	CR	2	0	4
Orobanche longibracteata Schiman-Czeika	Orobanchaceae	EN	4	165312	16
Orobanche pulchra Gilli	Orobanchaceae	EN	4	93917	16
Orobanche schwingenschussii Gilli	Orobanchaceae	CR	1	0	4
Otostegia michauxii Briq.	Lamiaceae	EN	2	0	8
Oxytropis aellenii Vassilcz.	Fabaceae	CR	1	0	4
Oxytropis azarbaijanica Podlech	Fabaceae	CR	1	0	4
Oxytropis bakhtiarica Maassoumi	Fabaceae	EN	2	0	8
Oxytropis bicornis Vassilcz	Fabaceae	VU	10	36133	40
Oxytropis binaludensis Vassilcz.	Fabaceae	VU	11	47339	44
Oxytropis chrysocarpa Boiss.	Fabaceae	VU	7	391011	28
Oxytropis cinerea Vassilcz.	Fabaceae	EN	3	63	12
Oxytropis compacta Maassoumi & Joharchi	Fabaceae	CR	2	0	4
Oxytropis gracillima Vassilcz.	Fabaceae	CR	1	0	4
Oxytropis guilanica Maassoumi & Moradi	Fabaceae	CR	1	0	4
Oxytropis hypsophila Bunge & Boiss.	Fabaceae	VU	11	47250	44
Oxytropis indurata Maassoumi	Fabaceae	CR	1	0	4
Oxytropis Iranica Vassilcz.	Fabaceae	EN	4	2166	16
Oxytropis javaherdehi Maassoumi	Fabaceae	EN	3	111	12
Oxytropis kermanica Freyn & Bornm	Fabaceae	EN	5	182674	20
Oxytropis kordkoyensis Maassoumi	Fabaceae	EN	2	0	8
Oxytropis kuchanensis Vassilcz.	Fabaceae	LC or NT	13	100100	52
Oxytropis mahneshanensis Maassoumi	Fabaceae	CR	2	0	4
Oxytropis masanderanensis Vassilcz	Fabaceae	EN	3	30727	12
Oxytropis neorechingeriana Vassilcz.	Fabaceae	EN	5	309	16
Oxytropis persica Boiss.	Fabaceae	VU	6	50047	24
Oxytropis pseudosuavis Maassoumi	Fabaceae	CR	1	0	4
Oxytropis rechingeri Vassilcz.	Fabaceae	EN	2	0	8
Oxytropis rhodontha Vassilcz.	Fabaceae	CR	1	0	4
Oxytropis rudbarensis Vassilcz.	Fabaceae	CR	1	0	4

Oxytropis sabzevarensis Maassoumi	Fabaceae	CR	1	0	4
Oxytropis salukensis Maassoumi	Fabaceae	CR	2	0	4
Oxytropis shahvarica Maassoumi	Fabaceae	CR	1	0	4
Oxytropis shirkuhi Vassilcz.	Fabaceae	CR	1	0	4
Oxytropis sivehensis Maassoumi	Fabaceae	EN	3	9	8
Oxytropis surmandehi Vassilcz.	Fabaceae	CR	1	0	4
Oxytropis szovitsii Boiss. & Buhse	Fabaceae	VU	12	129115	48
Oxytropis yazdi Vassilcz.	Fabaceae	CR	2	0	4
Oxytropis zangolehensis Vassilcz.	Fabaceae	CR	1	0	4
Paeonia wendelboi Ruksans & Zetterlund	Paeoniaceae	EN	3	1149	12
Papaver gaubae Cullen & Rech.f.	Papaveraceae	EN	2	0	8
Papaver persicum Lindl.	Papaveraceae	EN	4	15559	16
Papaver tenuifolium Boiss. & Hohen. ex Boiss.	Papaveraceae	LC or NT	13	67213	52
Paracaryum cyclhymenium (Boiss.)H.Riedl	Boraginaceae	LC or NT	22	345563	88
Paracaryum glandulosum Khatamsaz	Boraginaceae	CR	1	0	4
Paracaryum gracile Czern.	Boraginaceae	CR	1	0	4
Paracaryum khorassanicum Khatamsaz	Boraginaceae	CR	1	0	4
Paracaryum lalezarense Doostmohammadi & Mirtadzadini	Boraginaceae	CR	2	0	4
Paracaryum luristanicum Nab.	Boraginaceae	VU	6	35262	24
Paracaryum modestum Boiss. & Hausskn. ex Boiss.	Boraginaceae	LC or NT	13	208341	52
Paracaryum persicum (Boiss.) Boiss.	Boraginaceae	LC or NT	41	766777	160
Paracaryum pygmaeum (Rech.f.) Heller in D. Heller & C.C. Heyn	Boraginaceae	EN	4	193	12
Paracaryum tenerum Bornm.	Boraginaceae	CR	1	0	4
Paraquilegia caespitosa	Ranunculaceae	EN	5	1632	20
Paronychia bungei Boiss.	Caryophyllaceae	LC or NT	39	364703	152
Paronychia caespitosa Stapf	Caryophyllaceae	LC or NT	26	119740	104
Paronychia lordeganica Dinarvand & Assadi	Caryophyllaceae	CR	1	0	4
Pedicularis rechingeri Wendelbo	Orobanchaceae	EN	3	2901	12
Pedicularis straussii Hausskn.	Orobanchaceae	EN	3	78	12
Pentanema kurdistanicum Maroofi & Ghaderi	Asteraceae	CR	1	0	4

Pentanema multicaule Boiss.	Asteraceae	VU	6	3980	24
Pentanema pulicariiforme (DC.) Rech.f.	Asteraceae	LC or NT	17	128913	68
Petrorhagia macra (Boiss. & Hausskn.) Ball & Heywood	Caryophyllaceae	CR	1	0	4
Peucedanum chenur Mozaff. Bot. Zhurn	Apiaceae	CR	1	0	4
Peucedanum glaucopruinosum Rech.f.	Apiaceae	EN	4	17855	16
Peucedanum hyrcanicum Gholizadeh, Naqinezhad & Mozaff.	Apiaceae	CR	1	0	4
Peucedanum knappii Bornm.	Apiaceae	CR	1	0	4
Peucedanum pimenovii Mozaff. Bot. Zhurn	Apiaceae	CR	1	0	4
Peucedanum translucens Rech.f.	Apiaceae	CR	1	0	4
Phagnalon persicum Boiss.	Asteraceae	LC or NT	14	245622	56
Phelipanche pouyanii Joharchi & Vaezi	Orobanchaceae	CR	1	0	4
Phleum iranicum Bornm. & Gauba	Poaceae	VU	11	3769	36
Phlomis anisodonta Boiss.	Lamiaceae	LC or NT	59	405553	220
Phlomis aucheri Boiss.	Lamiaceae	LC or NT	37	235976	148
Phlomis chorassanica Bunge	Lamiaceae	CR	1	0	4
Phlomis elliptica Benth.	Lamiaceae	LC or NT	35	65779	136
Phlomis ghilanensis C. Koch	Lamiaceae	EN	2	0	4
Phlomis iranica Joharchi & Vaezi	Lamiaceae	CR	1	0	4
Phlomis lurestanica Jamzad	Lamiaceae	EN	3	359	12
Phlomis mazandaranica Jamzad	Lamiaceae	CR	1	0	4
Phlomis pachyphylla Rech.f.	Lamiaceae	VU	7	18840	28
Phlomis persica Boiss.	Lamiaceae	LC or NT	110	443435	424
Phlomoides adenantha Jaub. & Spach	Lamiaceae	LC or NT	35	345748	140
Phlomoides azerbaijanica Rech.f.	Lamiaceae	LC or NT	18	23639	68
Phlomoides binaludensis Salmaki & Joharchi	Lamiaceae	EN	6	3854	24
Phlomoides codonocalyx Rech.f.	Lamiaceae	EN	2	0	8
Phlomoides hyoscyamoides Boiss. & Buhse	Lamiaceae	LC or NT	19	110144	76
Phlomoides kermanica Ranjbar & C.Mahmoudi	Lamiaceae	CR	1	0	4
Phlomoides lanata Jamzad	Lamiaceae	EN	4	8327	16
Phlomoides pulvinaris Jaub. & Spach	Lamiaceae	LC or NT	20	190408	76

Phuopsis stylosa Benth. & Hook.f	Rubiaceae	LC or NT	18	36547	72
Physogeton acanthophyllus Jaub. & Spach	Amaranthaceae	LC or NT	19	764802	76
Physogeton occultus (Bge.) Assadi	Amaranthaceae	LC or NT	14	139627	56
Physogeton pedunculatus (Assadi) Assadi	Amaranthaceae	EN	3	17022	12
Physoptychis gnaphalodes Boiss.	Brassicaceae	LC or NT	49	407236	188
Pimpinella anisactis Rech.f.	Apiaceae	EN	4	151986	16
Pimpinella deverroides (Boiss.) Boiss.	Apiaceae	LC or NT	19	96845	72
Pimpinella dichotoma (Boiss. et Hausskn.) Wolff in Engler	Apiaceae	EN	7	102580	24
Pimpinella gedrosiaca Bornm.	Apiaceae	EN	5	24	20
Pimpinella gilanica Mozaff.	Apiaceae	CR	1	0	4
Pimpinella khayyamii Mozaff.	Apiaceae	EN	4	135	16
Pimpinella khorasanica Engstrand.	Apiaceae	EN	5	5082	20
Pimpinella tragioides (Boiss.) Benth. & Hook.f. ex Drude	Apiaceae	LC or NT	16	115221	64
Piptatherum denaense Hamzehee & Assadi	Poaceae	CR	1	0	4
Piptatherum molinioides Boiss.	Poaceae	VU	6	200204	24
Plagioloba derakii Khosravi & Eslami-Farouji	Brassicaceae	EN	5	44514	20
Plantago podlechii Akhani.	Plantaginaceae	CR	1	0	4
Platychaete aucheri (Boiss.) Boiss.	Asteraceae	LC or NT	25	184977	100
Platychaete mucronifolia (Boiss.) Boiss.	Asteraceae	VU	10	37187	40
Platychaete velutina Boiss. & Hausskn.	Asteraceae	EN	3	1250	12
Plocama ehrendorferi Mirtadz. & Bordbar	Rubiaceae	CR	1	0	4
Poa demavandica Assadi & Kavousi	Poaceae	CR	1	0	4
Poa golestanensis H. Scholz & Akhani.	Poaceae	CR	1	0	4
Polygala guilanica Sarvi & Faghir	Polygalaceae	CR	1	0	4
Polygala mazandaranica Sarvi & Faghir	Polygalaceae	CR	1	0	4
Polygala platyptera Bornm & Gauba	Polygalaceae	LC or NT	29	26801	108
Polygonum aridum Boiss. & Hausskn. in Boiss.	Polygonaceae	LC or NT	12	18409	48
Polygonum botuliforme Mozaffarian	Polygonaceae	CR	2	0	4
Polygonum dumosum Boiss.	Polygonaceae	VU	11	74702	44
Polygonum hyrcanicum Rech.f.	Polygonaceae	LC or NT	27	112164	108

Polygonum iranicum Mozaff.	Polygonaceae	EN	2	0	8
Polygonum salicornioides Jaub. & Spach	Polygonaceae	LC or NT	19	242769	72
Polygonum spinosum H. Gross	Polygonaceae	VU	6	13910	24
Polylophium involucratum (Pall.) Boiss.	Apiaceae	EN	5	3021	20
Polypodium iranicum Mazzoji	Polygonaceae	CR	1	0	4
Postia bombycina Boiss. & Hausskn.	Asteraceae	EN	4	6591	16
Postia puberula Boiss. & Hausskn.	Asteraceae	LC or NT	12	29236	48
Potentilla alborzensis Faghir & Attar	Rosaceae	CR	1	0	4
Potentilla aucheriana Th. Wolf	Rosaceae	LC or NT	19	28818	72
Potentilla diversidentata Faghir & Naqinezhad	Rosaceae	CR	1	0	4
Potentilla elvendensis Boiss. et Hohen.	Rosaceae	LC or NT	19	40722	72
Potentilla farsistanica Browicz	Rosaceae	EN	2	0	8
Potentilla flaccida Th. Wolf	Rosaceae	VU	7	22178	28
Potentilla gaubaeana Bornm.	Rosaceae	CR	1	0	4
Potentilla gilanica (Th. Wolf.)Th. Wolf	Rosaceae	CR	1	0	4
Potentilla humillis Mozaff.	Rosaceae	CR	1	0	4
Potentilla iranica (Rech.f.) Schiman-Czeika	Rosaceae	EN	4	87274	16
Potentilla kandavanensis Born. & Gauba	Rosaceae	CR	1	0	4
Potentilla mallota Boiss.	Rosaceae	EN	4	16361	16
Potentilla nuda Boiss.	Rosaceae	LC or NT	19	766080	76
Potentilla nurensis Boiss. & Hausskn.	Rosaceae	VU	11	89300	40
Potentilla petraea Willd. ex Schlecht.	Rosaceae	CR	1	0	4
Potentilla poteriifolia Boiss.	Rosaceae	VU	9	183864	36
Potentilla radiata Lehm.	Rosaceae	CR	1	0	4
Potentilla sangedehensis Faghir & Naqinezhad	Rosaceae	CR	1	0	4
Potentilla schiraziana Khatamsaz	Rosaceae	CR	1	0	4
Prangos calligonoides Rech.f.	Apiaceae	EN	3	20	8
Prangos cheilanthifolia Boiss.	Apiaceae	LC or NT	22	232582	88
Prangos crossoptera Herrnst. & Heyn	Apiaceae	EN	4	842	16
Prangos gauba (Bornm.) Herrnstadt & Heyn	Apiaceae	VU	10	139950	36

Prangos tuberculata Boiss. & Hausskn.	Apiaceae	LC or NT	12	148322	48
Primula gaubaeana Bornm.	Primulaceae	LC or NT	13	355814	52
Prunus ghahremanii (Maroofi, Attar & Vafadar) Falatoury	Rosaceae	DD	NA	NA	NA
Prunus mazandaranica Habibi, H.Maleki & Attar	Rosaceae	DD	NA	NA	NA
Prunus paradoxa (Dehshiri & Mozaff.) Falatoury	Rosaceae	DD	NA	NA	NA
Prunus yazdiana (Mozaff.) Falatoury	Rosaceae	DD	NA	NA	NA
Psephellus congestus (Wagenitz) Wagenitz	Asteraceae	EN	3	349	12
Psephellus khalkhalensis Ranjbar & Negaresh	Asteraceae	EN	3	12	12
Psephellus sennikovianus Negaresh	Asteraceae	DD	NA	NA	NA
Pseudocamelina aphragmodes (Boiss.) N. Busch	Brassicaceae	EN	5	16485	20
Pseudocamelina bakhtiarica Esmailbegi, Mirtadzadini & Al-Shehbaz	Brassicaceae	CR	1	0	4
Pseudocamelina camelinae (Boiss.) N.Busch	Brassicaceae	LC or NT	36	441547	144
Pseudocamelina campylocarpa (Boiss.) N. Busch	Brassicaceae	LC or NT	12	130069	48
Pseudocamelina glaucophylla (DC.) N. Busch	Brassicaceae	LC or NT	22	178469	84
Pseudocamelina kermanica Esmailbegi, Mirtadzadini & Al-Shehbaz	Brassicaceae	EN	5	3880	20
Pseudocamelina szowitsii (Boiss.) N. Busch	Brassicaceae	CR	2	0	4
Pseudocamelina violacea (Boiss.) N.Busch	Brassicaceae	EN	4	60594	16
Pseudofortuynia esfandiarii Hedge	Brassicaceae	EN	4	324	16
Pseudofortuynia leucoclada (Boiss.) Khosravi	Brassicaceae	LC or NT	20	166514	80
Pseudotrachydium kotschyi (Boiss.) Pimenov & Kljuykov	Apiaceae	LC or NT	21	122325	84
Pseudotrachydium pauciradiatum (Boiss. & Hohen.) Pimenov & Kljuykov	Apiaceae	VU	10	23080	40
Psychrogeton aellenii (Rech.f.) Grierson	Asteraceae	EN	2	0	8
Psychrogeton chionophilus (Boiss.) Krasch.	Asteraceae	CR	1	0	4
Pterocephalus ghahremanii Jamzad	Caprifoliaceae	CR	1	0	4
Pterocephalus lignosus Freyn & Bornm.	Caprifoliaceae	LC or NT	13	4647	52
Pterocephalus melanobasis Pau.	Caprifoliaceae	CR	1	0	4
Pterocephalus persicus Boiss.	Caprifoliaceae	LC or NT	22	230230	84
Pterocephalus ramianensis Ranjbar & Z.Ranjbar	Caprifoliaceae	CR	1	0	4
Pterocephalus wendelboi Rech.f.	Caprifoliaceae	CR	1	0	4
Pteropyrum gypsaceum Akhani & Doostmohammadi	Polygonaceae	LC or NT	14	14058	56

Pteropyrum jakdanense Doostmohammadi	Polygonaceae	EN	2	0	8
Pteropyrum macrocarpum Doostmohammadi & Akhani	Polygonaceae	LC or NT	11	42321	48
Pteropyrum zagricum Doostmohammadi & Akhani	Polygonaceae	VU	7	9486	28
Puschkinia advayana Rukšāns & Zubov	Asparagaceae	CR	1	0	4
Puschkinia avromanica Rukšāns & Zubov	Asparagaceae	EN	3	202	12
Puschkinia latifolia Rukšāns & Zubov	Asparagaceae	CR	1	0	4
Puschkinia parvula Rukšāns & Zubov	Asparagaceae	EN	2	0	8
Pycnocycla acanthorhipsis Rech.f.	Apiaceae	EN	4	5131	12
Pycnocycla bashagardiana Mozaffarian	Apiaceae	VU	9	10872	36
Pycnocycla musiformis Hedge & Lamond	Apiaceae	EN	4	68	16
Pyrus cordifolia Zamani & Attar	Rosaceae	EN	2	0	8
Pyrus ghahremanii Attar & Zamani	Rosaceae	CR	1	0	4
Pyrus giffanica Zamani & Attar	Rosaceae	CR	1	0	4
Pyrus glabra Boiss.	Rosaceae	LC or NT	16	38102	64
Pyrus kandevanica Ghahreman	Rosaceae	EN	5	3912	16
Pyrus longipedicellata Zamani & Attar	Rosaceae	EN	2	0	4
Pyrus mazanderanica Schonbeck-Temesy	Rosaceae	EN	8	3607	24
Ranunculus amblyolobus Boiss. & Hohen. in Boiss.	Ranunculaceae	LC or NT	24	60771	92
Ranunculus dalechanensis Iranshahr & Rech.f.	Ranunculaceae	EN	2	0	8
Ranunculus elbursensis Boiss.	Ranunculaceae	VU	11	25588	44
Ranunculus elymaiticus Boiss. & Hausskn.	Ranunculaceae	LC or NT	13	26612	52
Ranunculus eriorrhizus Boiss. & Buhse	Ranunculaceae	EN	7	22804	24
Ranunculus farsicus Rech.f.	Ranunculaceae	CR	1	0	4
Ranunculus koeiei Rech.f.	Ranunculaceae	CR	1	0	4
Ranunculus microflorus Pakravan	Ranunculaceae	CR	1	0	4
Ranunculus papyrocarpus Rech.f.	Ranunculaceae	VU	10	15158	40
Ranunculus pichleri Freyn	Ranunculaceae	VU	7	42055	28
Ranunculus renzii Iranshahr & Rech.f.	Ranunculaceae	CR	1	0	4
Ranunculus sahendicus Boiss. & Buhse	Ranunculaceae	EN	2	0	8
Ranunculus sojakii Iranshahr & Rech.f.	Ranunculaceae	EN	3	96	8

Ranunculus straussii Bornm.	Ranunculaceae	EN	7	555	24
Ranunculus termei Iranshahr & Rech.f.	Ranunculaceae	EN	3	1036	12
Ranunculus zenjanensis Iranshahr & Rech.f.	Ranunculaceae	EN	4	6959	16
Reseda buhseana Mull.-Arg.	Resedaceae	LC or NT	27	944702	108
Reseda bungei Boiss.	Resedaceae	LC or NT	39	181272	144
Reseda macrobotrys Boiss.	Resedaceae	VU	9	36092	36
Rhabdosciadium petiolare Boiss. & Hausskn.	Apiaceae	EN	3	5560	12
Rhabdosciadium straussii Hausskn. ex Beih.	Apiaceae	EN	4	5358	16
Rhamnus × mehreganii Alijanpoor & Khodayari	Ranunculaceae	EN	4	9417	16
Rhamnus cornifolia Boiss. & Hohen.	Rhamnaceae	LC or NT	41	184477	160
Rhamnus rahiminejadii Alijanpoor & Assadi	Rhamnaceae	CR	1	0	4
Rhaponticoides bachtiarica (Boiss. & Hausskn.) L. Martins	Asteraceae	CR	1	0	4
Rheum austro-iranicum Taheri & Assadi	Polygonaceae	CR	1	0	4
Rheum iranshahrii Taheri & Assadi	Polygonaceae	CR	1	0	4
Rheum kordestanicum Taheri & Assadi	Polygonaceae	VU	6	63307	24
Rheum neyshabourense B. Baradaran & A. Jafari	Polygonaceae	CR	1	0	4
Rheum persicum Los.	Polygonaceae	EN	4	16429	16
Rhopalosciadium stereocalyx Rech.f.	Apiaceae	CR	1	0	4
Ribes khorasanicum Saghafi & Assadi	Grossulariaceae	EN	3	166	12
Rindera bungei (Boiss.) Gurke in Engler & Prantl	Boraginaceae	EN	4	15243	16
Rindera media (Turrill) H.Riedl	Boraginaceae	CR	1	0	4
Rindera regia (Gmelin) Kusn.	Boraginaceae	CR	2	0	4
Rochelia mirheydari Reidl & Esfandiari	Boraginaceae	VU	9	87708	36
Rochelia retrosepala Khatamsaz	Boraginaceae	CR	1	0	4
Rosa arabica Khatamsaz & Koobaz	Rosaceae	CR	1	0	4
Rubia albicaulis Boiss.	Rubiaceae	LC or NT	20	170037	80
Rubia caramanica Bornm.	Rubiaceae	EN	8	1970	32
Rubia pauciflora Boiss.	Rubiaceae	LC or NT	21	54699	80
Rumex elbursensis Boiss.	Polygonaceae	VU	11	126014	44
Rumex ephedroides Bornm.	Polygonaceae	VU	8	34882	32

Rumex kandavanicus (Rech.f.) Rech.f.	Polygonaceae	CR	1	0	4
Salicornia iranica Akhani	Amaranthaceae	EN	4	8762	12
Salicornia persica Akhani	Amaranthaceae	VU	6	42081	24
Salicornia perspolitana Akhani	Amaranthaceae	EN	2	0	8
Salicornia sinus-persica Akhani	Amaranthaceae	VU	12	73671	44
Salicornia turanica Chatren. & Akhani	Amaranthaceae	EN	2	0	8
Salix baladehensis Maassoumi	Salicaceae	EN	3	62	8
Salix elymaitica Maassoumi	Salicaceae	CR	1	0	4
Salix firouzkuhensis Maassoumi	Salicaceae	EN	5	422	20
Salix issatissensis Maassoumi, Moeeni & Rahiminejad	Salicaceae	VU	8	46275	32
Salix lacus-tari Maassoumi & Kazempour	Salicaceae	EN	2	0	4
Salix viridiformis Maassoumi	Salicaceae	CR	1	0	4
Salsola abarghuensis Assadi	Amaranthaceae	EN	4	955	12
Salsola austro-iranica Akhani	Amaranthaceae	CR	1	0	4
Salsola persica Bunge ex Boiss.	Amaranthaceae	EN	9	2058	28
Salsola yazdiana Assadi	Amaranthaceae	LC or NT	22	190530	84
Salvia aristata Aucher ex Benth. in DC.	Lamiaceae	LC or NT	59	295347	236
Salvia bazmanica Rech.f. & Esfand.	Lamiaceae	VU	6	13775	24
Salvia chorassanica Bunge	Lamiaceae	VU	8	6258	28
Salvia eremophila Boiss.	Lamiaceae	LC or NT	58	426116	228
Salvia hypochionaea Boiss.	Lamiaceae	EN	3	191	12
Salvia hypoleuca Benth. in DC.	Lamiaceae	LC or NT	84	26589	296
Salvia jamzadii Mozaffarian	Lamiaceae	EN	3	1273	12
Salvia kermanshahensis Rech.f.	Lamiaceae	VU	6	4193	24
Salvia lachnocalyx Hedge	Lamiaceae	EN	6	59	16
Salvia oligophylla Auch. ex Benth. in DC.	Lamiaceae	VU	7	5248	28
Salvia persepolitana Boiss.	Lamiaceae	LC or NT	16	87419	64
Salvia sahendica Boiss. & Buhse	Lamiaceae	LC or NT	29	25708	112
Salvia sclareopsis Bornm. ex Hedge	Lamiaceae	LC or NT	13	91832	52
Salvia shahkuhmahalei Akhani	Lamiaceae	CR	1	0	4

Salvia sharifii Rech.f. & Esfand.	Lamiaceae	LC or NT	31	580966	120
Salvia urmiensis Bunge	Lamiaceae	LC or NT	13	109728	52
Salvia wendelboi Hedge	Lamiaceae	EN	2	0	8
Saponaria bodeana Boiss.	Caryophyllaceae	VU	8	3551	32
Saponaria esfandiarii Assadi	Caryophyllaceae	CR	1	0	4
Saponaria iranica Dashti, Assadi & Sharifnia	Caryophyllaceae	CR	1	0	4
Satureja atropatana Bunge	Lamiaceae	EN	5	140	16
Satureja avromanica Maroofi	Lamiaceae	CR	1	0	4
Satureja bachtiarica Bunge	Lamiaceae	LC or NT	43	346250	168
Satureja edmondi Briquet	Lamiaceae	VU	9	8276	36
Satureja isophylla Rech.f.	Lamiaceae	VU	15	8494	56
Satureja kallarica Jamzad	Lamiaceae	EN	3	48	8
Satureja kermanica Payandeh, Bordbar & Mirtadz.	Lamiaceae	EN	4	107	16
Satureja kermanshahensis Jamzad	Lamiaceae	CR	1	0	4
Satureja khuzistanica Jamzad	Lamiaceae	EN	3	84	12
Satureja rechingeri Jamzad	Lamiaceae	EN	2	0	4
Satureja sahendica Bornm.	Lamiaceae	LC or NT	20	146085	80
Saxifraga iranica Bornm.	Saxifragaceae	VU	10	620	36
Saxifraga koelzii Schönb.-Tem.	Saxifragaceae	CR	2	0	4
Saxifraga mazanderanica Rech.f.	Saxifragaceae	EN	2	0	8
Saxifraga ramsarica Jamzad	Saxifragaceae	EN	2	0	4
Saxifraga wendelboi Schönb.-Tem.	Saxifragaceae	EN	7	268	24
Scabiosa esfandiarii Jamzad	Caprifoliaceae	CR	1	0	4
Scabiosa schimperiana Boiss. & Buhse	Caprifoliaceae	EN	5	1145	20
Scilla bisotunensis Speta	Asparagaceae	EN	4	2459	16
Scilla gorganica Speta	Asparagaceae	VU	7	3558	28
Scilla greilhuberi Speta	Asparagaceae	VU	9	94356	36
Scilla khorassanica Meikle	Asparagaceae	VU	7	23119	24
Sclerochorton haussknechtii Boiss.	Apiaceae	CR	2	0	4
Sclerorhachis binaludensis Sonboli	Asteraceae	EN	10	200	20

Sclerorhachis ferdowsii Hassanpour, Moazzeni & Sonboli	Asteraceae	EN	9	2786	24
Sclerorhachis leptoclada Rech.f.	Asteraceae	EN	3	4284	12
Scorzonera alborzensis S.R.Safavi & Amini Rad	Asteraceae	CR	1	0	4
Scorzonera flaccida Rech.f.	Asteraceae	EN	3	8	8
Scorzonera grossheimii Lipsch. & Vassilcz	Asteraceae	VU	8	185952	32
Scorzonera helodes Rech.f.	Asteraceae	EN	2	0	8
Scorzonera intricata Boiss.	Asteraceae	LC or NT	22	105290	84
Scorzonera ispahanica Boiss.	Asteraceae	VU	7	28945	28
Scorzonera joharchii S.R.Safavi	Asteraceae	EN	2	0	8
Scorzonera kandavanica Rech.f.	Asteraceae	EN	7	239	24
Scorzonera karkasensis Safavi	Asteraceae	CR	2	0	4
Scorzonera luristanica Rech.f.	Asteraceae	LC or NT	34	234414	132
Scorzonera microcalathia (Rech.f.) Rech.f.	Asteraceae	VU	11	229269	44
Scorzonera mucida Rech.f.	Asteraceae	LC or NT	65	935462	248
Scorzonera nivalis Boiss. & Hausskn.	Asteraceae	CR	1	0	4
Scorzonera persica Boiss. & Buhse	Asteraceae	EN	2	0	8
Scorzonera perspolitana Boiss.	Asteraceae	LC or NT	16	162619	64
Scorzonera psychrophila Boiss. & Hausskn.	Asteraceae	EN	3	871	12
Scorzonera renzii Rech.f.	Asteraceae	VU	6	22098	24
Scorzonera rupicola Hausskn.	Asteraceae	LC or NT	19	98989	76
Scorzonera stenocephala Boiss.	Asteraceae	LC or NT	14	162051	52
Scorzonera subaphylla Boiss.	Asteraceae	EN	6	3282	24
Scorzonera wendelboi Rech.f.	Asteraceae	EN	3	12	12
Scorzonera xylobasis Rech.f.	Asteraceae	CR	1	0	4
Scrophularia alhagioides Attar & Joharchi	Scrophulariaceae	CR	1	0	4
Scrophularia atroglandulosa Grau	Scrophulariaceae	EN	2	0	4
Scrophularia attariae Ranjbar & Rahchamani	Scrophulariaceae	EN	3	214	12
Scrophularia crassicaulis Boiss.	Scrophulariaceae	VU	11	4022	44
Scrophularia crassipedunculata Attar & Joharchi	Scrophulariaceae	EN	5	253	20
Scrophularia crassiuscula Grau	Scrophulariaceae	VU	11	25172	44

Scrophularia denaensis Attar	Scrophulariaceae	CR	2	0	4
Scrophularia dianatnejadii Ranjbar & Rahch.	Scrophulariaceae	CR	1	0	4
Scrophularia elbursensis Bornm.	Scrophulariaceae	LC or NT	19	604872	72
Scrophularia elymaitica Mozaff.	Scrophulariaceae	CR	1	0	4
Scrophularia farinosa Boiss.	Scrophulariaceae	LC or NT	18	30607	68
Scrophularia flava Grau	Scrophulariaceae	CR	1	0	4
Scrophularia frigida Boiss.	Scrophulariaceae	LC or NT	68	1163706	264
Scrophularia gaubae Bornm.	Scrophulariaceae	LC or NT	13	49247	48
Scrophularia glauca Decne. ex Benth. in DC.	Scrophulariaceae	LC or NT	22	79917	88
Scrophularia gorganica Rech.f.	Scrophulariaceae	VU	7	42821	24
Scrophularia iranica Attar	Scrophulariaceae	CR	1	0	4
Scrophularia ispahanica Attar & Nowrouzi	Scrophulariaceae	CR	1	0	4
Scrophularia kermanica Ghahreman & Mirtadzadini	Scrophulariaceae	CR	1	0	4
Scrophularia khorassanica Attar & Joharchi	Scrophulariaceae	EN	3	32	12
Scrophularia maharluica Ranjbar & Rahchamani	Scrophulariaceae	EN	2	0	8
Scrophularia megalantha Rech.f.	Scrophulariaceae	LC or NT	17	13130	64
Scrophularia oxysepala Boiss.	Scrophulariaceae	VU	11	3779	44
Scrophularia papyracea Attar	Scrophulariaceae	CR	1	0	4
Scrophularia rechingeri Grau	Scrophulariaceae	EN	6	2474	20
Scrophularia rostrata Boiss. & Buhse	Scrophulariaceae	LC or NT	17	86776	60
Scrophularia sardashtensis Ranjbar & Rahch.	Scrophulariaceae	CR	1	0	4
Scrophularia schiraziana Attar & Hatami	Scrophulariaceae	EN	3	37534	12
Scrophularia shulabadensis Attar & Hamzeh'ee	Scrophulariaceae	CR	1	0	4
Scrophularia subaphylla Boiss.	Scrophulariaceae	LC or NT	27	526921	104
Scrophularia tortuosissima Attar & Joharchi	Scrophulariaceae	CR	1	0	4
Scrophularia valida Grau	Scrophulariaceae	EN	4	65	16
Scrophularia xylobasis Rech.f.	Scrophulariaceae	EN	2	0	8
Scutellaria arakensis Jamzad & Safikhani	Lamiaceae	EN	4	33131	16
Scutellaria bornmuelleri Hausskn. ex Bornm.	Lamiaceae	VU	9	9903	36
Scutellaria farsistanica Rech.f.	Lamiaceae	LC or NT	20	52312	76

Scutellaria fragillima Rech.f.	Lamiaceae	EN	2	0	8
Scutellaria glechomoides Boiss.	Lamiaceae	VU	15	50629	48
Scutellaria lurestanica Jamzad & Mehrnia	Lamiaceae	CR	1	0	4
Scutellaria multicaulis Boiss.	Lamiaceae	LC or NT	100	690724	372
Scutellaria nepetifolia Benth.	Lamiaceae	LC or NT	23	211152	88
Scutellaria patonii Jamzad & Safikhani	Lamiaceae	VU	12	434139	44
Scutellaria persica Bornm.	Lamiaceae	VU	10	52109	40
Scutellaria pinnatifida A. Ham.	Lamiaceae	LC or NT	108	500351	420
Scutellaria ramazanica Parsa	Lamiaceae	CR	1	0	4
Scutellaria szovitziana Bunge	Lamiaceae	CR	1	0	4
Scutellaria theobromina Rech.f.	Lamiaceae	LC or NT	19	9632	76
Scutellaria xylorrhiza Bornm.	Lamiaceae	EN	2	0	8
Secale ciliatiglume (Boiss.) Grossh.	Poaceae	CR	1	0	4
Sedum callichroum Boiss.	Crassulaceae	EN	3	8187	12
Sedum elburzense Akhiani & Assadi	Crassulaceae	EN	3	631	12
Sedum kotschyanum Boiss.	Crassulaceae	VU	8	83330	32
Semenovia dichotoma (Boiss.) Manden.	Apiaceae	VU	8	5918	32
Semenovia frigida (Boiss.) Hausskn.	Apiaceae	LC or NT	14	57550	56
Semenovia subscaposa (Rech.f.) Alava	Apiaceae	EN	5	37008	20
Semenovia suffruticosa (Freyn & Bornm.) Manden.	Apiaceae	EN	5	24221	20
Semenovia trajioides (Boiss.) Manden.	Apiaceae	LC or NT	23	612052	88
Sempervivum atropatanum Parnell	Crassulaceae	VU	11	7653	36
Sempervivum iranicum Bornm. & Gauba	Crassulaceae	LC or NT	26	108465	88
Senecio eligulatus B. Nord., Moussavi & Djavadi	Asteraceae	CR	1	0	4
Senecio iranicus B. Nord.	Asteraceae	CR	3	12	12
Senecio joharchii F.Ghahrem.	Asteraceae	CR	1	0	4
Senecio kotschyanus Boiss.	Asteraceae	EN	2	0	8
Senecio lipskyi Lomak.	Asteraceae	VU	7	7698	24
Senecio subnivalis Ajani	Asteraceae	EN	2	0	8
Senecio vulcanicus Boiss.	Asteraceae	EN	5	2829	20

Serratula bachtiarica Boiss. & Hausskn.	Asteraceae	CR	1	0	4
Serratula calcarea Mozaff.	Asteraceae	CR	1	0	4
Serratula gracillima Rech.f.	Asteraceae	CR	1	0	4
Serratula melanocheila Boiss. & Hausskn.	Asteraceae	CR	1	0	4
Serratula suffulta Rech.f.	Asteraceae	CR	1	0	4
Serratula viciifolia Boiss. & Hausskn.	Asteraceae	EN	3	46789	12
Sideritis khalkhalica Bidarlord & Jamzad	Lamiaceae	CR	1	0	4
Silene albescens Boiss.	Caryophyllaceae	LC or NT	19	178708	76
Silene aminii Assadi	Caryophyllaceae	CR	1	0	4
Silene aminiradii Gholipour	Caryophyllaceae	EN	4	13913	16
Silene avromana Boiss. & Hausskn.	Caryophyllaceae	LC or NT	15	289872	60
Silene bakhtiarica Assadi	Caryophyllaceae	CR	1	0	4
Silene caroli-henrici Melzh.	Caryophyllaceae	EN	2	0	8
Silene circumcarmanica F.Jafari, Gholipour, Mirtadz. & Pourmirzaei	Caryophyllaceae	EN	5	5865	16
Silene daenensis Melzh.	Caryophyllaceae	EN	4	14422	16
Silene demawendica Bornm.	Caryophyllaceae	CR	1	0	4
Silene dschuparensis Bornm.	Caryophyllaceae	CR	1	0	4
Silene elymaitica Bornm.	Caryophyllaceae	LC or NT	13	39667	52
Silene erysimifolia Stapf	Caryophyllaceae	VU	9	79623	36
Silene farsistanica Melzh.	Caryophyllaceae	VU	7	7053	24
Silene ferdowsii Joharchi	Caryophyllaceae	EN	3	1214	12
Silene gaubae Bornm. & Gauba	Caryophyllaceae	EN	3	63	12
Silene gertraudiae Melzh.	Caryophyllaceae	VU	7	213221	28
Silene ghahremaninejadii Hoseini & Assadi	Caryophyllaceae	CR	1	0	4
Silene goniocaula Boiss.	Caryophyllaceae	VU	10	281189	40
Silene gynodioica Ghaz.	Caryophyllaceae	LC or NT	39	696355	148
Silene hirticalyx Boiss. & Hausskn.	Caryophyllaceae	CR	1	0	4
Silene karajensis Assadi	Caryophyllaceae	EN	2	0	8
Silene kurdistanica Maroofi & Attar	Caryophyllaceae	CR	1	0	4
Silene lineata Boiss. & Buhse	Caryophyllaceae	CR	1	0	4

Silene lulakabadensis Heidari, F.Ghahrem. & Assadi	Caryophyllaceae	CR	1	0	4
Silene meyeri Fenzl ex Boiss. & Buhse	Caryophyllaceae	LC or NT	18	171245	68
Silene mishudaghensis Gholipour & Parsa Khanghah	Caryophyllaceae	CR	1	0	4
Silene nizvana Melzeh.	Caryophyllaceae	EN	3	19	8
Silene nurensis Boiss. & Hausskn.	Caryophyllaceae	VU	7	100692	28
Silene oligophylla Melzh.	Caryophyllaceae	CR	1	0	4
Silene orientoalborzensis F.Jafari & Mirtadz.	Caryophyllaceae	CR	1	0	4
Silene oxelmanii A. Gholipour	Caryophyllaceae	EN	4	16913	16
Silene palinotricha Fenzl ex Boiss.	Caryophyllaceae	EN	5	3666	12
Silene parrowiana Boiss. & Hausskn. in Boiss.	Caryophyllaceae	CR	2	0	4
Silene penduliflora F.Jafari, Keshavarzi & Doostm.	Caryophyllaceae	EN	2	0	8
Silene persepolitana Melzh.	Caryophyllaceae	EN	3	1252	12
Silene persica Boiss.	Caryophyllaceae	EN	6	7946	24
Silene pravitziana Rech.f.	Caryophyllaceae	CR	1	0	4
Silene pseudaucheriana Melzh.	Caryophyllaceae	EN	3	24373	12
Silene pseudonurensis Melzh.	Caryophyllaceae	EN	2	0	8
Silene rasvandica Melzh.	Caryophyllaceae	EN	2	0	8
Silene renzii Melzh.	Caryophyllaceae	CR	1	0	4
Silene sciaphila Melzh. & Rech.f.	Caryophyllaceae	CR	1	0	4
Silene shahrudensis Rech.f.	Caryophyllaceae	EN	2	0	8
Silene sojakii Melzh.	Caryophyllaceae	EN	3	2880	12
Silene stapfii Melzh.	Caryophyllaceae	EN	3	1390	12
Silene thyrsiantha F.Jafari, Mirtadz. & Keshavarzi	Caryophyllaceae	CR	1	0	4
Silene tragacantha Fenzl ex Boiss.	Caryophyllaceae	EN	2	0	8
Sisymbrium gaubae Rech.f. & Bornm.	Brassicaceae	VU	8	68638	28
Sisymbrium kermanicum Khodashenas & Mirtadzadini	Brassicaceae	CR	1	0	4
Solenanthus bakhtiaricus Khatamsaz	Boraginaceae	CR	2	0	4
Sorbus luristanica (Bornm.) Schonbeck-Temesy	Rosaceae	EN	4	8084	16
Sorbus tiliifolia H.Zare, Amini & Assadi	Rosaceae	CR	3	8	8
Spiraea sheikhii Zare	Rosaceae	CR	1	0	4

Stachys acerosa Boiss.	Lamiaceae	LC or NT	90	308195	352
Stachys asterocalyx Rech.f.	Lamiaceae	EN	5	20148	20
Stachys aucheri Benth.	Lamiaceae	VU	10	26340	40
Stachys ixodes Boiss. & Hausskn. ex Boiss.	Lamiaceae	LC or NT	20	43771	80
Stachys kermanshahensis Rech.f.	Lamiaceae	VU	8	8079	32
Stachys koelzii Rech.f.	Lamiaceae	EN	3	44	12
Stachys laxa Boiss. & Buhse	Lamiaceae	LC or NT	38	39094	148
Stachys lurestanica Jamzad	Lamiaceae	CR	1	0	4
Stachys obtusicrena Boiss.	Lamiaceae	LC or NT	41	54434	144
Stachys persepolitana Boiss.	Lamiaceae	LC or NT	17	202548	68
Stachys pilifera Benth.	Lamiaceae	LC or NT	88	118917	332
Stachys subaphylla Rech.f.	Lamiaceae	VU	7	1750	28
Stachys veroniciformis Rech.f.	Lamiaceae	VU	6	30606	24
Stellaria assadii Mahdavi	Caryophyllaceae	VU	9	40698	24
Stellaria scaturiginella Rech.f.	Caryophyllaceae	CR	1	0	4
Stelleropsis iranica Pobedimova	Thymelaeaceae	EN	3	26248	12
Stenotaenia elbursensis Bornm.	Apiaceae	CR	1	0	4
Stenotaenia haussknechtii Boiss.	Apiaceae	CR	1	0	4
Stenotaenia nudicaulis Boiss.	Apiaceae	VU	9	29031	36
Sterigmostemum longistylum (Boiss.) Bornm.	Brassicaceae	LC or NT	13	209563	52
Stipa atriseta Stapf ex Bor	Poaceae	EN	5	3032	20
Stipa gaubae Bor	Poaceae	EN	3	7442	12
Stipa haussknechtii Boiss.	Poaceae	VU	8	161875	32
Straussiella purpurea (Bge.) Hausskn.	Brassicaceae	VU	10	31169	40
Stroganowia persica Busch	Brassicaceae	VU	10	27856	40
Suaeda iranshahrii Akhani & Freitag	Amaranthaceae	CR	1	0	4
Suaeda khalijefarsica Akhani	Amaranthaceae	CR	1	0	4
Tamarix humboldtiana Akhani, Borsch & N. Samadi	Tamaricaceae	CR	1	0	4
Tamarix serotina Bunge ex Boiss.	Tamaricaceae	EN	4	203796	16
Tanacetum archibaldii Podl.	Asteraceae	CR	1	0	4

Tanacetum bachtiaricum Mozaff.	Asteraceae	EN	3	319	12
Tanacetum budjnurdense (Rech.f.) Tzvel.	Asteraceae	EN	2	0	8
Tanacetum dumosum Boiss.	Asteraceae	LC or NT	14	19140	56
Tanacetum elbursense Mozaffarian	Asteraceae	CR	1	0	4
Tanacetum hololeucum (Bornm.) Podl.	Asteraceae	LC or NT	22	3046	84
Tanacetum isfahanicum Sonboli & Naderifar	Asteraceae	EN	2	0	8
Tanacetum joharchii Sonboli & Kaz.Osaloo	Asteraceae	CR	1	0	4
Tanacetum khorassanicum (Krasch.) Parsa	Asteraceae	VU	7	25873	28
Tanacetum kurdistanicum Maroofi & Rastegar	Asteraceae	CR	2	0	4
Tanacetum lingulatum (Boiss.) Bornm.	Asteraceae	LC or NT	16	171474	64
Tanacetum paradoxum Bornm.	Asteraceae	EN	3	36	12
Tanacetum salsugineum Podl.	Asteraceae	CR	1	0	4
Tanacetum sonbolii Mozaffarian	Asteraceae	CR	1	0	4
Tanacetum stapfianum (Rech.f.) Podl.	Asteraceae	EN	3	279	12
Tanacetum tarighii Sonboli	Asteraceae	CR	1	0	4
Tanacetum tenuisectum (Boiss.) Podl.	Asteraceae	LC or NT	23	122272	92
Tanacetum trifoliolatum Podl.	Asteraceae	CR	1	0	4
Tanacetum urmiense Tabad, Maroofi & Rastegar	Asteraceae	EN	2	0	8
Taraxacum azerbaijanicum Soest	Asteraceae	VU	8	110364	32
Taraxacum baluchistanicum Soest	Asteraceae	CR	1	0	4
Taraxacum darbandense Soest	Asteraceae	EN	3	367	12
Taraxacum hepaticolor Soest	Asteraceae	CR	1	0	4
Taraxacum hydrophilum Soest	Asteraceae	EN	3	695	12
Taraxacum kalchanicum Soest	Asteraceae	EN	2	0	8
Taraxacum leonardii Soest	Asteraceae	DD	NA	NA	NA
Taraxacum plicatulum Soest	Asteraceae	EN	2	0	8
Taraxacum ruberuliforme Soest	Asteraceae	EN	3	31936	12
Taraxacum vagum Soest	Asteraceae	CR	1	0	4
Taverniera echinata Mozaffarian	Fabaceae	EN	2	0	8
Telephium eriglaucum Williama	Caryophyllaceae	VU	9	50949	36

Tetrataenium nephrophyllum (Leute) Manden.	Apiaceae	EN	2	0	8
Teucrium abolhayatensis Ranjbar & Mahmoudi	Lamiaceae	CR	1	0	4
Teucrium macrum Boiss. & Hausskn. ex Boiss.	Lamiaceae	CR	1	0	4
Teucrium persicum Boiss.	Lamiaceae	LC or NT	14	57167	56
Thalictrum mazandaranicum Pakravan & Assadi	Ranunculaceae	CR	1	0	4
Thalictrum tacabicum Pakravan & Assadi	Ranunculaceae	CR	1	0	4
Thecocarpus meifolius Boiss.	Apiaceae	LC or NT	26	90874	104
Thlaspi inhumile Ponert	Brassicaceae	CR	1	0	4
Thlaspi kurdicum Hedge	Brassicaceae	EN	2	0	8
Thlaspi maassoumii Mozaffarian	Brassicaceae	CR	1	0	4
Thlaspi pulvinata Mozaffarian	Brassicaceae	EN	3	28095	12
Thlaspi stenocarpum (Boiss.) Hedge	Brassicaceae	VU	7	15466	28
Thlaspi tenue (Boiss. & Buhse) Hedge.	Brassicaceae	EN	4	327	16
Thymus carmanicus Jalas	Lamiaceae	VU	12	520081	44
Thymus daenensis Celak.	Lamiaceae	LC or NT	81	532719	320
Thymus marandensis Jamzad	Lamiaceae	CR	1	0	4
Thymus persicus (Ronniger ex Rech.f.) Jalas	Lamiaceae	EN	6	2280	24
Thymus pubescens Boiss. & Kotschy ex Celak.	Lamiaceae	LC or NT	82	476200	312
Tilia sabetii H.Zare	Malvaceae	EN	4	21	12
Tilia stellatopilosa H.Zare, Amini & Assadi	Malvaceae	CR	3	8	8
Trachydium depressum Boiss.	Apiaceae	LC or NT	27	489660	104
Trachydium eriocarpum Bornm. & Gauba	Apiaceae	EN	4	121	16
Trachydium kotschyi (Boiss.) Boiss.	Apiaceae	LC or NT	27	139506	104
Trachydium pauciradiatum (Boiss. & Hohen.) Rech.f.	Apiaceae	LC or NT	15	33091	56
Trachyspermum reginei Ajani & Mozzaff.	Apiaceae	CR	1	0	4
Tragopogon acanthocarpus Boiss.	Asteraceae	LC or NT	26	188617	104
Tragopogon caricifolius Boiss.	Asteraceae	LC or NT	74	830698	288
Tragopogon erostris Boiss. & Hausskn.	Asteraceae	EN	3	1237	12
Tragopogon gongylorrhizus Rech.f.	Asteraceae	VU	10	6171	40
Tragopogon jezdianus Boiss. & Buhse	Asteraceae	LC or NT	17	519126	68

Tragopogon kotschyi Boiss.	Asteraceae	LC or NT	29	18001	96
Tragopogon kurdicus Safavi & Maroofi	Asteraceae	CR	1	0	4
Tragopogon maturatus Boiss.	Asteraceae	EN	2	0	8
Tragopogon porphyrocephalus Rech.f.	Asteraceae	EN	5	250739	20
Tragopogon rezaiyensis Rech.f.	Asteraceae	VU	8	69896	32
Tragopogon stroterocarpus Rech.f.	Asteraceae	EN	2	0	8
Trichodesma aellenii H.Riedl	Boraginaceae	EN	3	7721	12
Trichodesma aucheri DC.	Boraginaceae	LC or NT	31	219658	124
Trichodesma elymaiticum Mozaffarian	Boraginaceae	CR	1	0	4
Trichodesma macrocarpum Rech.f. Aell. & Esfand.	Boraginaceae	CR	1	0	4
Tricholepis edmondsonii Rech.f.	Asteraceae	CR	1	0	4
Trifolium longidentatum Nabelek	Fabaceae	EN	2	0	8
Trifolium mazanderanicum Rech.f.	Fabaceae	CR	1	0	4
Trifolium radicosum Boiss. & Hohen.	Fabaceae	LC or NT	12	101885	48
Trigonella aphanoneura Rech.f.	Fabaceae	CR	1	0	4
Trigonella bakhtiarica Ranjbar & Z.Hajmoradi	Fabaceae	CR	1	0	4
Trigonella disperma Bornm. ex Vassilcz.	Fabaceae	LC or NT	24	126778	96
Trigonella elliptica Boiss.	Fabaceae	VU	7	454139	24
Trigonella khalkhalica Ranjbar & Z. Hajmoradi	Fabaceae	CR	1	0	4
Trigonella latialata (Bornm.) Vassilcz.	Fabaceae	EN	3	185	12
Trigonella persica Boiss.	Fabaceae	LC or NT	22	193913	88
Trigonella stenocarpa Rech.f.	Fabaceae	EN	3	3841	12
Trigonella subenervis Rech.f.	Fabaceae	CR	1	0	4
Trigonella teheranica Bornm.	Fabaceae	VU	13	1824	48
Trigonella yasujensis ranjbar	Fabaceae	CR	1	0	4
Trigonosciadium brachytaenium (Boiss.) Alava	Apiaceae	VU	11	73639	44
Trisetum bungei Boiss.	Poaceae	EN	4	6639	16
Triticum persicum Vavilov	Poaceae	DD	NA	NA	NA
Tulipa brinkii J.de Groot & Zonn.	Liliaceae	CR	1	0	4
Tulipa harazensis Rech.f.	Liliaceae	CR	1	0	4

Tulipa lorestanica Rukšāns & Zubov	Liliaceae	CR	1	0	4
Tulipa montana Lindl.	Liliaceae	LC or NT	29	402921	112
Tulipa sarvestanica Alipour & Majidi	Liliaceae	DD	NA	NA	NA
Tulipa ulophylla Wendelbo	Liliaceae	EN	4	6843	16
Tulipa urumiensis Stapf	Liliaceae	CR	1	0	4
Tulipa wendelboi Matin & Iranshahr	Liliaceae	CR	1	0	4
Typha azerbaijanensis Hamdi & Assadi	Typhaceae	CR	1	0	4
Typha kalatensis Assadi & Hamdi	Typhaceae	EN	4	215	12
Ulmus boissieri Graudz	Ulmaceae	VU	8	109794	32
Ungernia flava Boiss. & Hausskn. in Boiss.	Amaryllidaceae	LC or NT	12	85106	48
Verbascum albidiflorum Ranjbar & Nouri	Scrophulariaceae	CR	1	0	4
Verbascum aucheri (Boiss.) Hub.-Mor.	Scrophulariaceae	LC or NT	27	61249	100
Verbascum austroiranicum Hub.-Mor.	Scrophulariaceae	EN	4	487	16
Verbascum azerbaijanense Sharifnia & Assadi	Scrophulariaceae	CR	1	0	4
Verbascum birjandense Sotoodeh	Scrophulariaceae	DD	NA	NA	NA
Verbascum bornmuellerianum Hub.- Mor.	Scrophulariaceae	EN	2	0	8
Verbascum carmanicum (Bornm.) Hub.-Mor.	Scrophulariaceae	EN	4	3599	16
Verbascum disjectum (Murb.) Hub.-Mor.	Scrophulariaceae	VU	10	48560	40
Verbascum farsistanicum (Murb.) Hub.- Mor	Scrophulariaceae	LC or NT	54	325342	212
Verbascum gabrieliae (Bronm.) Hub.-Mor.	Scrophulariaceae	EN	3	696	12
Verbascum gilanicum Mozaff.	Scrophulariaceae	CR	1	0	4
Verbascum hasarense Freyn & Bornm. in Freyn	Scrophulariaceae	EN	4	14286	16
Verbascum haussknechtianum Hub.-Mor.	Scrophulariaceae	EN	5	11276	20
Verbascum intricatum (Benth.) O. Kuntze	Scrophulariaceae	LC or NT	14	34243	52
Verbascum kermanense Hub.-Mor.	Scrophulariaceae	EN	3	3704	12
Verbascum kochiiforme Boiss. & Hausskn.	Scrophulariaceae	LC or NT	15	33771	60
Verbascum lyprocarpum (Murb.) Hub.-Mor.	Scrophulariaceae	EN	3	37206	12
Verbascum parsana Sotoodeh, Attar & Civeyrel	Scrophulariaceae	CR	1	0	4
Verbascum scoparium Mozaffarian	Scrophulariaceae	EN	3	565	12
Verbascum shahsavarensis Sotoodeh, Attar & Civeyrel	Scrophulariaceae	CR	1	0	4

Verbascum straussii (Bornm.) Hub.- Mor	Scrophulariaceae	VU	9	25379	36
Verbascum sublobatum Murb.	Scrophulariaceae	VU	11	45119	44
Verbascum urumiense Sotoodeh	Scrophulariaceae	CR	1	0	4
Veronica acrotheca Bornm. & Gauba	Plantaginaceae	LC or NT	26	79453	100
Veronica aucheri Boiss.	Plantaginaceae	LC or NT	18	24924	68
Veronica bungei Boiss.	Plantaginaceae	CR	1	0	4
Veronica chionantha Bornm.	Plantaginaceae	EN	5	60	20
Veronica daranica Saeidi & Ghahr.	Plantaginaceae	EN	2	0	8
Veronica euphrasiifolia Link	Plantaginaceae	CR	1	0	4
Veronica farinosa Hausskn.	Plantaginaceae	LC or NT	18	71651	72
Veronica fragilis Boiss. & Hausskn.	Plantaginaceae	VU	12	21663	48
Veronica francispetae M. A. Fischer	Plantaginaceae	VU	6	11833	24
Veronica kopetdaghensis B. Fedtsch. ex Boriss.	Plantaginaceae	EN	3	306	12
Veronica kurdica Benth.	Plantaginaceae	LC or NT	35	355424	136
Veronica kurdistanica Advay	Plantaginaceae	EN	2	0	8
Veronica longipedicellata Saeidi	Plantaginaceae	CR	2	0	4
Veronica mazanderanae Wendelbo	Plantaginaceae	VU	10	54408	40
Veronica mirabilis Wendelbo	Plantaginaceae	EN	4	1043	12
Veronica paederotae Boiss.	Plantaginaceae	EN	7	1689	28
Veronica rechingeri M. A. Fischer	Plantaginaceae	LC or NT	16	13256	60
Veronica rubrifolia Boiss.	Plantaginaceae	VU	12	257287	40
Vicia aucheri Jaub. & Spach	Fabaceae	CR	1	0	4
Vicia kotschyana Boiss.	Fabaceae	EN	3	3067	12
Vicia kurdica Jalilian	Fabaceae	CR	1	0	4
Vicia sojakii Chrtkova-Zertova	Fabaceae	EN	3	74	12
Vincetoxicum assadii M. Zaeifi	Apocynaceae	CR	1	0	4
Vincetoxicum mozaffarianii M. Zaeifi	Apocynaceae	CR	1	0	4
Viola spathulata Willd.	Violaceae	VU	16	9806	60
Yazdana shirkuhensis A.Pirani & Noroozi	Caryophyllaceae	CR	2	0	4
Zeravschania aucheri (Boiss.) Pimenov	Apiaceae	LC or NT	36	640673	144

Zeravschania khorasanica Kljuykov & Lyskov	Apiaceae	CR	1	0	4
Zeravschania membranacea (Boiss.) M. Pimen.	Apiaceae	VU	8	152976	32
Zeravschania sola Lyskov & Kljuykov	Apiaceae	CR	1	0	4
Zerdana anchonioides Boiss.	Brassicaceae	VU	9	95748	36
Zeugandra iranica P.H.Davis	Campanulaceae	LC or NT	18	1437	72
Zhumeria majdae Rech.f. & Wendelbo	Lamiaceae	VU	13	164657	52
Zosima radians Boiss. & Hohen.	Apiaceae	VU	13	16893	44

The end...
